# Synthesis of β-ketophosphonates through aerobic copper(II)-mediated phosphorylation of enol acetates

**DOI:** 10.3762/bjoc.21.96

**Published:** 2025-06-20

**Authors:** Alexander S Budnikov, Igor B Krylov, Fedor K Monin, Valentina M Merkulova, Alexey I Ilovaisky, Liu Yan, Bing Yu, Alexander O Terent’ev

**Affiliations:** 1 N. D. Zelinsky Institute of Organic Chemistry of the Russian Academy of Sciences, 47 Leninsky prosp., 119991 Moscow, Russian Federation; 2 M. V. Lomonosov Moscow State University, 1 Leninskie Gory, 119991 Moscow, Russian Federation; 3 College of Chemistry, Zhengzhou University, Zhengzhou 450001, China; 4 Henan International Joint Laboratory of Rare Earth Composite Material, College of Materials Engineering, Henan University of Engineering, Zhengzhou 451191, China

**Keywords:** C–P coupling, copper, enol acetates, β-ketophosphonates, phosphorylation

## Abstract

Aerobic copper(II)-mediated phosphorylation of enol acetates with H-phosphonates leading to the formation of β-ketophosphonates was discovered. The proposed method is applicable to a wide range of H-phosphonates or phosphine oxides as PH-reagents and enol acetates. Unlike previous reports, which generally employed stoichiometric amounts of oxidants or more expensive transition metal catalysts, the present protocol employs only cheap copper sulfate pentahydrate as a catalyst under mild reaction conditions. The achieved phosphorylation proceeds via the formation of P-centered radicals produced by the oxidation of PH-reagents by copper(II)-containing species. Employing anhydrous CuSO_4_ instead of the pentahydrate led to a dramatic phosphorylation yield drop from 70 to <5%. It seems that the ligand environment of copper is very important for the effective reaction: other Cu(II) and Cu(I) salts, including halides, nitrate, tetrafluoroborate, or perchlorate, were much less effective or completely inert.

## Introduction

The construction of C–P bonds is a highly important task in key areas of modern chemistry [1–5] due to the numerous applications of phosphorus-containing compounds in pharmaceuticals, biology, agrochemistry, organic synthesis, and materials science [6–13].

**Scheme 1:**
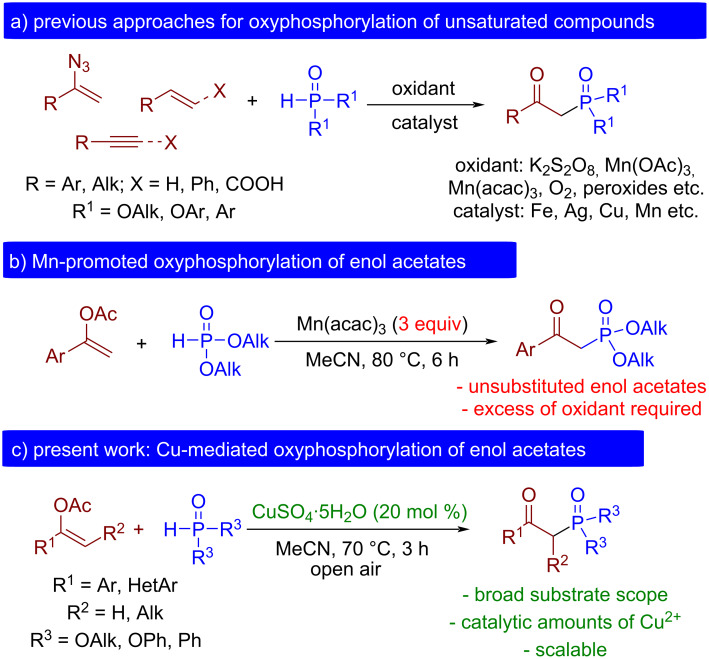
Recent approaches for the synthesis of β-ketophosphonates by the oxyphosphorylation of unsaturated compounds.

Among various organophosphorus compounds, β-ketophosphonates have received particular attention for various synthetically useful transformations, including alkene synthesis via Horner–Wadsworth–Emmons reaction [14–15], heterocycle construction [16–17], and the synthesis of chiral β-amino and β-hydroxy phosphonic acids [18–19]. Furthermore, they exhibit metal-complexing abilities [20], and anti-inflammatory [21–22] as well as enzyme inhibition activities [23–26]. Traditionally, β-ketophosphonates were prepared via Arbuzov reaction [27], acylation of alkylphosphonates [28], and hydration of alkynylphosphonates [29–31]. However, these methods have several drawbacks, including low atom efficiency, strong basic or acidic conditions, and excess of organohalides as starting materials. Recent years have witnessed the upsurge of free-radical oxidative phosphorylation transformations that became a reliable strategy for the construction of C–P bonds in organophosphorus chemistry [2,32–39]. The primary benefit of these reactions is introducing phosphorus fragments under mild reaction conditions into a diverse array of compounds that are inaccessible for functionalization employing other traditional approaches. After the pioneering work of Ji and Wei on aerobic oxyphosphorylation of styrenes [40], this strategy was further extended [41] to phenylacetylenes [42–44], cinnamic [45–48] and α,β-alkynyl carboxylic acids [42], and vinyl azides [49–50] (Scheme 1a). As a rule, transition metal catalysts (Fe [42,44,51–52], Cu [42,44,46,51,53], Ag [54], Mn [55], etc.) and strong oxidants (K_2_S_2_O_8_ [46,54], Mn(OAc)_3_ [56–57], organic peroxides [51,58–59], etc.) are employed in these approaches. Modern photocatalytic [47,50,60–61] and electrochemical [48,62] methods were also recently reported.

Although several successful oxyphosphorylation reactions leading to β-ketophosphonates have been reported [41–50,61], challenges in this area still exist primely in the search for new available synthetic equivalents of alkynes and alkenes for effective radical C–P bond formation. Enol acetates are potentially versatile precursors of α-substituted carbonyl compounds that have been recently applied as coupling partners for radical functionalization with the formation of C–C [63–66] and C–Het [67–70] bonds. The ready availability of enol acetates from the corresponding carbonyl compounds in just one synthetic step distinguishes them from the less accessible substituted vinylarenes, alkynes, cinnamic and α,β-alkynyl carboxylic acids, as well as vinyl azides. In addition, compared to similar silyl and alkyl enol ethers, enol acetates are more resistant to solvolysis, which prevents unwanted side reactions leading to the corresponding unfunctionalized carbonyl compounds. To date, only the Xu group reported oxidative phosphorylation of enol acetates with dialkyl H-phosphonates and Mn(acac)_3_ as an oxidant (Scheme 1b) [71]. However, the reported approach is limited by an excess of manganese salt, long reaction times, and scope limitations.

In the present work, the selective copper(II)-mediated phosphorylation of enol acetates with the formation of substituted β-ketophosphonates employing cheap copper sulfate as a catalyst and atmospheric oxygen as a terminal oxidant was carried out (Scheme 1c).

## Results and Discussion

We commenced our study with 1-phenylvinyl acetate (**1a**) and diisopropyl H-phosphonate (**2a**) as the model substrates by optimizing the reaction conditions (Table 1).

**Table 1:** Optimization of reaction conditions for the synthesis of β-ketophosphonate **3a** from enol acetate **1a** and diisopropyl H-phosphonate (**2a**)^a^.

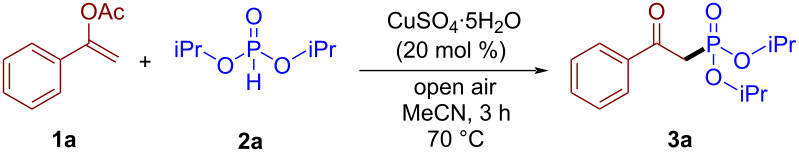		

Entry	Variations from standard reaction conditions	Yield **3a**, %^b^

**1**	**none**	**70 (68)**
2	without CuSO_4_·5H_2_O	n.d.
3	anhydrous CuSO_4_ (20 mol %)	<5
4	1/5/10 mol % of CuSO_4_·5H_2_O	24/56/64
5	rt, 24 h	9
6	DMF/DMSO/AcOH/toluene/EtOH instead of MeCN	<5
7	Cu(NO_3_)_2_·2.5H_2_O (20 mol %)	25
8	Cu(ClO_4_)_2_·6H_2_O (20 mol %)	23
9	Cu(BF_4_)_2_·xH_2_O (20 mol %)	39
10	CuBr_2_ (20 mol %)	n.d.
11	CuBr (20 mol %)	13
12	CuCl (20 mol %)	n.d.
13	CuCl_2_ (20 mol %)	n.d.
14	Cu(OAc)_2_ (20 mol %)	<5
15	Cu(acac)_2_ (20 mol %)	n.d.
16	Mn(acac)_3_ (20 mol %)	7
17	FeCl_3_ (20 mol %)	n.d.
18	CoSO_4_ (20 mol %)	n.d.
19	CoCO_3_ (20 mol %)	n.d.
20	AgNO_3_ (20 mol %)	11

^a^Standard reaction conditions: enol acetate **1a** (0.5 mmol, 81 mg), diisopropyl phosphite (**2a**, 1 mmol, 166 mg), catalyst (0–20 mol %, 0–37 mg), and a solvent (5 mL) were sequentially added to a round-bottom flask. The reaction mixture was stirred for 3 hours at 70 °C under air (air condenser); ^b^Yields of **3a** were determined by ^1^H NMR spectroscopy using 1,1,2,2-tetrachloroethane as an internal standard. The yield of isolated product is given in parenthesis.

After screening reaction conditions (see Table S1 in Supporting Information File 1 for more details) it was found that the best results were obtained employing 20 mol % of copper(II) sulfate pentahydrate and 2-fold excess of **2a** in MeCN at 70 °C under air atmosphere (Table 1, entry 1). In the absence of copper sulfate, no reaction product was detected (Table 1, entry 2). Notably, employing anhydrous copper sulfate afforded the desired product only in trace amounts (Table 1, entry 3). The optimal loading of the catalyst was examined (Table 1, entry 4); the best results were obtained with 20 mol % of copper sulfate, slightly lower yield (64%) was observed with 10 mol % loading of the catalyst. Increased temperature was crucial for the reaction output (Table 1, entry 5). The solvent screening revealed that MeCN is the best choice for the discovered transformation (Table 1, entry 6). Employing other copper salts as catalysts (Table 1, entries 7–15) resulted in lower yields compared to copper sulfate. In the case of copper(II) chloride and bromide (Table 1, entries 10 and 13) the formation of acetophenone as the main reaction product was observed. Presumably, in the presence of these salts, which can act as Lewis acids, the hydrolysis of the enol acetate **1a** proceeds faster than the generation of the corresponding P-centered radical from diisopropyl H-phosphonate (**2a**). In addition, the anion affects the redox properties of the copper ion, and thus the optimal choice is important to achieve both sufficient oxidative properties and catalyst recycling by reoxidation with O_2_. The employment of Mn(acac)_3_ and FeCl_3_, which had previously proven themselves effective in the oxyphosphorylation of styrenes [42,44,51–52] and enol acetates [71], turned out to be unsuitable in our case: the yield of **3a** did not exceed 7% (Table 1, entries 16 and 17). In the case of FeCl_3_ the formation of acetophenone as the main reaction product was observed. Other transition metal salts were also ineffective in the discovered transformation (Table 1, entries 18–20).

**Scheme 2:**
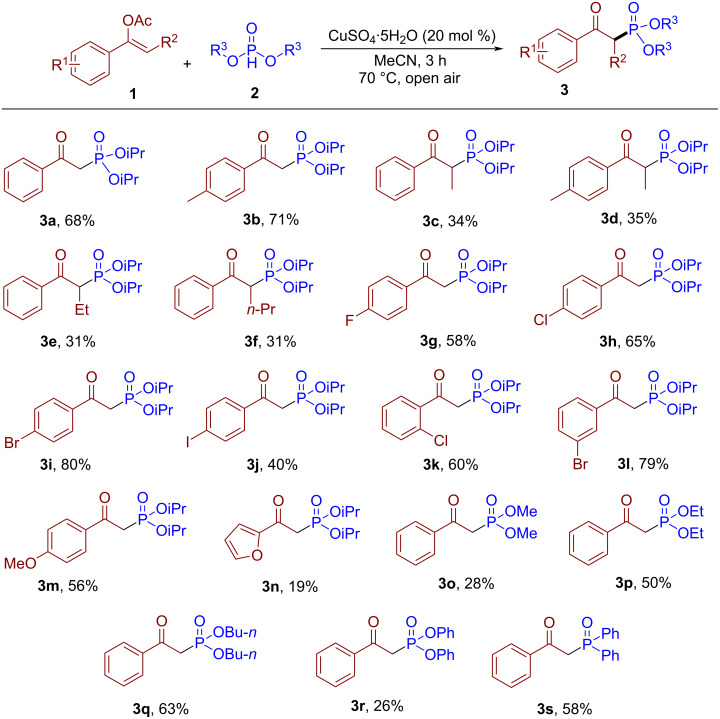
The scope of the discovered copper(II)-mediated phosphorylation of enol acetates.

With optimal conditions in hand, the scope of the developed phosphorylation protocol was evaluated (Scheme 2). The discovered phosphorylation is applicable to various substituted enol acetates **1a–n**, possessing both electron-donating and electron-withdrawing groups, leading to the desired phosphorylation products **3a–n** in good yields. Notably, sterically hindered terminal substituted enol acetates also furnished the desired products **3c–f**, albeit in lower yields. The substitution pattern in the aryl ring does not have a significant effect on the yield of **3** for both acceptor and donor derivatives, which shows the versatility of the developed protocol. However, among halogen-containing derivatives, higher yields were observed for bromine-substituted ones (products **3i** and **3l**, 80% and 79% yield). A furan moiety was also tolerated, giving the corresponding phosphorylated product **3n** in 19% yield.

Next, we evaluated the scope of the phosphorous component (products **3p–r**). The best result was obtained with di-*n*-butyl H-phosphonate (product **3q**). To our delight, diphenylphosphine oxide gave the corresponding product **3s** in 58% yield, although earlier **3s** wasn’t observed in an analogous reaction when Mn(acac)_3_ was used as an oxidant [71].

**Scheme 3:**
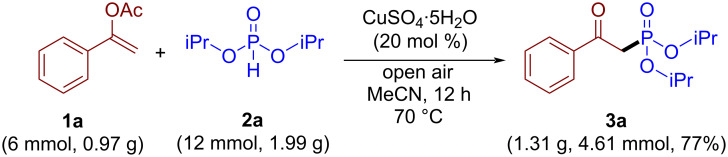
Gram-scale synthesis of **3a**.

The synthetic utility of the developed protocol was demonstrated by performing the model reaction on a 6 mmol scale (Scheme 3), product **3a** was isolated in 77% yield (1.3 g, 4.61 mmol).

**Scheme 4:**
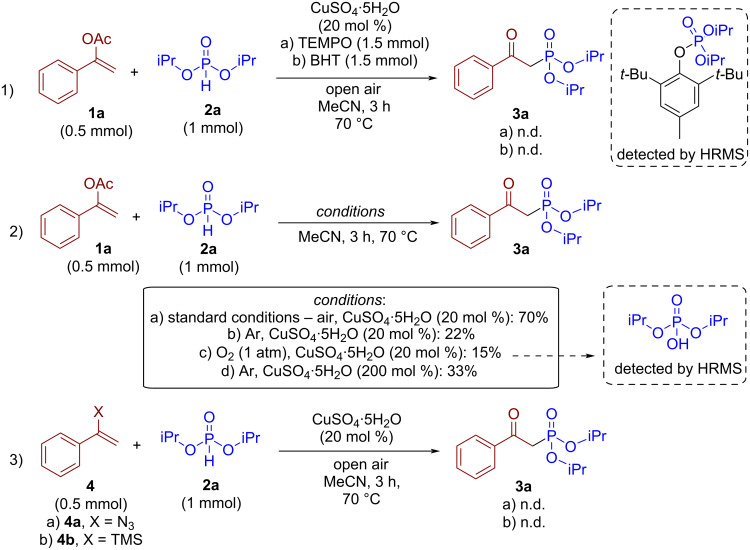
Control experiments.

In order to propose the reaction mechanism, control experiments were conducted (Scheme 4).

The reaction is completely inhibited by the addition of (2,2,6,6-tetramethylpiperidin-1-yl)oxyl (TEMPO) or BHT (Scheme 4, reaction 1). A BHT-adduct derived from a P-centered radical was detected by HRMS, which supported a radical mechanism. Next, the influence of the atmosphere and the role of copper catalyst was evaluated (Scheme 4, reaction 2). Neither under argon nor under an oxygen atmosphere, the yield of **3a** did not exceed 22%. However, employing 200 mol % of copper(II) sulfate under an argon atmosphere afforded **3a** in 33% yield. These results indicate, that copper(II) at high loading is capable of oxidizing H-phosphonate even in inert conditions [72]; however, a balanced concentration of oxygen in the reaction mixture is required for recycling of copper catalyst and effective generation of phosphorous radicals. Under a pure O_2_ atmosphere, the interception of formed P-centered radicals by excess oxygen can presumably inhibit the target process by the formation of unreactive phosphoric acid from the corresponding H-phosphonate [73–74]. The formation of phosphoric acid from diisopropyl H-phosphonate (**2a**) was confirmed by HRMS analysis of the crude reaction mixture. Finally, vinyl azide **4a** and silyl enol ether **4b** were introduced into standard reaction conditions (Scheme 4, reaction 3). However, no phosphorylation product **3a** was observed.

**Scheme 5:**
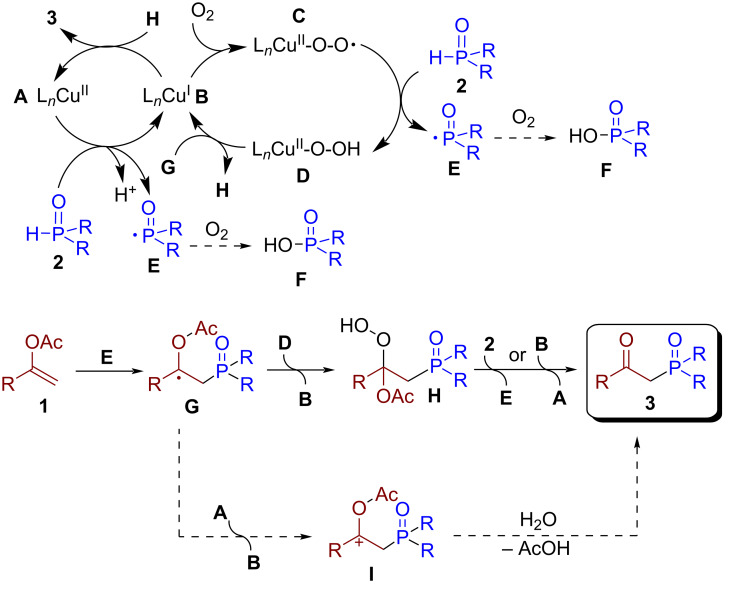
Proposed mechanism for copper(II) mediated phosphorylation of enol acetates.

On the basis of the obtained results and previous reports on copper(II) mediated oxyphosphorylation reactions [42,44,46,51,53], a plausible reaction mechanism is proposed (Scheme 5).

The discovered transformation is unlikely to proceed via only a single route (see control experiments in Scheme 4), and the generation of phosphorus radicals and product **3** can be described by several pathways. The direct oxidation of **2** by Cu(II) **A** leads to the formation of P-centered radical **E** and Cu(I) **B**. Under air atmosphere, the formed Cu(I) species **B** can react with molecular oxygen resulting in the formation of peroxycopper intermediate **C** [75–79]. The latter abstracts hydrogen atom from H-phosphonate or phosphine oxide **2**, thus generating P-centered radical **E** and hydroperoxide complex **D**. Subsequently, the addition of **E** to the double bond of enol acetate **1** leads to the formation of benzylic radical **G**. The latter undergoes hydroperoxide transfer from copper complex **D** with the formation of intermediate **H** and, therefore, regenerating Cu(I) **B**. Alternatively, the latter can be formed by oxidation of benzylic radical **G** with the formation of carbocation **I**. However, given that in the absence of oxygen the β-ketophosphonate was formed only in 33% yield (Scheme 4, reaction 2d), the direct oxidation of **G** to **I** is unlikely to be the main reaction pathway. Finally, the reduction of hydroperoxide **H** by initial phosphorous precursor **2** [80–82] or by Cu(I) **B** delivers β-ketophosphonate **3** and P-centered radical **E** or regenerated Cu(II) **A**. Alternatively, **3** can be formed by hydrolysis of benzylic cation **I** [71].

## Conclusion

In summary, we have disclosed an aerobic copper(II)-mediated phosphorylation of enol acetates with H-phosphonates leading to substituted β-ketophosphonates. The suggested method is versatile and can also be applied to phosphine oxides as PH-reagents. The developed protocol utilizes a simple copper catalyst under mild reaction conditions and synthetically available enol acetates (compared to traditionally employed alkenes, alkynes, cinnamic, and α,β-alkynyl carboxylic acids). The present approach showed good functional group tolerance and can be applied for the gram-scale synthesis of target compounds. Conducted mechanistic studies revealed that the discovered transformation proceeds via the formation of P-centered radicals produced by the oxidation of the corresponding phosphorous precursors by copper(II)-containing species.

## Experimental

**General**: In all experiments rt stands for 22–25 °C. ^1^H and ^13^C NMR spectra were recorded on Bruker AVANCE II 300, Bruker Fourier 300HD (300.13 MHz for ^1^H, 75.47 MHz for ^13^C and 121.49 MHz for ^31^P, respectively), and Bruker AVANCE II 600 (600.13 MHz for ^1^H, 150.92 MHz for ^13^C, 242.93 MHz for ^31^P) spectrometers in CDCl_3_. Chemical shifts were reported in parts per million (ppm), and the residual solvent peak was used as an internal reference: ^1^H (СDCl_3_ δ = 7.26 ppm), ^13^C (СDCl_3_ δ = 77.16 ppm). Multiplicity was indicated as follows: s (singlet), d (doublet), t (triplet), q (quartet), m (multiplet). Coupling constants are reported in Hertz (Hz).

FTIR spectra were recorded on a Bruker Alpha instrument. High-resolution mass spectra (HRMS) were measured on a Bruker maXis instrument using electrospray ionization (ESI). The measurements were performed in a positive ion mode (interface capillary voltage – 4500 V); mass range from *m*/*z* 50 to *m*/*z* 3000 Da; external calibration with Electrospray Calibrant Solution (Fluka). A syringe injection was used for all acetonitrile solutions (flow rate 3 μL/min). Nitrogen was applied as a dry gas; interface temperature was set at 180 °C.

### General reaction conditions for copper(II)-mediated phosphorylation of enolacetates (experimental data for Scheme 2)

Enol acetate **1** (0.5 mmol, 81–121 mg), H-phosphonate **2** (1.0 mmol, 110–234 mg), CuSO_4_·5H_2_O (0.1 mmol, 25 mg), and MeCN (5 mL) were sequentially added to a round-bottom flask. The reaction mixture (suspension) was stirred for 3 hours at 70 °C under air (air condenser) and then cooled to room temperature, and rotary-evaporated under reduced pressure. An additional evaporation step using a rotary vane pump (0.5 mmHg) at 80 °C was made for the evaporation of phosphite excess. The residue was isolated by column chromatography on silica gel.

## Supporting Information

File 1 Experimental details, compound characterization data, and NMR spectra.

## Data Availability

All data that supports the findings of this study is available in the published article and/or the supporting information of this article.
