# Supplementary material for: Synthesis of β-ketophosphonates through aerobic copper(II)-mediated phosphorylation of enol acetates
Source: Beilstein J Org Chem. 2025 Jun 20;21:1192–200. doi: 10.3762/bjoc.21.96 (PMC12207267; doi:10.3762/bjoc.21.96)
Supplement: File 1 — Experimental details, compound characterization data, and NMR spectra. [file Beilstein_J_Org_Chem-21-1192-s001.pdf]

BEILSTEIN JOURNAL OF ORGANIC CHEMISTRY

Supporting Information

for

Synthesis of β-ketophosphonates through aerobic copper(II)-mediated phosphorylation of enol acetates

Alexander S. Budnikov, Igor B. Krylov, Fedor K. Monin, Valentina M. Merkulova, Alexey I. Ilovaisky, Liu Yan, Bing Yu and Alexander O. Terent’ev

Beilstein J. Org. Chem. **2025,** *21,* 1192–1200. doi:10.3762/bjoc.21.96

Experimental details, compound characterization data, and NMR spectra

License and Terms: This is a supporting information file under the terms of the Creative Commons Attribution License (https://creativecommons.org/licenses/by/4.0). Please note that the reuse, redistribution and reproduction in particular requires that the author(s) and source are credited and that individual graphics may be subject to special legal provisions.
The license is subject to the *Beilstein Journal of Organic Chemistry* terms and conditions: (https://www.beilstein-journals.org/bjoc/terms)

Table of contents

General S3

Experimental details and characterization data of synthesized compounds S4

References..S13

The ^{1}H, ^{13}C, and ^{31}P spectra of synthesized compounds..S14

General

In all experiments rt stands for 22–25 °C. ^{1}H and ^{13}C NMR spectra were recorded on a Bruker AVANCE II 300, Bruker Fourier 300HD (300.13 MHz for ^{1}H, 75.47 MHz for ^{13}C and 121.49 MHz for ^{31}P, respectively) and Bruker AVANCE II 600 (600.13 MHz for ^{1}H, 150.92 MHz for ^{13}C, 242.93 MHz for ^{31}P) spectrometers in $CDCl_3$. Chemical shifts were reported in parts per million (ppm), and the residual solvent peak was used as an internal reference: ^{1}H ($CDCl_3$ δ = 7.26 ppm), ^{13}C ($CDCl_3$ δ = 77.16 ppm). Multiplicity was indicated as follows: s (singlet), d (doublet), t (triplet), q (quartet), m (multiplet). Coupling constants were reported in Hertz (Hz).

FTIR spectra were recorded on Bruker Alpha instrument. High resolution mass spectra (HRMS) were measured on a Bruker maXis instrument using electrospray ionization (ESI). The measurements were performed in a positive ion mode (interface capillary voltage – 4500 V); mass range from *m*/*z* 50 to *m*/*z* 3000 Da; external calibration with Electrospray Calibrant Solution (Fluka). A syringe injection was used for all acetonitrile solutions (flow rate 3 μL/min). Nitrogen was applied as a dry gas; interface temperature was set at 180 °C.

Diisopropyl phosphite 98%, diethyl phosphite 98%, dimethyl phosphite 99%, dibutyl phosphite 98%, diphenyl phosphite 98%, diphenylphosphine oxide 97%, 2,2,6,6-tetramethylpiperidoxyl (TEMPO) 99%, 3,5-di-*tert*-butyl-4-hydroxytoluene (BHT) 99%, manganese(III) acetylacetonate 98%, copper(II) sulfate pentahydrate 98%, copper(II) sulfate 99%, copper(I) bromide 99%, copper(II) bromide 99%, copper(II) nitrate hemi(pentahydrate) 99%, copper(II) perchlorate hexahydrate 98%, copper(II) tetrafluoroborate hydrate 98%, copper(II) acetylacetonate 99%, iron(III) chloride 97%, copper(I) chloride 99%, copper(II) chloride 97%, copper(II) acetate 98%, copper(II) acetate monohydrate 98%, cobalt(II) sulfate heptahydrate 99%, cobalt(II) carbonate hydrate 99%, silver nitrate 99% were used as is from commercial sources. Acetonitrile and ethyl acetate were distilled over P_2O_5 prior use. Toluene, DMSO, and DMF were distilled over CaH_2 prior to use. Glacial acetic acid was used as is from commercial sources. Enol acetates **1a–n**, vinyl azide **4a**, and silyl enol ether **4b** were prepared according literature procedures[1–6].

Experimental details and characterization data of synthesized compounds

Optimization of the reaction conditions (additional experimental data for Table 1).

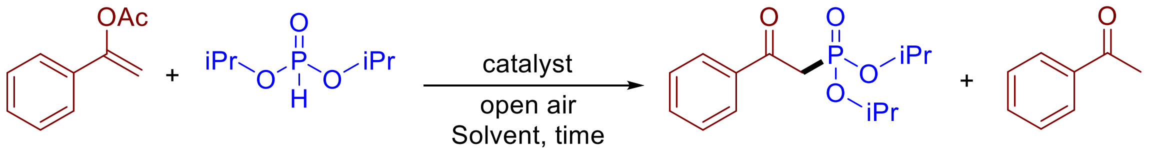

1a **2a** **3a** **A**

Run	Molar ratio **1a**:**2a**	catalyst, mol %	Solvent	Time, h	Yield %,[a] **3a**	Yield %,[a] **A**
1	1:4	$CuSO_4$•$5H_2O$, 20	MeCN	10	62	7
2	1:1.5	$CuSO_4$•$5H_2O$, 20	MeCN	10	56	8
3	1:1.5	$CuSO_4$•$5H_2O$, 20	MeCN	6	52	4
4	1:2	$CuSO_4$•$5H_2O$, 20	MeCN	6	71	10
5	1:2	none	MeCN	6	n.d.	n.d.
6	1:2	CuBr, 20	MeCN	6	13	9
7	1:2	$CuBr_2$, 20	MeCN	6	n.d.	81
8	1:2	CuCl, 20	MeCN	6	n.d	7
9	1:2	$CuCl_2$, 20	MeCN	6	n.d.	81
10	1:2	$Cu(NO_3)_2$•$2.5H_2O$, 20	MeCN	6	25	<5
11	1:2	$Cu(ClO_4)_2$•$6H_2O$, 20	MeCN	6	23	34
12	1:2	$Cu(BF_4)_2$•xH_2O, 20	MeCN	6	39	25
13	1:2	$CuSO_4$, 20	MeCN	6	<5	<5
14	1:2	$Cu(acac)_2$, 20	MeCN	6	n.d.	7
15	1:2	$Cu(OAc)_2$, 20	MeCN	6	<5	<5
16	1:2	$Cu(OAc)_2$•H_2O, 20	MeCN	6	<5	<5
17	1:2	$CoSO_4$, 20	MeCN	6	n.d.	5
18	1:2	$CoCO_3$, 20	MeCN	6	n.d.	4
19	1:2	$Mn(acac)_3$, 20	MeCN	6	7	7
20	1:2	$AgNO_3$, 20	MeCN	6	11	<5
21	1:2	$FeCl_3$, 20	MeCN	6	n.d.	74
22	1:2	$CuSO_4$•$5H_2O$, 20	DMF	6	<5	<5
23	1:2	$CuSO_4$•$5H_2O$, 20	AcOH	6	n.d.	12
24	1:2	$CuSO_4$•$5H_2O$, 20	DMSO	6	<5	<5
25	1:2	$CuSO_4$•$5H_2O$, 20	Toluene	6	<5	<5
26	1:2	$CuSO_4$•$5H_2O$, 20	EtOH	6	<5	<5
27	1:2	$CuSO_4$•$5H_2O$, 20	MeCN/H_2O = 4/1	3	32	6
**28**	**1:2**	**$CuSO_4$•$5H_2O$, 20**	**MeCN**	**3**	**70 (68)**	**<5**
29	1:2	$CuSO_4$•$5H_2O$, 20	MeCN	1	36	<5
30[b]	1:2	$CuSO_4$•$5H_2O$, 20	MeCN	24	9	5
31	1:2	$CuSO_4$•$5H_2O$, 10	MeCN	3	64	<5
32	1:2	$CuSO_4$•$5H_2O$, 5	MeCN	3	56	<5
33	1:2	$CuSO_4$•$5H_2O$, 1	MeCN	12	24	<5
34[c]	1:2	$CuSO_4$•$5H_2O$, 20	MeCN	3	71	<5

[a]Yields were determined by ^{1}H NMR using 1,1,2,2-tetrachloroethane as an internal standard. The yields for the isolated products are given in parenthesis. [b]rt. [c]Finely powdered $CuSO_4$•$5H_2O$.

Enol acetate **1a** (0.5 mmol, 81 mg), diisopropyl phosphite (**2a**, 0.5–2 mmol, 83–332 mg), catalyst (0–20 mol %, 0–37 mg), and a solvent (5 mL) were sequentially added to a round-bottom

flask. The reaction mixture was stirred for 3 hours at 70 °C under air (unless otherwise stated), cooled to room temperature, and rotary-evaporated under reduced pressure. The yields of **3a** were determined by ^{1}H NMR spectroscopy using 1,1,2,2-tetrachloroethane as an internal standard. In the run 28 **3a** was isolated by column chromatography on silica gel using $CHCl_3$/MeOH = 60:1 mixture as an eluent.

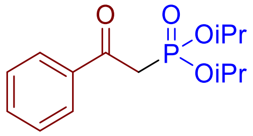

Diisopropyl (2-oxo-2-phenylethyl)phosphonate (3a) was synthesized as yellow oil (68%, isolated by column chromatography using $CHCl_3$/MeOH = 60/1 as an eluent).[5] **^{1}H NMR** (300.13 MHz, $CDCl_3$): δ = 8.04 – 7.94 (m, 2H), 7.60 – 7.50 (m, 1H), 7.49 – 7.39 (m, 2H), 4.88 – 4.52 (m, 2H), 3.57 (d, *J* = 22.9 Hz, 2H), 1.26 (d, *J* = 2.2 Hz, 6H), 1.24 (d, *J* = 2.2 Hz, 6H). **^{13}C NMR** (75.48 MHz, $CDCl_3$): δ = 192.2 (d, *J* = 6.7 Hz), 136.8, 133.6, 129.2, 128.6, 71.6 (d, *J* = 6.5 Hz), 39.9 (d, *J* = 130.2 Hz), 24.0 (d, *J* = 3.6 Hz), 23.8 (d, *J* = 5.0 Hz). **^{31}P** (121.49 MHz, $CDCl_3$): δ = 18.6.

General reaction conditions for copper(II)-mediated phosphorylation of enolacetates (experimental data for Scheme 2).

Enol acetate **1** (0.5 mmol, 81–121 mg), phosphite **2** (1.0 mmol, 110–234 mg), $CuSO_4{\cdot}5H_2O$ (0.1 mmol, 25 mg), and MeCN (5 mL) were sequentially added to a round-bottom flask. The reaction mixture (suspension) was stirred for 3 hours at 70 °C under air (air condenser) and then cooled to room temperature, and rotary-evaporated under reduced pressure. An additional evaporation step using a rotary vane pump (0.5 mmHg) at 80 °C was made for the evaporation of phosphite excess. The residue was isolated by column chromatography on silica gel (eluent is given for each product, see below).

^{1}H, ^{13}C, and ^{31}P NMR spectra of the synthesized compounds **3a–c,g–l,m** were in agreement with the literature data.[1,5,6]

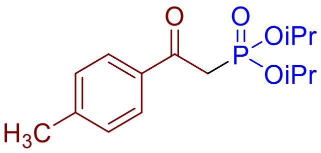

Diisopropyl (2-oxo-2-(*p*-tolyl)ethyl)phosphonate (3b) was synthesized as yellow oil (71%, isolated by column chromatography using $CHCl_3$/MeOH = 60/1 as an eluent).[5] **^{1}H NMR** (300.13 MHz, $CDCl_3$): δ = 7.91 (d, *J* = 8.2 Hz, 2H), 7.25 (d, *J* = 8.2 Hz, 2H), 4.87–4.55 (m, 2H), 3.55 (d, *J* = 22.9 Hz, 2H), 2.40 (s, 3H), 1.28 (d, *J* = 2.7 Hz, 6H), 1.26 (d, *J* = 2.7 Hz, 6H). **^{13}C NMR** (75.47 MHz, $CDCl_3$): δ = 191.8 (d, *J* = 6.7 Hz), 144.6, 134.4 (d, *J* = 1.7 Hz), 129.4, 129.3, 71.5 (d, *J* =

6.7 Hz), 39.8 (d, J = 130.4 Hz), 24.1 (d, J = 3.8 Hz), 23.9 (d, J = 5.2 Hz), 21.8. **^{31}P** (121.49 MHz, $CDCl_3$): δ = 18.0.

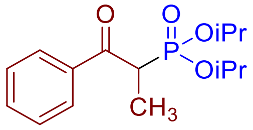

Diisopropyl (1-oxo-1-phenylpropan-2-yl)phosphonate (3c) was synthesized as pale yellow oil (34%, isolated by column chromatography using DCM/EA = 3/1 as an eluent).[5] **^{1}H NMR** (300.13 MHz, $CDCl_3$): δ = 8.09 – 7.95 (m, 2H), 7.62 – 7.50 (m, 1H), 7.49 – 7.40 (m, 2H), 4.82 – 4.51 (m, 2H), 4.11 (dq, J = 23.6, 7.0 Hz, 1H), 1.50 (dd, J = 18.0, 7.0 Hz, 3H), 1.29 (d, J = 6.2 Hz, 3H), 1.25 (d, J = 6.2 Hz, 3H), 1.22 (d, J = 6.2 Hz, 3H), 1.17 (d, J = 6.2 Hz, 3H). **^{13}C NMR** (75.50 MHz, $CDCl_3$): δ = 196.8 (d, J = 4.9 Hz), 137.2, 133.3, 129.0, 128.5, 71.5 (d, J = 2.2 Hz), 71.4 (d, J = 2.2 Hz), 42.0 (d, J = 131.2 Hz), 24.2 (d, J = 3.5 Hz), 24.1 (d, J = 3.5 Hz), 23.8 (d, J = 5.2 Hz), 12.3 (d, J = 6.5 Hz). **^{31}P** (121.54 MHz, $CDCl_3$): δ = 21.3.

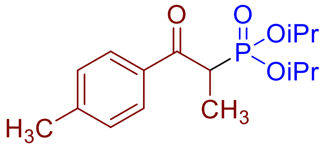

Diisopropyl (1-oxo-1-(*p*-tolyl)propan-2-yl)phosphonate (3d) was synthesized as pale yellow oil (35%, isolated by column chromatography using $CHCl_3$/MeOH = 60/1 as an eluent). **^{1}H NMR** (300.23 MHz, $CDCl_3$): δ = 7.89 (d, J = 8.2 Hz, 2H), 7.24 (d, J = 8.2 Hz, 2H), 4.81 – 4.53 (m, 2H), 4.08 (dq, J = 23.4, 7.0 Hz, 1H), 2.39 (s, 3H), 1.48 (dd, J = 18.0, 7.0 Hz, 3H), 1.29 (d, J = 6.2 Hz, 3H), 1.26 (d, J = 6.2 Hz, 3H), 1.23 (d, J = 6.2 Hz, 3H), 1.18 (d, J = 6.2 Hz, 3H). **^{13}C NMR** (75.50 MHz, $CDCl_3$): δ = 196.3 (d, J = 4.8 Hz), 144.1, 134.7 (d, J = 1.1 Hz), 129.20, 129.19, 71.3 (d, J = 7.0 Hz), 41.9 (d, J = 131.4 Hz), 24.2 (d, J = 3.3 Hz), 24.1 (d, J = 3.3 Hz), 23.9 (d, J = 5.2 Hz), 21.8, 12.4 (d, J = 6.4 Hz). **^{31}P** (121.54 MHz, $CDCl_3$): δ = 21.6. **FT-IR** (thin layer): ν_{max} = 2980, 2937, 1679, 1607, 1454, 1384, 1324, 1301, 1251, 1181, 1107, 987, 944, 786. **HR-MS** (ESI): m/z = 335.1383, calc. for $C_{16}H_{25}O_4P+Na^+$: 335.1382.

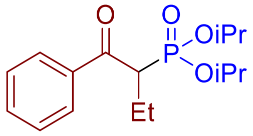

Diisopropyl (1-oxo-1-phenylbutan-2-yl)phosphonate (3e) was synthesized as pale yellow oil (31%, isolated by column chromatography using DCM/EA = 3/1 with gradient elution to 1/1). **^{1}H NMR** (300.13 MHz, $CDCl_3$): δ = 8.05 – 7.93 (m, 2H), 7.58 – 7.49 (m, 1H), 7.49 – 7.38 (m, 2H), 4.81 – 4.49 (m, 2H), 3.96 (ddd, J = 23.2, 10.5, 3.7 Hz, 1H), 2.36 – 2.11 (m, 1H), 2.10 – 1.91 (m, 1H), 1.28 (d, J = 6.2 Hz, 3H), 1.25 (d, J = 6.2 Hz, 3H), 1.19 (d, J = 6.2 Hz, 3H), 1.15 (d, J = 6.2

Hz, 3H), 0.91 (t, *J* = 7.3 Hz, 3H). **^{13}C NMR** (75.47 MHz, $CDCl_3$): δ = 196.7 (d, *J* = 5.3 Hz), 138.3, 133.2, 128.8, 128.5, 71.41 (d, *J* = 6.5 Hz), 71.33 (d, J = 6.5 Hz), 49.8 (d, *J* = 129.3 Hz), 24.2 (d, *J* = 3.2 Hz), 24.0 (d, *J* = 3.2 Hz), 23.8 (d, *J* = 5.1 Hz), 21.3 (d, *J* = 4.8 Hz), 13.3 (d, *J* = 16.0 Hz). **^{31}P** (121.54 MHz, $CDCl_3$): δ = 20.4. **HR-MS** (ESI): *m/z* = 313.1563, calc. for $C_{16}H_{25}O_4P+H^+$: 313.1563. **FT-IR** (thin layer): ν_{max} = 2979, 2936, 1681, 1449, 1385, 1267, 1250, 1220, 1179, 1106, 1013, 986, 774, 691, 553.

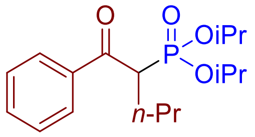

Diisopropyl (1-oxo-1-phenylpentan-2-yl)phosphonate (3f) was synthesized as yellow oil (31%, isolated by column chromatography using DCM/EA = 3/1 as an eluent). **^{1}H NMR** (300.13 MHz, $CDCl_3$): δ = 8.04 – 7.92 (m, 2H), 7.59 – 7.50 (m, 1H), 7.50 – 7.39 (m, 2H), 4.82 – 4.51 (m, 2H), 4.06 (ddd, *J* = 23.5, 10.7, 3.5 Hz, 1H), 2.35 – 2.07 (m, 1H), 2.02 – 1.77 (m, 1H), 1.41 – 1.31 (m, 2H), 1.29 (d, *J* = 6.2 Hz, 3H), 1.26 (d, *J* = 6.2 Hz, 3H), 1.20 (d, *J* = 6.2 Hz, 3H), 1.15 (d, *J* = 6.2 Hz, 3H), 0.87 (t, *J* = 7.3 Hz, 3H). **^{13}C NMR** (75.47 MHz, $CDCl_3$): δ = 196.7 (d, *J* = 5.3 Hz), 138.20, 133.2, 128.8, 128.5, 71.46 (d, *J* = 7.6 Hz), 71.35 (d, J = 7.6 Hz), 47.9 (d, *J* = 129.3 Hz), 29.8 (d, *J* = 5.0 Hz), 24.2 (d, *J* = 3.5 Hz), 24.1 (d, *J* = 3.5 Hz), 23.8 (d, *J* = 5.4 Hz), 22.0 (d, *J* = 15.5 Hz), 14.0. **^{31}P** (121.49 MHz, $CDCl_3$): δ = 21.4. **HR-MS** (ESI): *m/z* = 344.1985, calc. for $C_{17}H_{27}O_4P+NH_4^+$: 344.1983. **FT-IR** (thin layer): ν_{max} = 2978, 2934, 1681, 1449, 1385, 1250, 1209, 1178, 1107, 986, 771, 552.

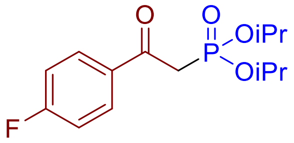

Diisopropyl (2-(4-fluorophenyl)-2-oxoethyl)phosphonate (3g) was synthesized as yellow oil (58%, isolated by column chromatography using $CHCl_3$/MeOH = 60/1 as an eluent).[5] **^{1}H NMR** (300.13 MHz, $CDCl_3$): δ = 8.13 – 7.93 (m, 2H), 7.19–7.01 (m, 2H), 4.79–4.56 (m, 2H), 3.53 (d, *J* = 22.9 Hz, 2H), 1.26 (d, *J* = 1.9 Hz, 6H), 1.24 (d, *J* = 1.9 Hz, 6H). **^{13}C NMR** (75.470 MHz, $CDCl_3$): δ = 190.5 (d, *J* = 6.5 Hz), 166.1 (d, *J* = 255.7 Hz), 133.2 (d, *J* = 2.5 Hz), 132.0 (d, *J* = 9.5 Hz), 115.7 (d, *J* = 22.0 Hz), 71.7 (d, *J* = 6.6 Hz), 40.0 (d, *J* = 129.7 Hz), 24.0 (d, *J* = 3.7 Hz), 23.8 (d, *J* = 5.0 Hz). **^{31}P** (121.54 MHz, $CDCl_3$): δ = 17.5.

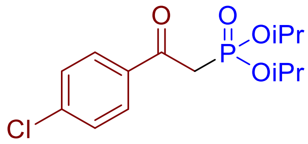

Diisopropyl (2-(4-chlorophenyl)-2-oxoethyl)phosphonate (3h) was synthesized as yellow oil (65%, isolated by column chromatography using $CHCl_3$/MeOH = 80/1 with gradient elution to 60/1).[5] **^{1}H NMR** (300.13 MHz, $CDCl_3$): δ = 7.96 (d, *J* = 8.6 Hz, 2H), 7.43 (d, *J* = 8.6 Hz, 2H), 4.82 – 4.59 (m, 2H), 3.54 (d, *J* = 23.0 Hz, 2H), 1.27 (d, *J* = 1.5 Hz, 6H), 1.25 (d, *J* = 1.5 Hz, 6H). **^{13}C NMR** (75.47 MHz, $CDCl_3$): δ = 191.0 (d, *J* = 6.7 Hz), 140.2, 135.1, 130.7, 128.9, 71.7 (d, *J* = 6.7 Hz), 40.1 (d, *J* = 129.5 Hz), 24.1 (d, *J* = 3.9 Hz), 23.9 (d, *J* = 5.0 Hz). **^{31}P** (121.49 MHz, $CDCl_3$): δ = 18.1.

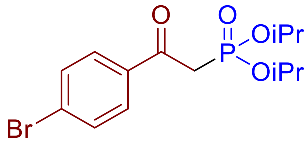

Diisopropyl (2-(4-bromophenyl)-2-oxoethyl)phosphonate (3i) was synthesized as pale yellow oil (80%, isolated by column chromatography using DCM/EA = 3/1 as an eluent).[5] **^{1}H NMR** (300.13 MHz, $CDCl_3$): δ = 7.88 (d, *J* = 8.6 Hz, 2H), 7.59 (d, *J* = 8.6 Hz, 2H), 4.83 – 4.60 (m, 2H), 3.53 (d, *J* = 23.0 Hz, 2H), 1.27 (d, *J* = 1.6 Hz, 6H), 1.25 (d, *J* = 1.6 Hz, 6H). **^{13}C NMR** (75.47 MHz, $CDCl_3$): δ = 191.2 (d, *J* = 6.6 Hz), 135.5, 131.9, 130.8, 129.0, 71.7 (d, *J* = 6.6 Hz), 40.1 (d, *J* = 129.4 Hz), 24.1 (d, *J* = 3.6 Hz), 23.9 (d, *J* = 4.9 Hz). **^{31}P** (121.54 MHz, $CDCl_3$): δ = 17.2.

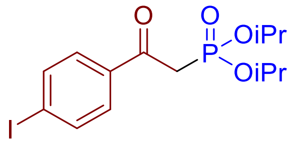

Diisopropyl (2-(4-iodophenyl)-2-oxoethyl)phosphonate (3j) was synthesized as yellow oil (40%, isolated by column chromatography using $CHCl_3$/MeOH = 60/1 as an eluent). **^{1}H NMR** (300.13 MHz, $CDCl_3$): δ = 7.82 (d, *J* = 8.6 Hz, 2H), 7.71 (d, *J* = 8.6 Hz, 2H), 4.83 – 4.55 (m, 2H), 3.52 (d, *J* = 23.0 Hz, 2H), 1.27 (d, *J* = 1.8 Hz, 6H), 1.25 (d, *J* = 1.8 Hz, 6H). **^{13}C NMR** (75.47 MHz, $CDCl_3$): δ = 191.5 (d, *J* = 6.7 Hz), 137.9, 136.0 (d, *J* = 1.4 Hz), 130.6, 101.9, 71.7 (d, *J* = 6.7 Hz), 39.9 (d, *J* = 129.5 Hz), 24.1 (d, *J* = 3.9 Hz), 23.9 (d, *J* = 5.2 Hz). **^{31}P** (121.54 MHz, $CDCl_3$): δ = 17.2. **HR-MS** (ESI): *m/z* = 433.0036, calc. for $C_{14}H_{20}IO_4P+Na^+$: 433.0038. **FT-IR** (thin layer): ν_{max} = 2928, 1727, 1678, 1646, 1581, 1563, 1384, 1270, 1182, 988, 802, 741, 703.

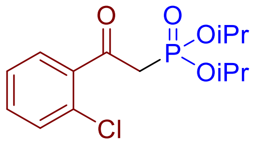

Diisopropyl (2-(2-chlorophenyl)-2-oxoethyl)phosphonate (3k) was synthesized as pale yellow oil (60%, isolated by column chromatography using $CHCl_3$/MeOH = 60/1 as an eluent). **^{1}H NMR** (300.23 MHz, $CDCl_3$): δ = 7.59 – 7.52 (m, 1H), 7.43 – 7.37 (m, 2H), 7.36 – 7.27 (m, 1H), 4.84 – 4.55 (m, 2H), 3.66 (d, *J* = 22.5 Hz, 2H), 1.25 (d, *J* = 3.3 Hz, 6H), 1.23 (d, *J* = 3.3 Hz, 6H). **^{13}C NMR** (75.50 MHz, $CDCl_3$): δ = 194.7 (d, *J* = 7.0 Hz), 139.0, 132.2, 131.2, 130.5, 130.2, 127.0, 71.6 (d, *J* = 6.7 Hz), 43.6 (d, *J* = 128.7 Hz), 24.0 (d, *J* = 3.9 Hz), 23.8 (d, *J* = 5.1 Hz). **^{31}P** (121.54 MHz, $CDCl_3$): δ = 16.9. **HR-MS** (ESI): *m/z* = 357.0419, calc. for $C_{14}H_{20}ClO_4P+K^+$: 357.0416. **FT-IR** (thin layer): ν_{max} = 2981, 2932, 1698, 1590, 1435, 1385, 1287, 1258, 1180, 1106, 1018, 989, 777, 760, 737.

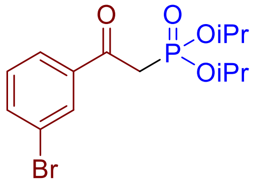

Diisopropyl (2-(3-bromophenyl)-2-oxoethyl)phosphonate (3l) was synthesized as yellow oil (79%, isolated by column chromatography using $CHCl_3$/MeOH = 60/1 as an eluent). **^{1}H NMR** (300.13 MHz, $CDCl_3$): δ = 8.14 (t, *J* = 1.9 Hz, 1H), 7.94 (dt, *J* = 8.0, 1.4 Hz, 1H), 7.69 (dt, *J* = 8.0, 1.4 Hz, 1H), 7.34 (t, *J* = 8.0 Hz, 1H), 4.83 – 4.60 (m, 2H), 3.55 (d, *J* = 23.0 Hz, 2H), 1.28 (d, *J* = 2.2 Hz, 6H), 1.26 (d, *J* = 2.2 Hz, 6H). **^{13}C NMR** (75.47 MHz, $CDCl_3$): δ = 190.9 (d, *J* = 6.8 Hz), 138.4 (d, *J* = 1.2 Hz), 136.4, 132.3, 130.2, 127.9, 122.9, 71.8 (d, *J* = 6.8 Hz), 40.1 (d, *J* = 129.6 Hz), 24.1 (d, *J* = 3.9 Hz), 23.9 (d, *J* = 5.2 Hz). **^{31}P** (121.54 MHz, $CDCl_3$): δ = 16.9. **HR-MS** (ESI): *m/z* = 380.0621, 382.0601, calc. for $C_{14}H_{20}BrO_4P+NH_4^+$: 380.0628, 382.0615. **FT-IR** (thin layer): ν_{max} = 2980, 2933, 1686, 1566, 1385, 1255, 1199, 1179, 1141, 1106, 987, 888, 792, 782, 590.

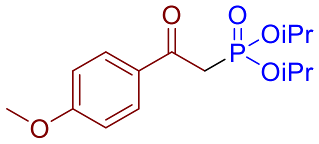

Diisopropyl (2-(4-methoxyphenyl)-2-oxoethyl)phosphonate (3m) was synthesized as yellow oil (56%, isolated by column chromatography using $CHCl_3$/MeOH = 60/1 as an eluent).[5] **^{1}H NMR** (300.23 MHz, $CDCl_3$): δ = 8.00 (d, *J* = 8.9 Hz, 2H), 6.93 (d, *J* = 8.9 Hz, 2H), 4.85 – 4.58 (m, 2H), 3.87 (s, 3H), 3.53 (d, *J* = 22.8 Hz, 2H), 1.28 (d, *J* = 2.7 Hz, 6H), 1.26 (d, *J* = 2.7 Hz, 6H). **^{13}C NMR** (75.50 MHz, $CDCl_3$): δ = 190.6 (d, *J* = 6.6 Hz), 164.0, 131.7, 130.0 (d, *J* = 1.4 Hz), 113.8, 71.5 (d, *J* = 6.8 Hz), 55.6, 39.7 (d, *J* = 130.1 Hz), 24.1 (d, *J* = 3.9 Hz), 23.9 (d, *J* = 5.2 Hz). **^{31}P** (121.54 MHz, $CDCl_3$): δ = 18.2.

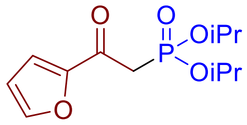

Diisopropyl (2-(furan-2-yl)-2-oxoethyl)phosphonate (3n) was synthesized as brown oil (19%, isolated by column chromatography using $CHCl_3$/MeOH = 60/1 as an eluent). **^{1}H NMR** (300.13 MHz, $CDCl_3$): δ = 7.61 (dd, *J* = 1.8, 0.8 Hz, 1H), 7.29 (dd, *J* = 3.6, 0.8 Hz, 1H), 6.55 (dd, *J* = 3.6, 1.7 Hz, 1H), 4.85 – 4.59 (m, 2H), 3.45 (d, *J* = 22.7 Hz, 2H), 1.29 (d, *J* = 1.6 Hz, 6H), 1.27 (d, *J* = 1.6 Hz, 6H). **^{13}C NMR** (75.47 MHz, $CDCl_3$): δ = 180.4 (d, *J* = 7.1 Hz), 152.5, 147.1, 119.0, 112.8, 71.7 (d, *J* = 6.6 Hz), 39.6 (d, *J* = 130.5 Hz), 24.1 (d, *J* = 3.9 Hz), 23.9 (d, *J* = 5.2 Hz). **^{31}P** (121.54 MHz, $CDCl_3$): δ = 17.3. **HR-MS** (ESI): *m/z* = 275.1043, calc. for $C_{12}H_{21}O_4P+H^+$: 275.1041. **FT-IR** (thin layer): ν_{max} = 2980, 2930, 1674, 1568, 1467, 1387, 1301, 1254, 1208, 1178, 1142, 1106, 987, 886, 770.

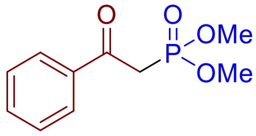

Dimethyl (2-oxo-2-phenylethyl)phosphonate (3o) was synthesized as yellow oil (28%, isolated by column chromatography using DCM/MeOH = 40/1 as an eluent).[6] **^{1}H NMR** (300.23 MHz, $CDCl_3$): δ = 8.06 – 7.91 (m, 2H), 7.64 – 7.53 (m, 1H), 7.52 – 7.39 (m, 2H), 3.76 (d, *J* = 11.2 Hz, 6H), 3.62 (d, *J* = 22.6 Hz, 2H). **^{13}C NMR** (75.50 MHz, $CDCl_3$): δ = 191.9 (d, *J* = 6.6 Hz), 136.5 (d, *J* = 2.5 Hz), 133.9, 129.1, 128.8, 53.2 (d, *J* = 6.5 Hz), 37.6 (d, *J* = 131.4 Hz). **^{31}P** (121.54 MHz, $CDCl_3$): δ = 22.8.

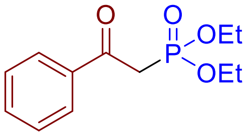

Diethyl (2-oxo-2-phenylethyl)phosphonate (3p) was synthesized as yellow oil (50%, isolated by column chromatography using $CHCl_3$/MeOH = 60/1 as an eluent).[5] **^{1}H NMR** (300.13 MHz, $CDCl_3$): δ = 8.15 – 7.81 (m, 2H), 7.70 – 7.52 (m, 1H), 7.51 – 7.31 (m, 2H), 4.31 – 3.93 (m, 4H), 3.62 (d, *J* = 22.7 Hz, 2H), 1.26 (t, *J* = 7.1 Hz, 6H). **^{13}C NMR** (75.50 MHz, $CDCl_3$): δ = 192.0 (d, *J* = 6.7 Hz), 136.6 (d, *J* = 1.8 Hz), 133.8, 129.1, 128.7, 62.8 (d, *J* = 6.5 Hz), 38.6 (d, *J* = 130.1 Hz), 16.3 (d, *J* = 6.3 Hz). **^{31}P** (121.54 MHz, $CDCl_3$): δ = 20.0.

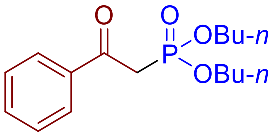

Dibutyl (2-oxo-2-phenylethyl)phosphonate (3q) was synthesized as yellow oil (63%, isolated by column chromatography using $CHCl_3$/MeOH = 60/1 as an eluent).[5] **^{1}H NMR** (300.13 MHz, $CDCl_3$): δ = 8.09–7.88 (m, 2H), 7.65–7.52 (m, 1H), 7.51–7.34 (m, 2H), 4.15–3.91 (m, 4H), 3.61 (d, *J* = 22.8 Hz, 2H), 1.69–1.45 (m, 4H), 1.42–1.15 (m, 4H), 0.86 (t, *J* = 7.4 Hz, 6H). **^{13}C NMR** (75.50 MHz, $CDCl_3$): δ = 192.0 (d, *J* = 6.7 Hz), 136.6 (d, *J* = 1.9 Hz), 133.7, 129.1, 128.6, 66.4 (d, *J* = 6.8 Hz), 38.4 (d, *J* = 129.5 Hz), 32.4 (d, *J* = 6.3 Hz), 18.7, 13.6. **^{31}P** (121.49 MHz, $CDCl_3$): δ = 20.8.

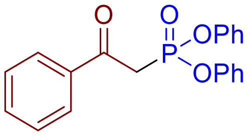

Diphenyl (2-oxo-2-phenylethyl)phosphonate (3r) was synthesized as yellow oil (26%, isolated by column chromatography using $CHCl_3$/MeOH = 60/1 as an eluent).[1] **^{1}H NMR** (300.13 MHz, $CDCl_3$): δ = 8.05–7.92 (m, 2H), 7.61–7.49 (m, 1H), 7.47–7.37 (m, 2H), 7.31 – 7.18 (m, 4H), 7.17 – 7.01 (m, 6H), 3.88 (d, *J* = 22.7 Hz, 2H). **^{13}C NMR** (75.50 MHz, $CDCl_3$): δ = 190.8 (d, *J* = 7.1 Hz), 150.1 (d, *J* = 8.8 Hz), 136.4 (d, *J* = 2.7 Hz), 134.0, 129.9 (d, *J* = 1.1 Hz), 129.1, 128.8, 125.5 (d, *J* = 1.4 Hz), 120.7 (d, *J* = 4.5 Hz), 37.9 (d, *J* = 133.4 Hz). **^{31}P** (121.54 MHz, $CDCl_3$): δ = 13.2.

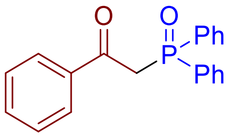

2-(diphenylphosphoryl)-1-phenylethan-1-one (3s) was synthesized as white solid (58%, isolated by column chromatography using DCM/EA = 5/1 with gradient elution to 1/1).[1] Mp = 134–135°C. **^{1}H NMR** (600.13 MHz, $CDCl_3$): δ = 8.08 – 7.92 (m, 2H), 7.90 – 7.71 (m, 4H), 7.65 – 7.33 (m, 9H), 4.17 (d, *J* = 15.1 Hz, 2H). **^{13}C NMR** (75.50 MHz, $CDCl_3$): δ =193.0 (d, *J* = 5.5 Hz), 137.1, 133.7, 132.28 (d, *J* = 2.8 Hz), 132.1 (d, *J* = 103.3 Hz), 131.24 (d, *J* = 9.8 Hz), 129.4, 128.8, 128.65 (d, *J* = 1.0 Hz), 43.46 (d, *J* = 57.9 Hz). **^{31}P** (242.93 MHz, $CDCl_3$): δ = 29.3.

Gram-scale synthesis of 3a (experimental data for Scheme 3).

Enol acetate **1a** (6 mmol, 973 mg), diisopropyl phosphite (**2a**, 12 mmol, 1.994 g), $CuSO_4·5H_2O$ (1.2 mmol, 300 mg), and MeCN (30 mL) were sequentially added to a round-bottom flask. The reaction mixture was stirred for 12 hours at 70 °C under air and then cooled to room temperature, and rotary-evaporated under reduced pressure. An additional evaporation step using a rotary

vane pump (0.5 mm Hg) at 80 °C was made for the evaporation of phosphite excess. β-ketophosphonate **3a** (1.31 g, 4.61 mmol, 77%) was isolated by column chromatography on silica gel on silica gel using $CHCl_3$/MeOH = 60/1 as an eluent.

Reaction of enolacetate 1a with diisopropyl phosphite (2a) at standard reaction conditions in the presence of radical scavengers (experimental data for Scheme 4, reaction 1).

Enol acetate (0.5 mmol, 81 mg), diisopropyl phosphite (**2a**, 1.0 mmol, 166 mg), $CuSO_4{\cdot}5H_2O$ (0.1 mmol, 25 mg), BHT (1.5 mmol, 330 mg) or TEMPO (1.5 mmol, 234 mg) and MeCN (5 mL) were sequentially added to a round-bottom flask. The reaction mixture was stirred for 3 hours at 70 °C under air and then cooled to room temperature, and rotary-evaporated under reduced pressure. The residue was analyzed using ^1H and ^{31}P NMR spectroscopy and HRMS.

Reaction of enolacetate 1a with diisopropyl phosphite (2a) at standard reaction conditions under O_2 or inert atmosphere (experimental data for Scheme 4, reaction 2b and 2c).

Enol acetate (0.5 mmol, 81 mg), diisopropyl phosphite (**2a**, 1.0 mmol, 166 mg), $CuSO_4{\cdot}5H_2O$ (0.1 mmol, 25 mg), and MeCN (5 mL) were sequentially added to a round-bottom flask. The flask was evacuated and backfilled with Ar or O_2 (this procedure was repeated three times). The reaction mixture was stirred for 3 hours at 70 °C, cooled to room temperature, and rotary-evaporated under reduced pressure. The residue was analyzed using ^1H and ^{31}P NMR spectroscopy.

Reaction of enolacetate 1a with diisopropyl phosphite (2a) with 10-fold excess of $CuSO_4{\cdot}5H_2O$ under inert atmosphere (experimental data for Scheme 4, reaction 2d).

Enol acetate (0.5 mmol, 81 mg), diisopropyl phosphite **2a** (1.0 mmol, 166 mg), $CuSO_4{\cdot}5H_2O$ (1 mmol, 250 mg), and MeCN (5 mL) were sequentially added to a round-bottom flask. The flask was evacuated and backfilled with Ar (this procedure was repeated three times). The reaction mixture was stirred for 3 hours at 70 °C, cooled to room temperature, and rotary-evaporated under reduced pressure. The residue was analyzed using ^1H and ^{31}P NMR spectroscopy.

Reaction of vinyl azide 4a or silyl enol ether 4b with diisopropyl phosphite (2a) at standard reaction conditions (experimental data for Scheme 4, reaction 3).

Vinyl azide **4a** (0.5 mmol, 73 mg) or silyl enol ether **4b** (0.5 mmol, 96 mg), diisopropyl phosphite (**2a**, 1.0 mmol, 166 mg), $CuSO_4{\cdot}5H_2O$ (1 mmol, 250 mg), and MeCN (5 mL) were sequentially added to a round-bottom flask. The flask was evacuated and backfilled with Ar (this procedure was repeated three times). The reaction mixture was stirred for 3 hours at 70 °C, cooled to room temperature, and rotary-evaporated under reduced pressure. The residue was analyzed using ^1H and ^{31}P NMR spectroscopy.

References:

(1) Chen, X.; Chen, X.; Li, X.; Qu, C.; Qu, L.; Bi, W.; Sun, K.; Zhao, Y. Acetonitrile-Dependent Oxyphosphorylation: A Mild One-Pot Synthesis of β-Ketophosphonates from Alkenyl Acids or Alkenes. *Tetrahedron* **2017**, *73* (17), 2439–2446. https://doi.org/10.1016/j.tet.2017.03.026.

(2) Lu, Y.; Li, Y.; Zhang, R.; Jin, K.; Duan, C. Highly Efficient Cu(I)-Catalyzed Trifluoromethylation of Aryl(Heteroaryl) Enol Acetates with CF3 Radicals Derived from CF3SO2Na and TBHP at Room Temperature. *J. Fluor. Chem.* **2014**, *161*, 128–133. https://doi.org/10.1016/j.jfluchem.2014.01.020.

(3) Paveliev, S. A.; Churakov, A. I.; Alimkhanova, L. S.; Segida, O. O.; Nikishin, G. I.; Terent'ev, A. O. Electrochemical Synthesis of *O* -Phthalimide Oximes from *α* -Azido Styrenes *via* Radical Sequence: Generation, Addition and Recombination of Imide- *N* -Oxyl and Iminyl Radicals with C−O/N−O Bonds Formation. *Adv. Synth. Catal.* **2020**, *362* (18), 3864–3871. https://doi.org/10.1002/adsc.202000618.

(4) Vil', V. A.; Gomes, G. D. P.; Ekimova, M. V.; Lyssenko, K. A.; Syroeshkin, M. A.; Nikishin, G. I.; Alabugin, I. V.; Terent'ev, A. O. Five Roads That Converge at the Cyclic Peroxy-Criegee Intermediates: BF_3 -Catalyzed Synthesis of β-Hydroperoxy-β-Peroxylactones. *J. Org. Chem.* **2018**, *83* (21), 13427–13445. https://doi.org/10.1021/acs.joc.8b02218.

(5) Wei, W.; Ji, J. Catalytic and Direct Oxyphosphorylation of Alkenes with Dioxygen and H-Phosphonates Leading to β-Ketophosphonates. *Angew. Chem.* **2011**, *123* (39), 9263–9265. https://doi.org/10.1002/ange.201100219.

(6) Zhou, M.; Chen, M.; Zhou, Y.; Yang, K.; Su, J.; Du, J.; Song, Q. β-Ketophosphonate Formation via Aerobic Oxyphosphorylation of Alkynes or Alkynyl Carboxylic Acids with H-Phosphonates. *Org. Lett.* **2015**, *17* (7), 1786–1789. https://doi.org/10.1021/acs.orglett.5b00574.

The ^{1}H, ^{13}C, and ^{31}P spectra of synthesized compounds

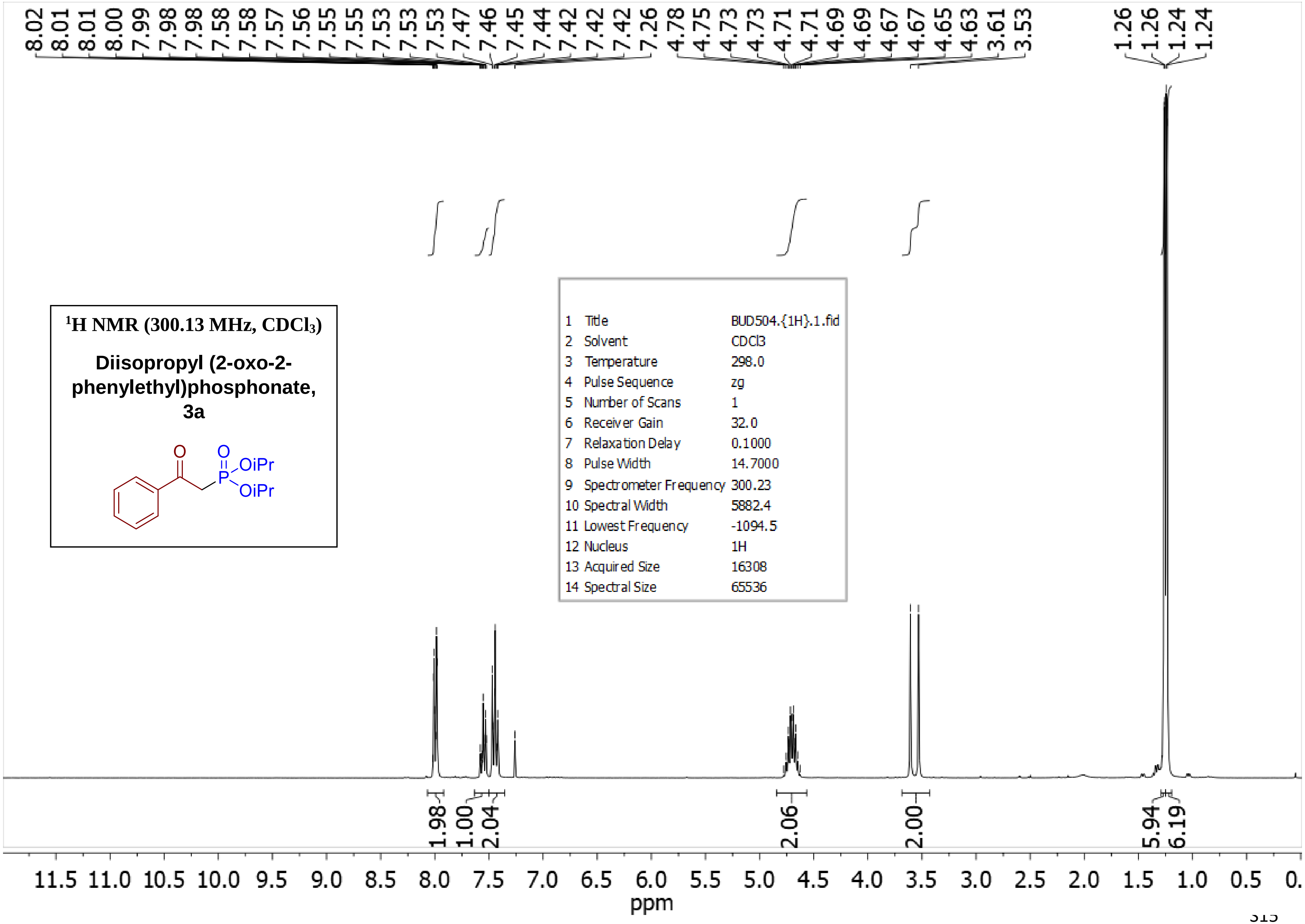
8.02 8.01 8.01 8.00 7.99 7.98 7.98 7.58 7.58 7.57 7.56 7.55 7.55 7.53 7.53 7.53 7.47 7.46 7.45 7.44 7.42 7.42 7.42 7.26 4.78 4.75 4.73 4.73 4.71 4.71 4.69 4.69 4.67 4.67 4.65 4.63 3.61 3.53
1.26 1.26 1.24 1.24
^{1}H NMR (300.13 MHz, CDCl$_3$)
Diisopropyl (2-oxo-2-phenylethyl)phosphonate, 3a
O
O
OiPr
P
OiPr
1 Title BUD504.{1H}.1.fid
2 Solvent CDCl3
3 Temperature 298.0
4 Pulse Sequence zg
5 Number of Scans 1
6 Receiver Gain 32.0
7 Relaxation Delay 0.1000
8 Pulse Width 14.7000
9 Spectrometer Frequency 300.23
10 Spectral Width 5882.4
11 Lowest Frequency -1094.5
12 Nucleus 1H
13 Acquired Size 16308
14 Spectral Size 65536
1.98 1.00 2.04 2.06 2.00 5.94 6.19
11.5 11.0 10.5 10.0 9.5 9.0 8.5 8.0 7.5 7.0 6.5 6.0 5.5 5.0 4.5 4.0 3.5 3.0 2.5 2.0 1.5 1.0 0.5 0.
ppm

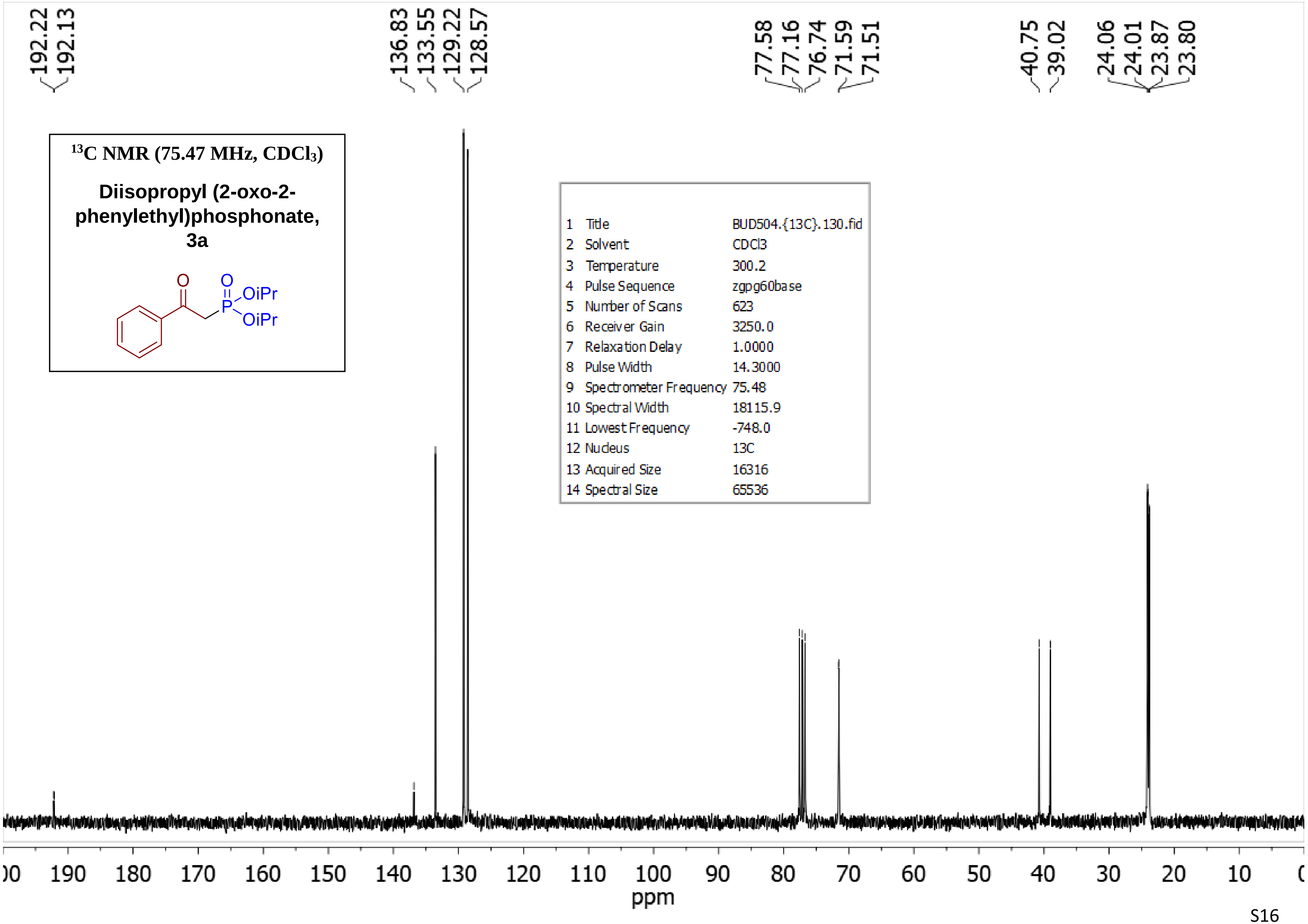
192.22
192.13
136.83
133.55
129.22
128.57
77.58
77.16
76.74
71.59
71.51
40.75
39.02
24.06
24.01
23.87
23.80
13C NMR (75.47 MHz, CDCl3)
Diisopropyl (2-oxo-2-phenylethyl)phosphonate, 3a
O
O
P
OiPr
OiPr
1 Title BUD504.{13C}.130.fid
2 Solvent CDCl3
3 Temperature 300.2
4 Pulse Sequence zgpg60base
5 Number of Scans 623
6 Receiver Gain 3250.0
7 Relaxation Delay 1.0000
8 Pulse Width 14.3000
9 Spectrometer Frequency 75.48
10 Spectral Width 18115.9
11 Lowest Frequency -748.0
12 Nucleus 13C
13 Acquired Size 16316
14 Spectral Size 65536
190
180
170
160
150
140
130
120
110
100
90
80
70
60
50
40
30
20
10
ppm

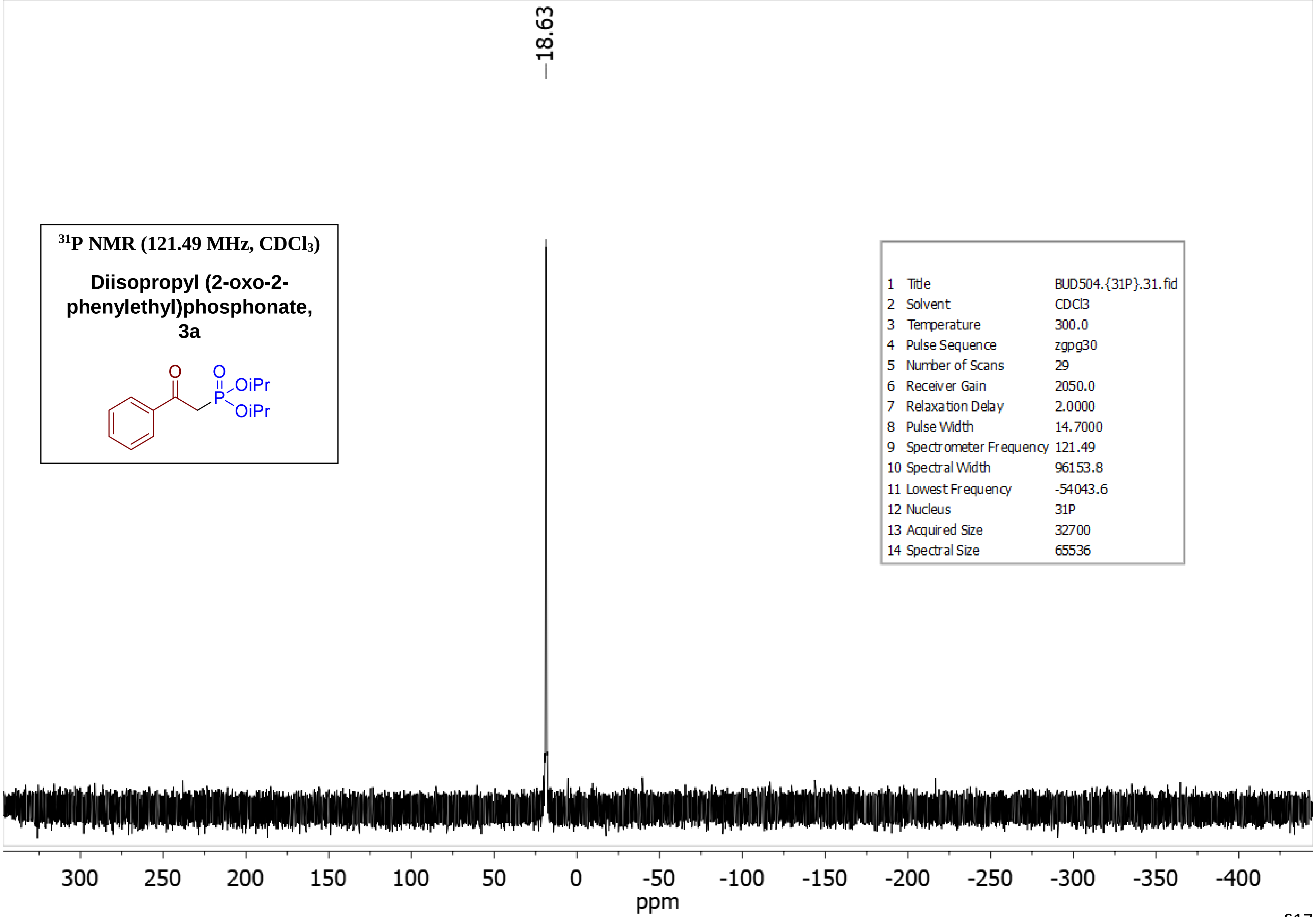

18.63
31P NMR (121.49 MHz, CDCl3)
Diisopropyl (2-oxo-2-phenylethyl)phosphonate, 3a
O
O
P
OiPr
OiPr
1 Title BUD504.{31P}.31.fid
2 Solvent CDCl3
3 Temperature 300.0
4 Pulse Sequence zgpg30
5 Number of Scans 29
6 Receiver Gain 2050.0
7 Relaxation Delay 2.0000
8 Pulse Width 14.7000
9 Spectrometer Frequency 121.49
10 Spectral Width 96153.8
11 Lowest Frequency -54043.6
12 Nucleus 31P
13 Acquired Size 32700
14 Spectral Size 65536
300 250 200 150 100 50 0 -50 -100 -150 -200 -250 -300 -350 -400
ppm

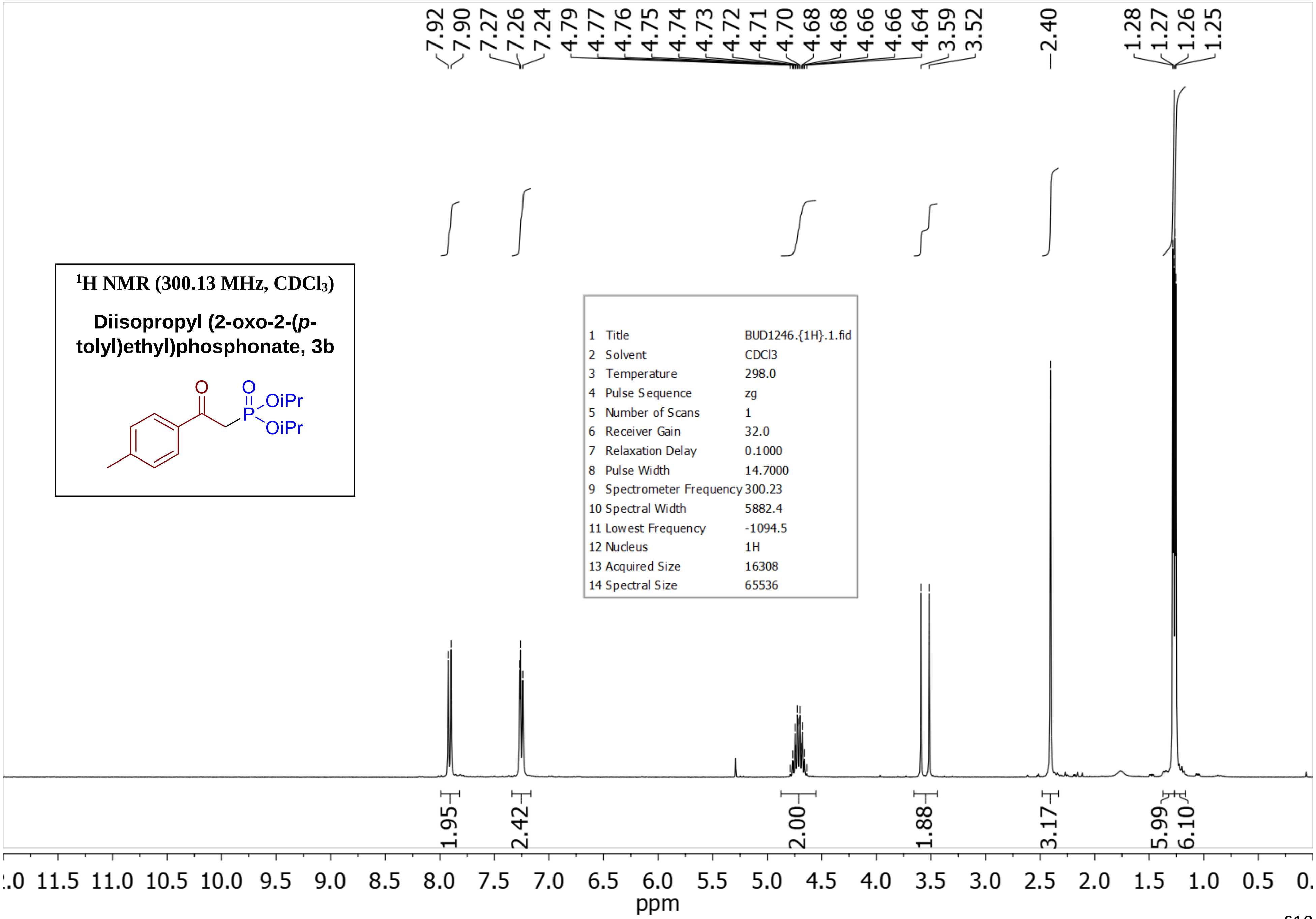

1H NMR (300.13 MHz, CDCl3)
Diisopropyl (2-oxo-2-(p-tolyl)ethyl)phosphonate, 3b
O
O
P
OiPr
OiPr
1 Title BUD1246.{1H}.1.fid
2 Solvent CDCl3
3 Temperature 298.0
4 Pulse Sequence zg
5 Number of Scans 1
6 Receiver Gain 32.0
7 Relaxation Delay 0.1000
8 Pulse Width 14.7000
9 Spectrometer Frequency 300.23
10 Spectral Width 5882.4
11 Lowest Frequency -1094.5
12 Nucleus 1H
13 Acquired Size 16308
14 Spectral Size 65536
7.92 7.90 7.27 7.26 7.24 4.79 4.77 4.76 4.75 4.74 4.74 4.73 4.72 4.71 4.70 4.68 4.68 4.66 4.66 4.64 3.59 3.52 2.40 1.28 1.27 1.26 1.25
1.95 2.42 2.00 1.88 3.17 5.99 6.10
11.5 11.0 10.5 10.0 9.5 9.0 8.5 8.0 7.5 7.0 6.5 6.0 5.5 5.0 4.5 4.0 3.5 3.0 2.5 2.0 1.5 1.0 0.5
ppm

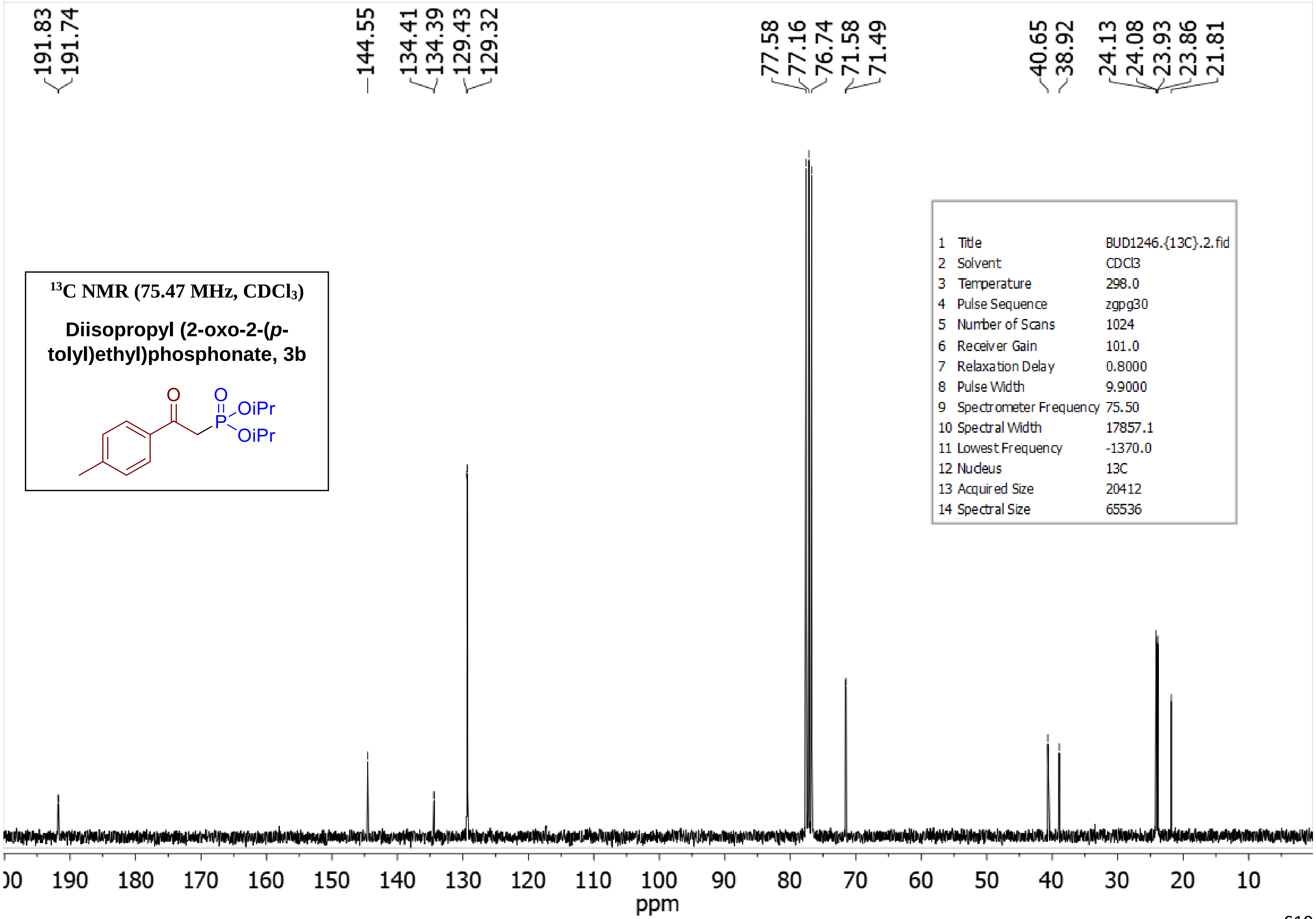
191.83
191.74
144.55
134.41
134.39
129.43
129.32
77.58
77.16
76.74
71.58
71.49
40.65
38.92
24.13
24.08
23.93
23.86
21.81
13C NMR (75.47 MHz, CDCl3)
Diisopropyl (2-oxo-2-(p-tolyl)ethyl)phosphonate, 3b
O
O
P
OiPr
OiPr
1 Title BUD1246.{13C}.2.fid
2 Solvent CDCl3
3 Temperature 298.0
4 Pulse Sequence zgpg30
5 Number of Scans 1024
6 Receiver Gain 101.0
7 Relaxation Delay 0.8000
8 Pulse Width 9.9000
9 Spectrometer Frequency 75.50
10 Spectral Width 17857.1
11 Lowest Frequency -1370.0
12 Nucleus 13C
13 Acquired Size 20412
14 Spectral Size 65536
190
180
170
160
150
140
130
120
110
100
90
80
70
60
50
40
30
20
10
ppm

^{31}P NMR (121.54 MHz, $CDCl_3$)

Diisopropyl (2-oxo-2-(*p*-tolyl)ethyl)phosphonate, 3b

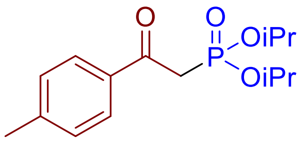

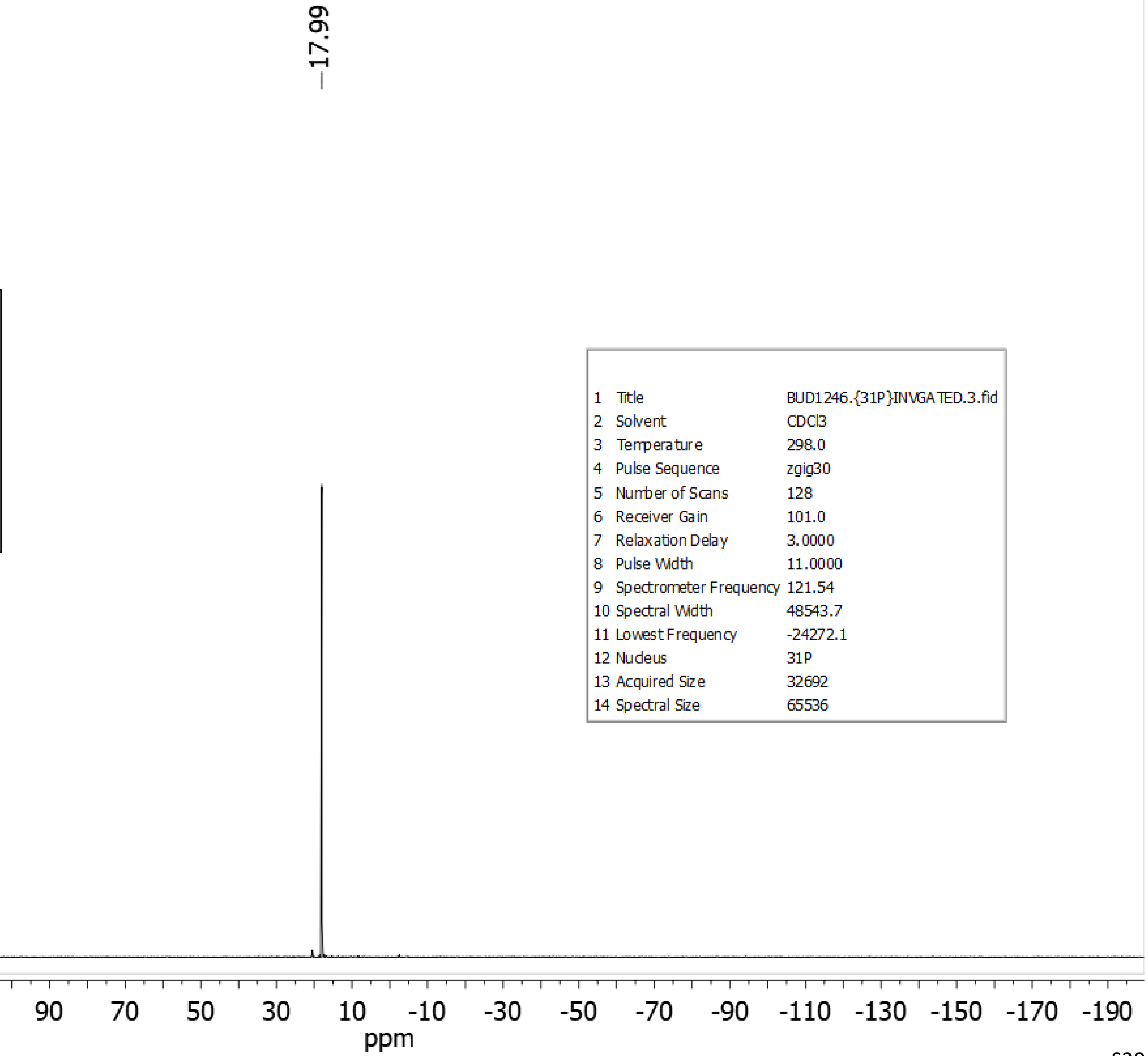

1	Title	BUD1246.{31P}INVGATED.3.fid
2	Solvent	CDCl3
3	Temperature	298.0
4	Pulse Sequence	zgig30
5	Number of Scans	128
6	Receiver Gain	101.0
7	Relaxation Delay	3.0000
8	Pulse Width	11.0000
9	Spectrometer Frequency	121.54
10	Spectral Width	48543.7
11	Lowest Frequency	-24272.1
12	Nucleus	31P
13	Acquired Size	32692
14	Spectral Size	65536

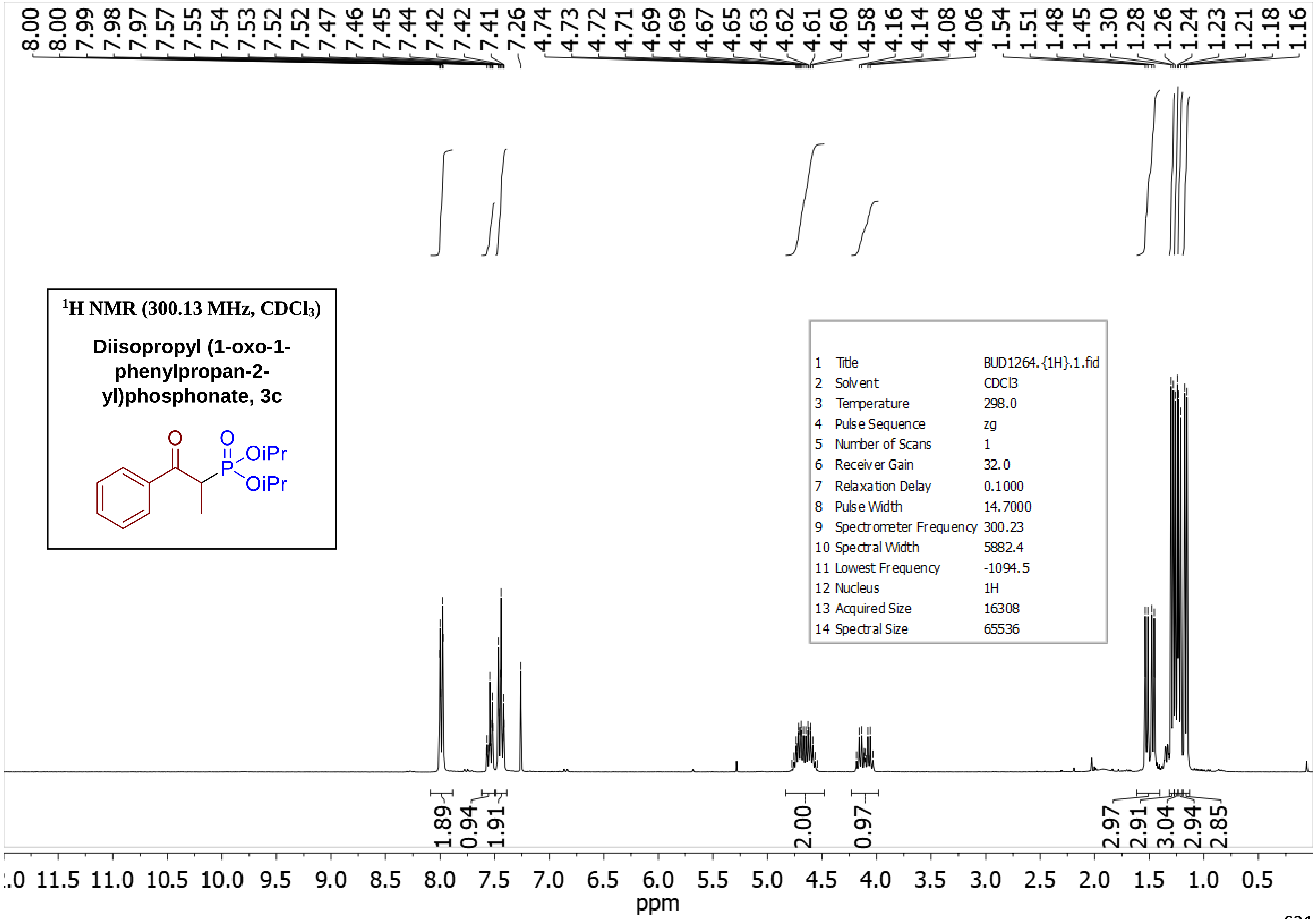
8.00 8.00 7.99 7.98 7.97 7.57 7.55 7.54 7.53 7.52 7.52 7.47 7.46 7.45 7.44 7.42 7.42 7.41 7.26 4.74 4.73 4.72 4.71 4.69 4.69 4.67 4.65 4.63 4.62 4.61 4.60 4.58 4.16 4.14 4.08 4.06 1.54 1.51 1.48 1.45 1.30 1.28 1.26 1.24 1.23 1.21 1.18 1.16
¹H NMR (300.13 MHz, CDCl3)
Diisopropyl (1-oxo-1-phenylpropan-2-yl)phosphonate, 3c
O
O
P
OiPr
OiPr
1 Title BUD1264.{1H}.1.fid
2 Solvent CDCl3
3 Temperature 298.0
4 Pulse Sequence zg
5 Number of Scans 1
6 Receiver Gain 32.0
7 Relaxation Delay 0.1000
8 Pulse Width 14.7000
9 Spectrometer Frequency 300.23
10 Spectral Width 5882.4
11 Lowest Frequency -1094.5
12 Nucleus 1H
13 Acquired Size 16308
14 Spectral Size 65536
1.89 0.94 1.91 2.00 0.97 2.97 2.91 3.04 2.94 2.85
11.5 11.0 10.5 10.0 9.5 9.0 8.5 8.0 7.5 7.0 6.5 6.0 5.5 5.0 4.5 4.0 3.5 3.0 2.5 2.0 1.5 1.0 0.5
ppm

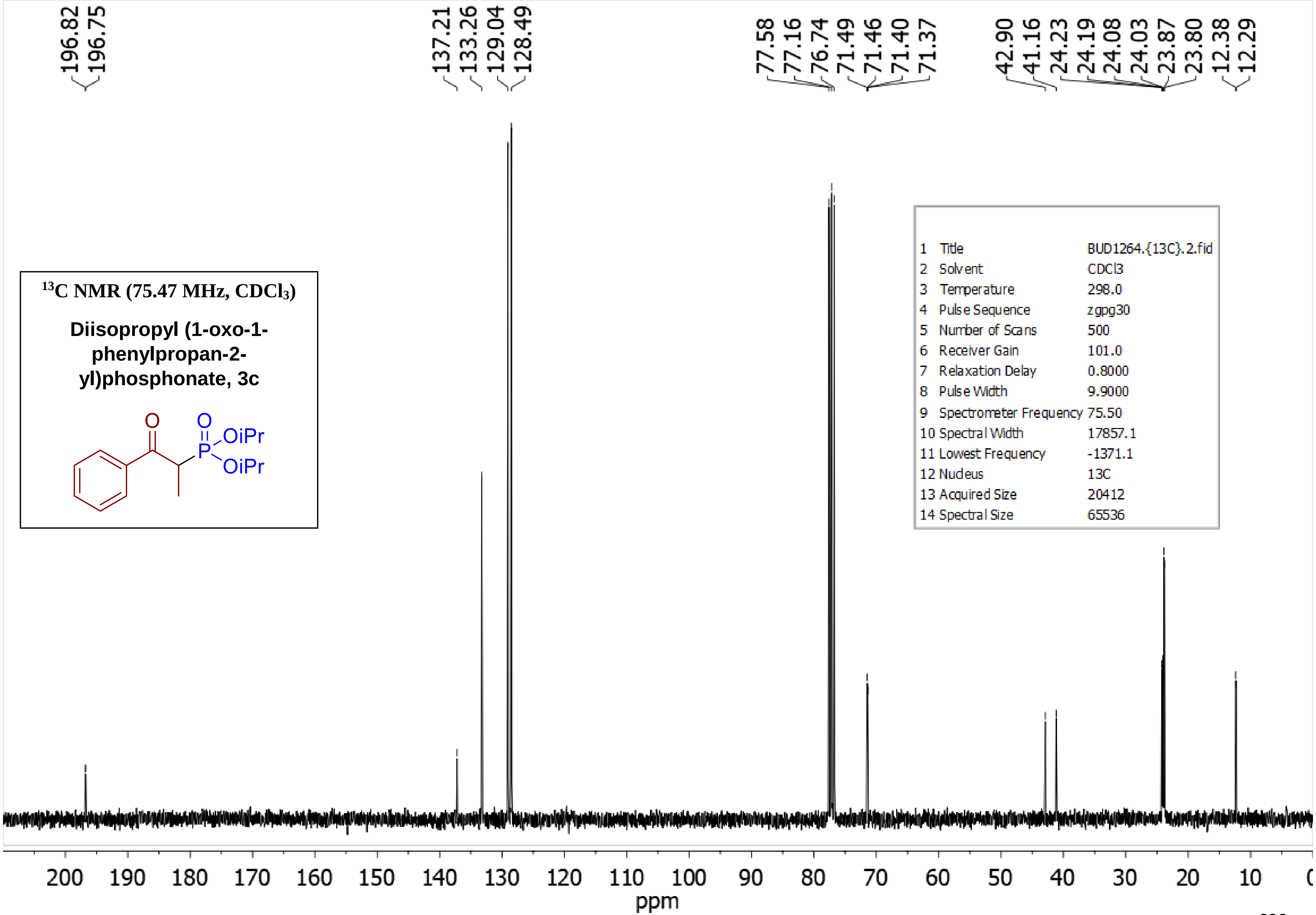
196.82
196.75
137.21
133.26
129.04
128.49
77.58
77.16
76.74
71.49
71.46
71.40
71.37
42.90
41.16
24.23
24.19
24.08
24.03
23.87
23.80
12.38
12.29
13C NMR (75.47 MHz, CDCl3)
Diisopropyl (1-oxo-1-phenylpropan-2-yl)phosphonate, 3c
O
O
P
OiPr
OiPr
1 Title BUD1264.{13C}.2.fid
2 Solvent CDCl3
3 Temperature 298.0
4 Pulse Sequence zgpg30
5 Number of Scans 500
6 Receiver Gain 101.0
7 Relaxation Delay 0.8000
8 Pulse Width 9.9000
9 Spectrometer Frequency 75.50
10 Spectral Width 17857.1
11 Lowest Frequency -1371.1
12 Nucleus 13C
13 Acquired Size 20412
14 Spectral Size 65536
200 190 180 170 160 150 140 130 120 110 100 90 80 70 60 50 40 30 20 10
ppm

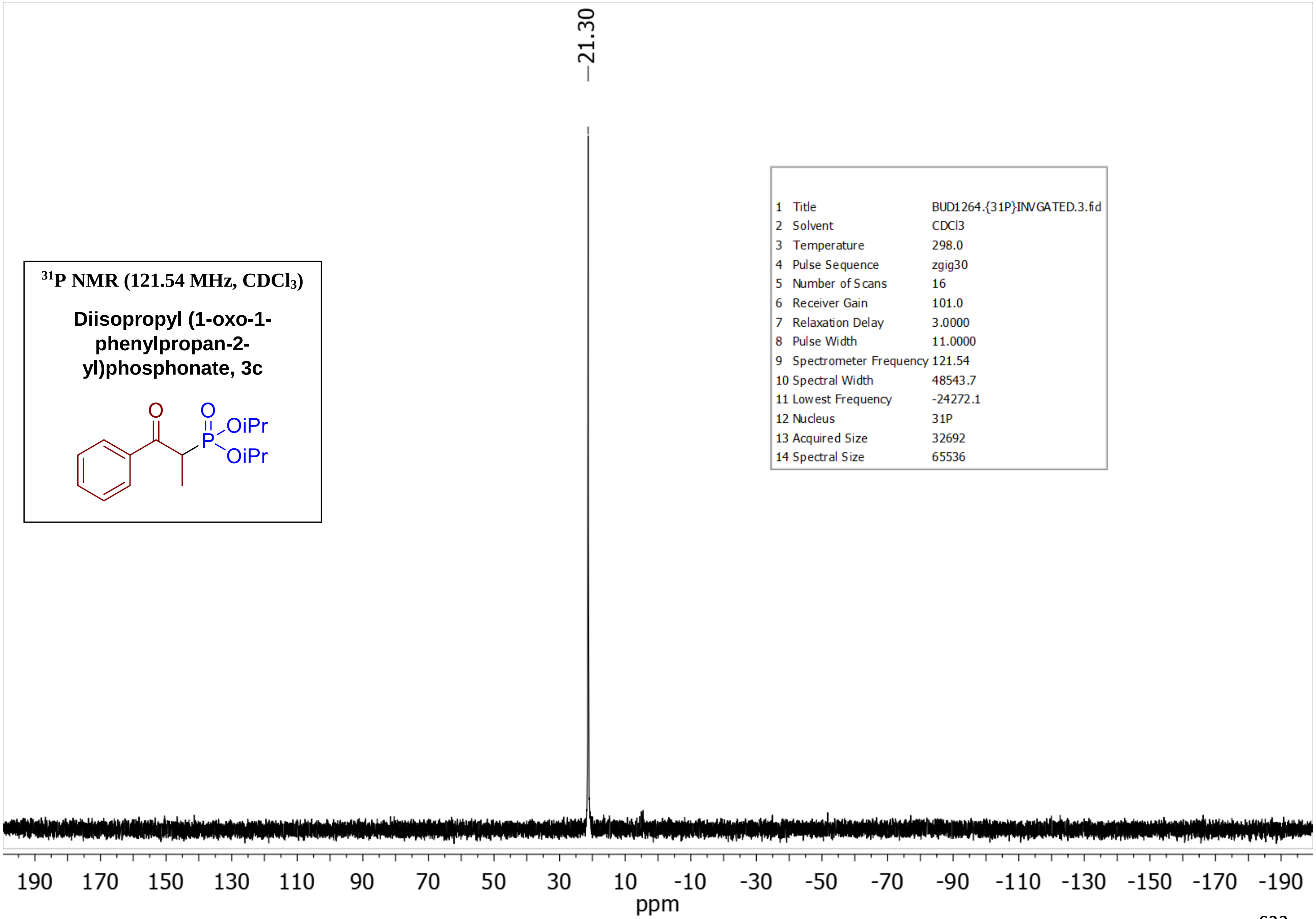

^{31}P NMR (121.54 MHz, CDCl$_3$)
Diisopropyl (1-oxo-1-phenylpropan-2-yl)phosphonate, 3c
O
O
OiPr
P
OiPr
21.30
1 Title BUD1264.{31P}INVGATED.3.fid
2 Solvent CDCl3
3 Temperature 298.0
4 Pulse Sequence zgig30
5 Number of Scans 16
6 Receiver Gain 101.0
7 Relaxation Delay 3.0000
8 Pulse Width 11.0000
9 Spectrometer Frequency 121.54
10 Spectral Width 48543.7
11 Lowest Frequency -24272.1
12 Nucleus 31P
13 Acquired Size 32692
14 Spectral Size 65536
190 170 150 130 110 90 70 50 30 10 -10 -30 -50 -70 -90 -110 -130 -150 -170 -190
ppm

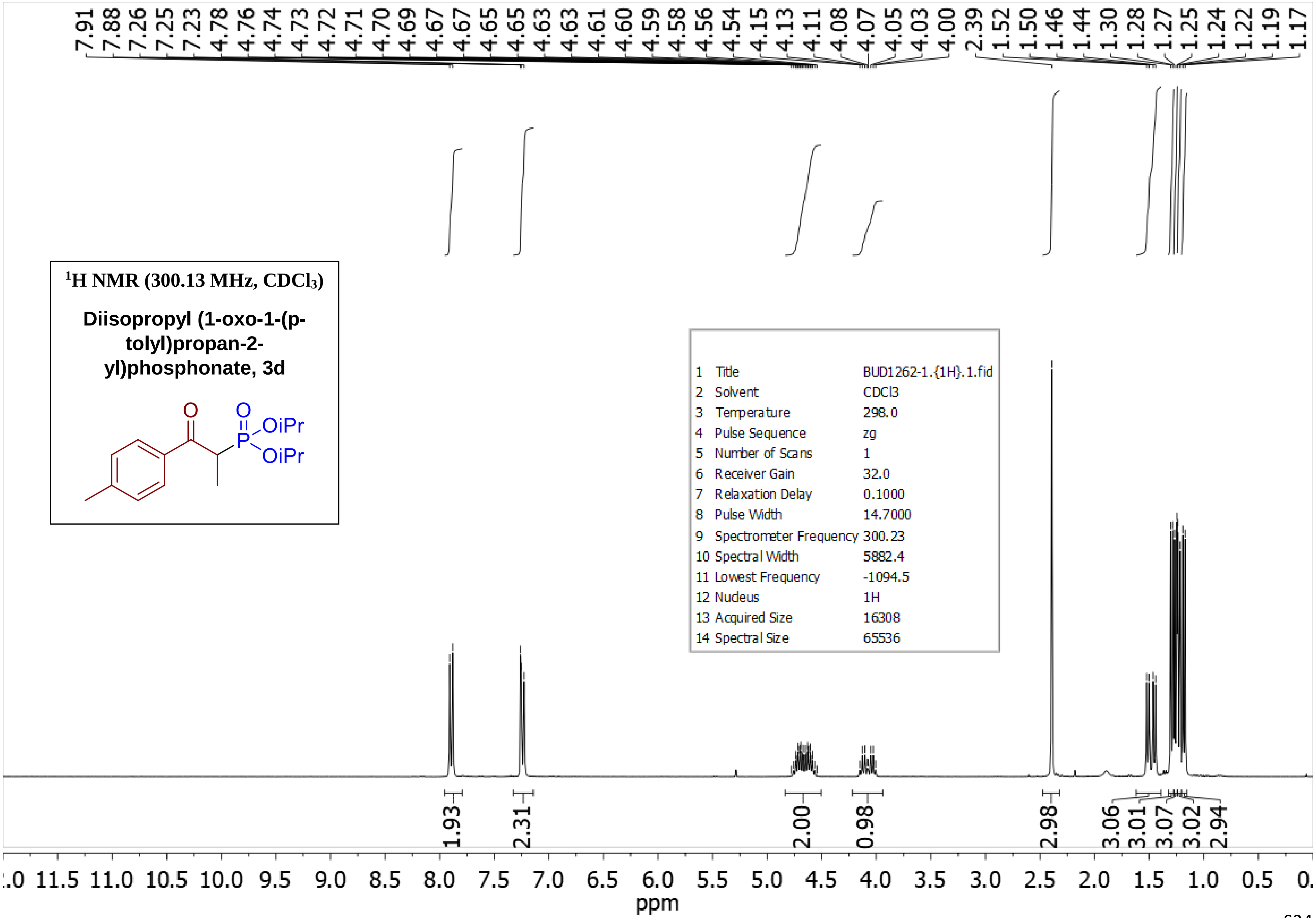

^{1}H NMR (300.13 MHz, CDCl$_3$)
Diisopropyl (1-oxo-1-(p-tolyl)propan-2-yl)phosphonate, 3d
O
O
P
OiPr
OiPr
7.91 7.88 7.26 7.25 7.23 4.78 4.76 4.74 4.73 4.72 4.71 4.70 4.69 4.67 4.67 4.65 4.65 4.63 4.63 4.61 4.60 4.59 4.58 4.56 4.54 4.15 4.13 4.11 4.08 4.07 4.05 4.03 4.00 2.39 1.52 1.50 1.46 1.44 1.30 1.28 1.27 1.25 1.24 1.22 1.19 1.17
1 Title BUD1262-1.{1H}.1.fid
2 Solvent CDCl3
3 Temperature 298.0
4 Pulse Sequence zg
5 Number of Scans 1
6 Receiver Gain 32.0
7 Relaxation Delay 0.1000
8 Pulse Width 14.7000
9 Spectrometer Frequency 300.23
10 Spectral Width 5882.4
11 Lowest Frequency -1094.5
12 Nucleus 1H
13 Acquired Size 16308
14 Spectral Size 65536
1.93 2.31 2.00 0.98 2.98 3.06 3.01 3.07 3.02 2.94
.0 11.5 11.0 10.5 10.0 9.5 9.0 8.5 8.0 7.5 7.0 6.5 6.0 5.5 5.0 4.5 4.0 3.5 3.0 2.5 2.0 1.5 1.0 0.5 0.
ppm

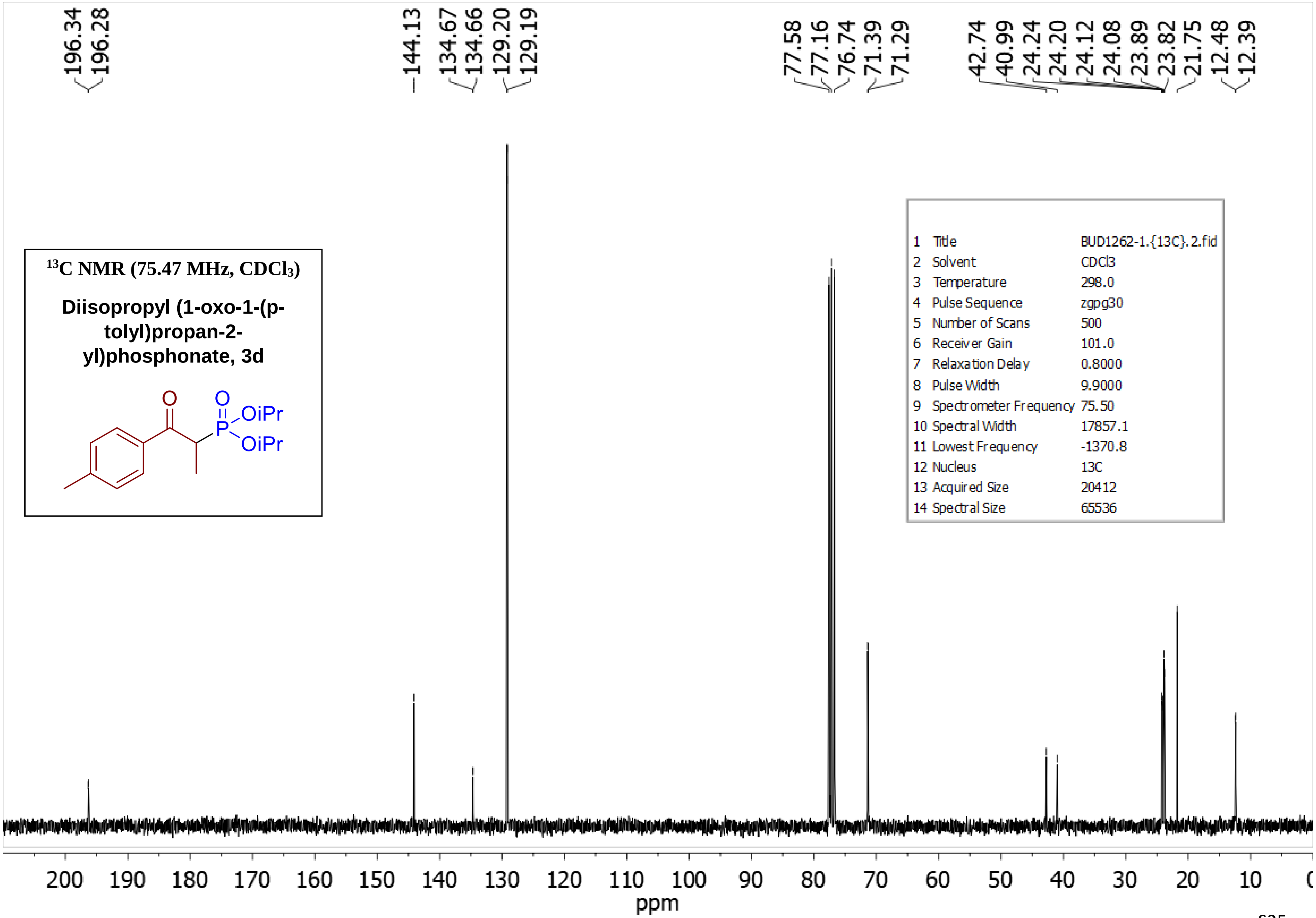
196.34
196.28
144.13
134.67
134.66
129.20
129.19
77.58
77.16
76.74
71.39
71.29
42.74
40.99
24.24
24.20
24.12
24.08
23.89
23.82
21.75
12.48
12.39
13C NMR (75.47 MHz, CDCl3)
Diisopropyl (1-oxo-1-(p-tolyl)propan-2-yl)phosphonate, 3d
O
O
OiPr
P
OiPr
1 Title BUD1262-1.{13C}.2.fid
2 Solvent CDCl3
3 Temperature 298.0
4 Pulse Sequence zgpg30
5 Number of Scans 500
6 Receiver Gain 101.0
7 Relaxation Delay 0.8000
8 Pulse Width 9.9000
9 Spectrometer Frequency 75.50
10 Spectral Width 17857.1
11 Lowest Frequency -1370.8
12 Nucleus 13C
13 Acquired Size 20412
14 Spectral Size 65536
200 190 180 170 160 150 140 130 120 110 100 90 80 70 60 50 40 30 20 10
ppm

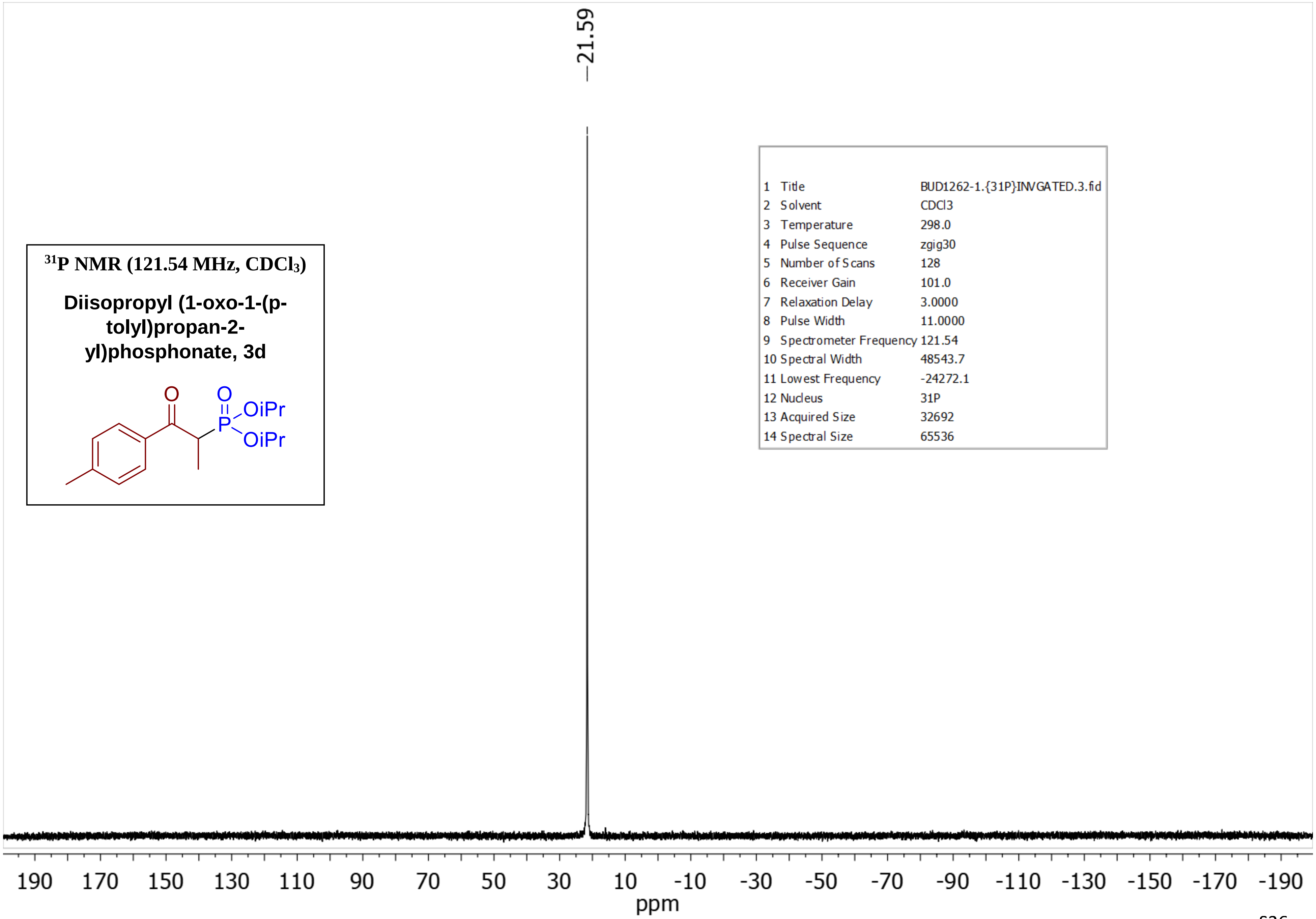
21.59
^{31}P NMR (121.54 MHz, CDCl$_3$)
Diisopropyl (1-oxo-1-(p-tolyl)propan-2-yl)phosphonate, 3d
O
O
P
OiPr
OiPr
1 Title BUD1262-1.{31P}INVGATED.3.fid
2 Solvent CDCl3
3 Temperature 298.0
4 Pulse Sequence zgig30
5 Number of Scans 128
6 Receiver Gain 101.0
7 Relaxation Delay 3.0000
8 Pulse Width 11.0000
9 Spectrometer Frequency 121.54
10 Spectral Width 48543.7
11 Lowest Frequency -24272.1
12 Nucleus 31P
13 Acquired Size 32692
14 Spectral Size 65536
190 170 150 130 110 90 70 50 30 10 -10 -30 -50 -70 -90 -110 -130 -150 -170 -190
ppm

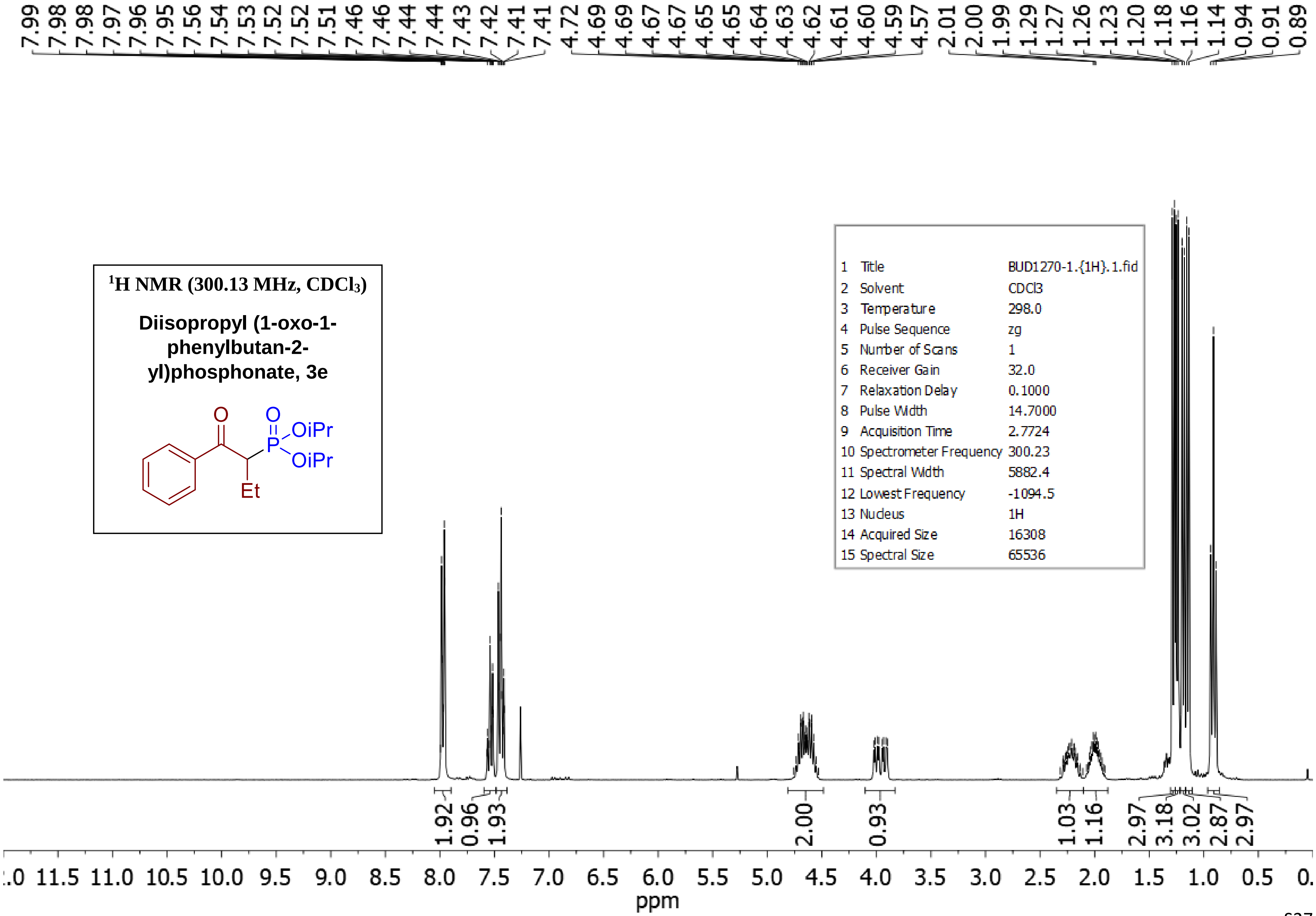

7.99 7.98 7.98 7.97 7.96 7.95 7.56 7.54 7.53 7.52 7.52 7.51 7.46 7.46 7.44 7.44 7.43 7.42 7.41 7.41 4.72 4.69 4.69 4.67 4.67 4.65 4.65 4.64 4.63 4.62 4.61 4.60 4.59 4.57 2.01 2.00 1.99 1.29 1.27 1.26 1.23 1.20 1.18 1.16 1.14 0.94 0.91 0.89
¹H NMR (300.13 MHz, CDCl3)
Diisopropyl (1-oxo-1-phenylbutan-2-yl)phosphonate, 3e
O
O
P
OiPr
OiPr
Et
1 Title BUD1270-1.{1H}.1.fid
2 Solvent CDCl3
3 Temperature 298.0
4 Pulse Sequence zg
5 Number of Scans 1
6 Receiver Gain 32.0
7 Relaxation Delay 0.1000
8 Pulse Width 14.7000
9 Acquisition Time 2.7724
10 Spectrometer Frequency 300.23
11 Spectral Width 5882.4
12 Lowest Frequency -1094.5
13 Nucleus 1H
14 Acquired Size 16308
15 Spectral Size 65536
1.92 0.96 1.93 2.00 0.93 1.03 1.16 2.97 3.18 3.02 2.87 2.97
11.5 11.0 10.5 10.0 9.5 9.0 8.5 8.0 7.5 7.0 6.5 6.0 5.5 5.0 4.5 4.0 3.5 3.0 2.5 2.0 1.5 1.0 0.5
ppm

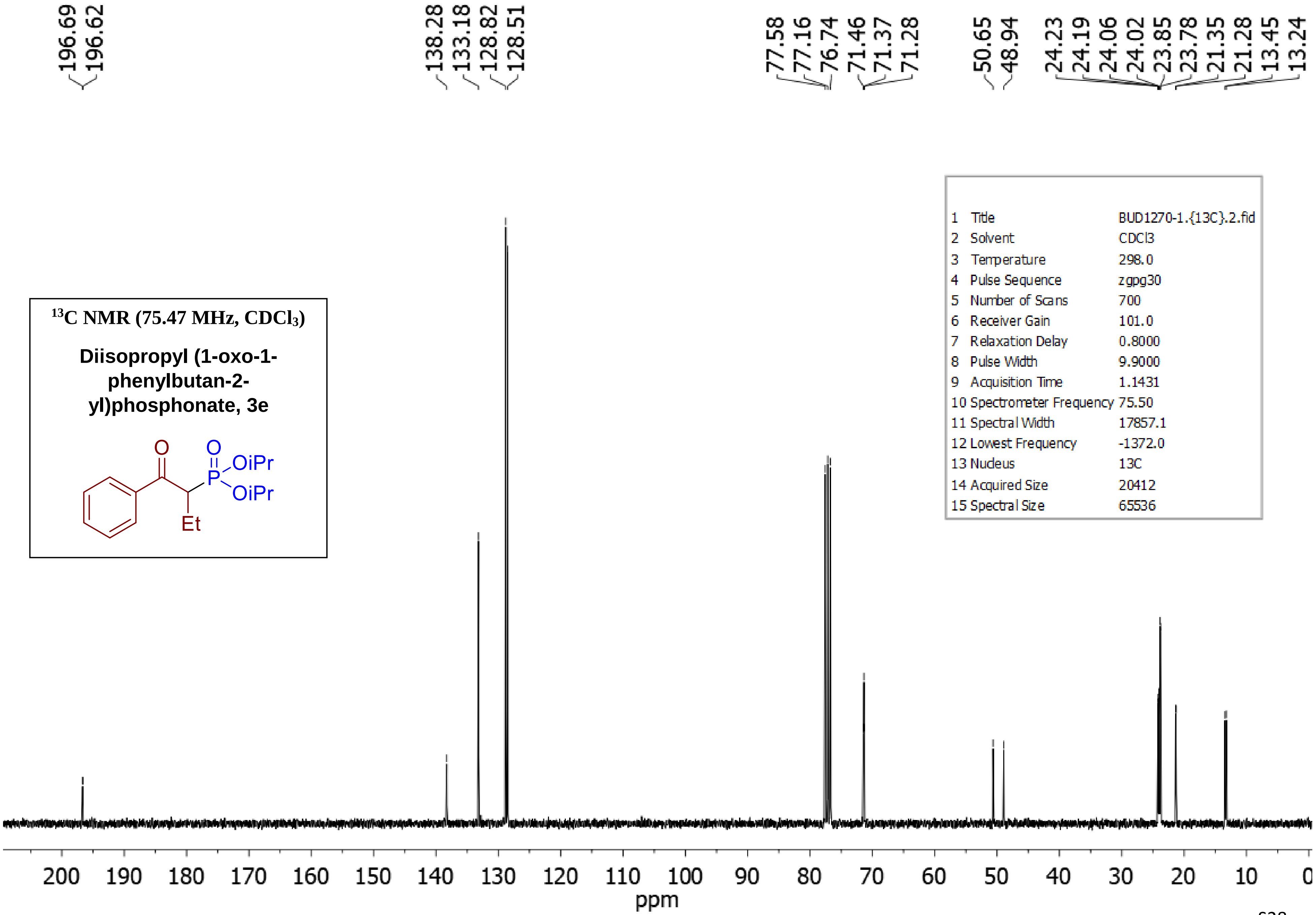
196.69
196.62
138.28
133.18
128.82
128.51
77.58
77.16
76.74
71.46
71.37
71.28
50.65
48.94
24.23
24.19
24.06
24.02
23.85
23.78
21.35
21.28
13.45
13.24
13C NMR (75.47 MHz, CDCl3)
Diisopropyl (1-oxo-1-phenylbutan-2-yl)phosphonate, 3e
O
O
OiPr
P
OiPr
Et
1 Title BUD1270-1.{13C}.2.fid
2 Solvent CDCl3
3 Temperature 298.0
4 Pulse Sequence zgpg30
5 Number of Scans 700
6 Receiver Gain 101.0
7 Relaxation Delay 0.8000
8 Pulse Width 9.9000
9 Acquisition Time 1.1431
10 Spectrometer Frequency 75.50
11 Spectral Width 17857.1
12 Lowest Frequency -1372.0
13 Nucleus 13C
14 Acquired Size 20412
15 Spectral Size 65536
200
190
180
170
160
150
140
130
120
110
100
90
80
70
60
50
40
30
20
10
0
ppm

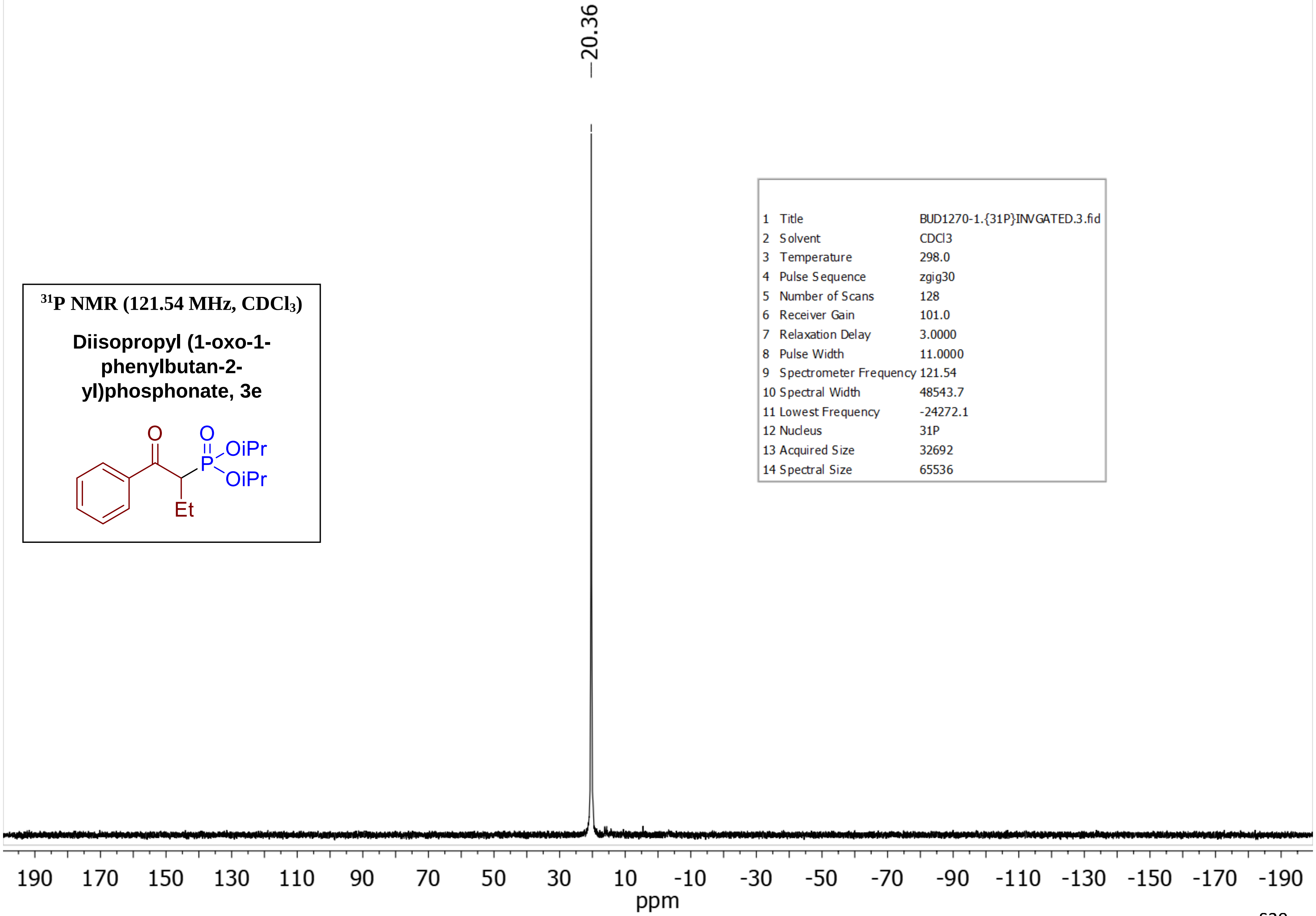
20.36
^{31}P NMR (121.54 MHz, $CDCl_3$)
Diisopropyl (1-oxo-1-phenylbutan-2-yl)phosphonate, 3e
O
O
OiPr
P
OiPr
Et
1 Title BUD1270-1.{31P}INVGATED.3.fid
2 Solvent CDCl3
3 Temperature 298.0
4 Pulse Sequence zgig30
5 Number of Scans 128
6 Receiver Gain 101.0
7 Relaxation Delay 3.0000
8 Pulse Width 11.0000
9 Spectrometer Frequency 121.54
10 Spectral Width 48543.7
11 Lowest Frequency -24272.1
12 Nucleus 31P
13 Acquired Size 32692
14 Spectral Size 65536
190 170 150 130 110 90 70 50 30 10 -10 -30 -50 -70 -90 -110 -130 -150 -170 -190
ppm

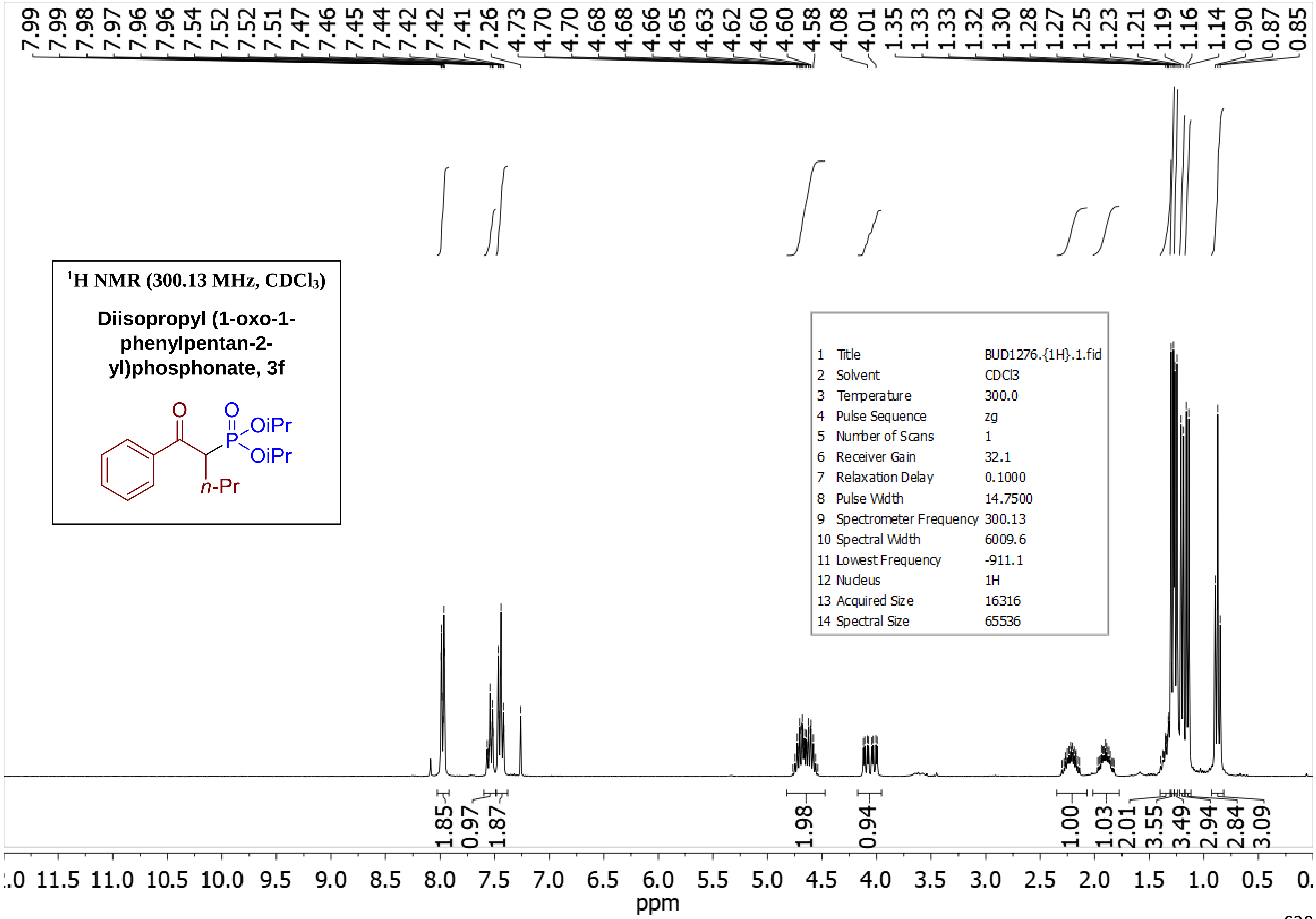

¹H NMR (300.13 MHz, CDCl₃)
Diisopropyl (1-oxo-1-phenylpentan-2-yl)phosphonate, 3f
O
O
P
OiPr
OiPr
n-Pr
7.99 7.99 7.98 7.97 7.96 7.96 7.54 7.52 7.52 7.51 7.47 7.46 7.45 7.44 7.42 7.42 7.41 7.26 4.73 4.70 4.70 4.68 4.68 4.66 4.65 4.63 4.62 4.60 4.60 4.58 4.08 4.01 1.35 1.33 1.33 1.32 1.30 1.28 1.27 1.25 1.23 1.21 1.19 1.16 1.14 0.90 0.87 0.85
1 Title BUD1276.{1H}.1.fid
2 Solvent CDCl3
3 Temperature 300.0
4 Pulse Sequence zg
5 Number of Scans 1
6 Receiver Gain 32.1
7 Relaxation Delay 0.1000
8 Pulse Width 14.7500
9 Spectrometer Frequency 300.13
10 Spectral Width 6009.6
11 Lowest Frequency -911.1
12 Nucleus 1H
13 Acquired Size 16316
14 Spectral Size 65536
1.85 0.97 1.87 1.98 0.94 1.00 1.03 2.01 3.55 3.49 2.94 2.84 3.09
11.5 11.0 10.5 10.0 9.5 9.0 8.5 8.0 7.5 7.0 6.5 6.0 5.5 5.0 4.5 4.0 3.5 3.0 2.5 2.0 1.5 1.0 0.5
ppm

^{13}C NMR (75.47 MHz, $CDCl_3$)

Diisopropyl (1-oxo-1-phenylpentan-2-yl)phosphonate, 3f

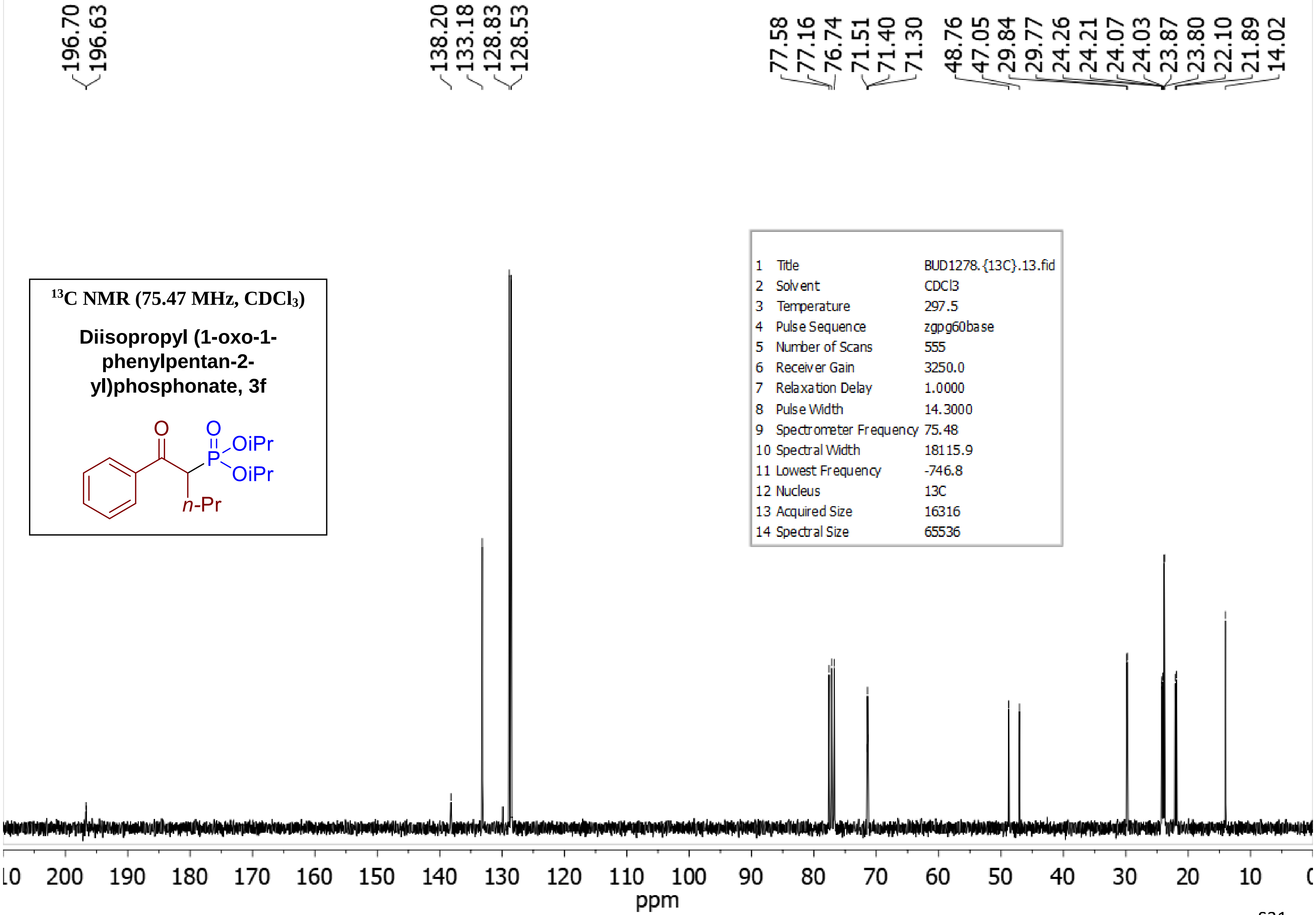

1	Title	BUD1278.{13C}.13.fid
2	Solvent	CDCl3
3	Temperature	297.5
4	Pulse Sequence	zgpg60base
5	Number of Scans	555
6	Receiver Gain	3250.0
7	Relaxation Delay	1.0000
8	Pulse Width	14.3000
9	Spectrometer Frequency	75.48
10	Spectral Width	18115.9
11	Lowest Frequency	-746.8
12	Nucleus	13C
13	Acquired Size	16316
14	Spectral Size	65536

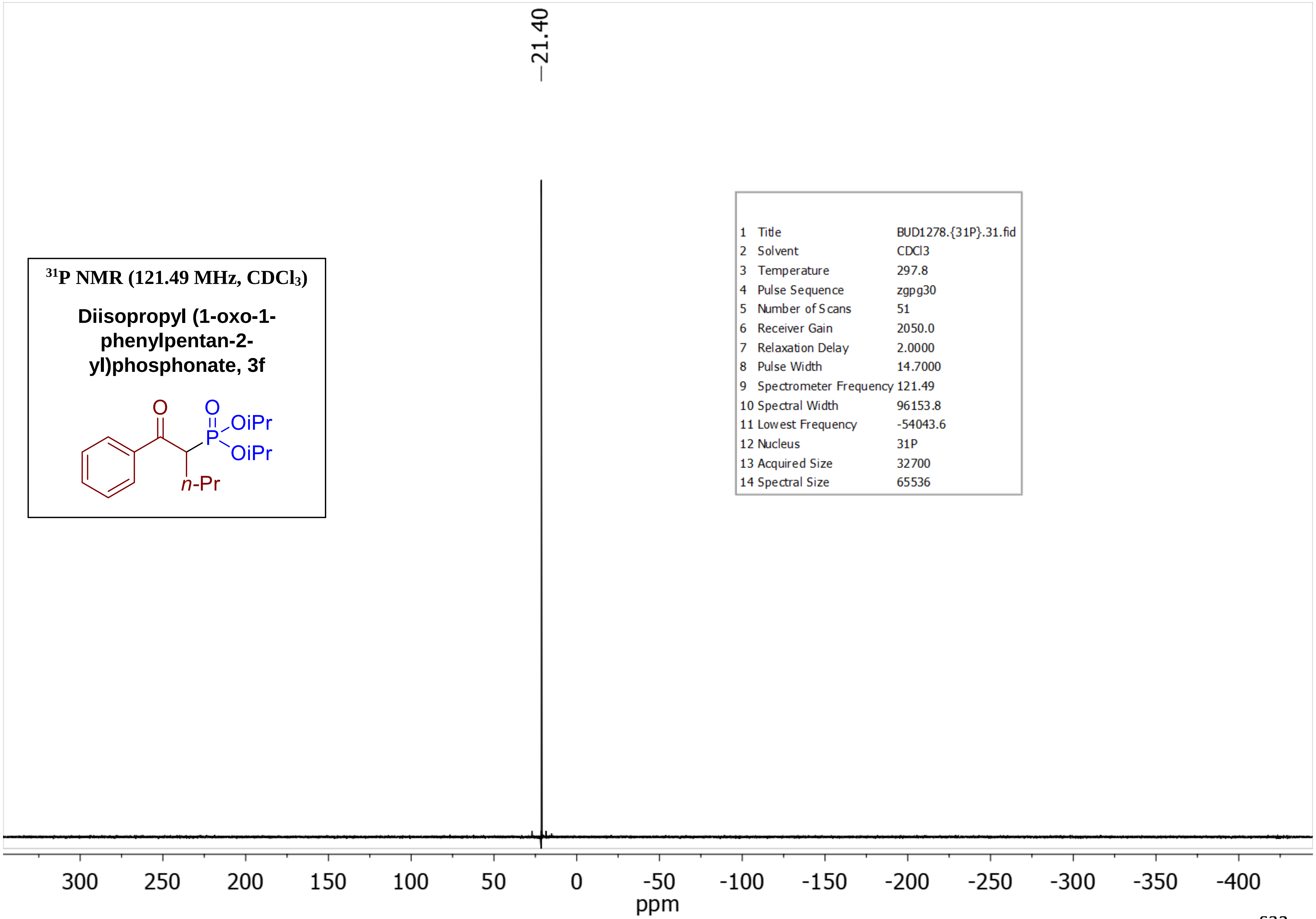

^{31}P NMR (121.49 MHz, $CDCl_3$)

Diisopropyl (1-oxo-1-phenylpentan-2-yl)phosphonate, 3f

1	Title	BUD1278.{31P}.31.fid
2	Solvent	CDCl3
3	Temperature	297.8
4	Pulse Sequence	zgpg30
5	Number of Scans	51
6	Receiver Gain	2050.0
7	Relaxation Delay	2.0000
8	Pulse Width	14.7000
9	Spectrometer Frequency	121.49
10	Spectral Width	96153.8
11	Lowest Frequency	-54043.6
12	Nucleus	31P
13	Acquired Size	32700
14	Spectral Size	65536

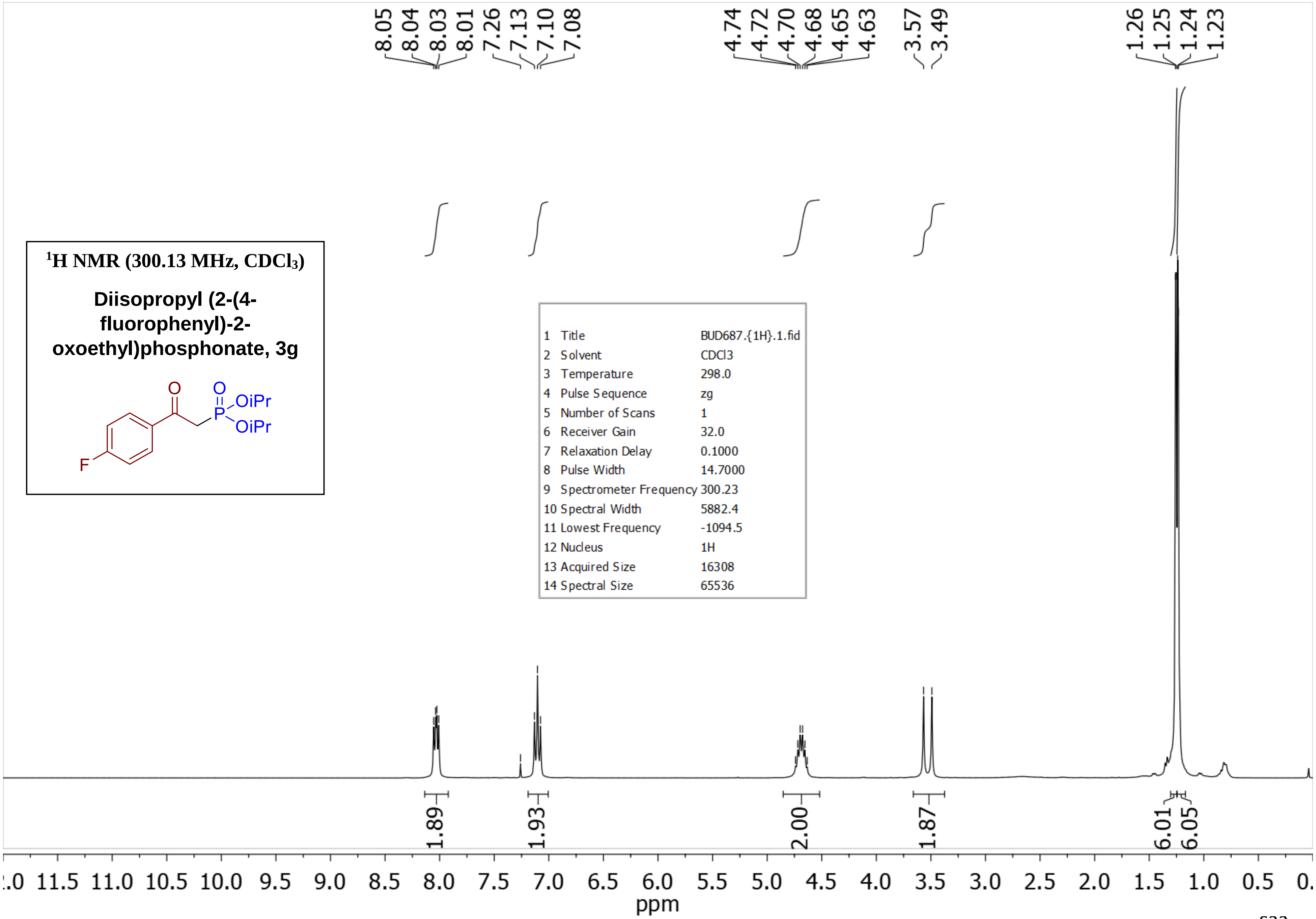

1H NMR (300.13 MHz, CDCl3)
Diisopropyl (2-(4-fluorophenyl)-2-oxoethyl)phosphonate, 3g
O
O
OiPr
OiPr
P
F
1 Title BUD687.{1H}.1.fid
2 Solvent CDCl3
3 Temperature 298.0
4 Pulse Sequence zg
5 Number of Scans 1
6 Receiver Gain 32.0
7 Relaxation Delay 0.1000
8 Pulse Width 14.7000
9 Spectrometer Frequency 300.23
10 Spectral Width 5882.4
11 Lowest Frequency -1094.5
12 Nucleus 1H
13 Acquired Size 16308
14 Spectral Size 65536
8.05 8.04 8.03 8.01 7.26 7.13 7.10 7.08
4.74 4.72 4.70 4.68 4.65 4.63
3.57 3.49
1.26 1.25 1.24 1.23
1.89 1.93 2.00 1.87 6.01 6.05
ppm

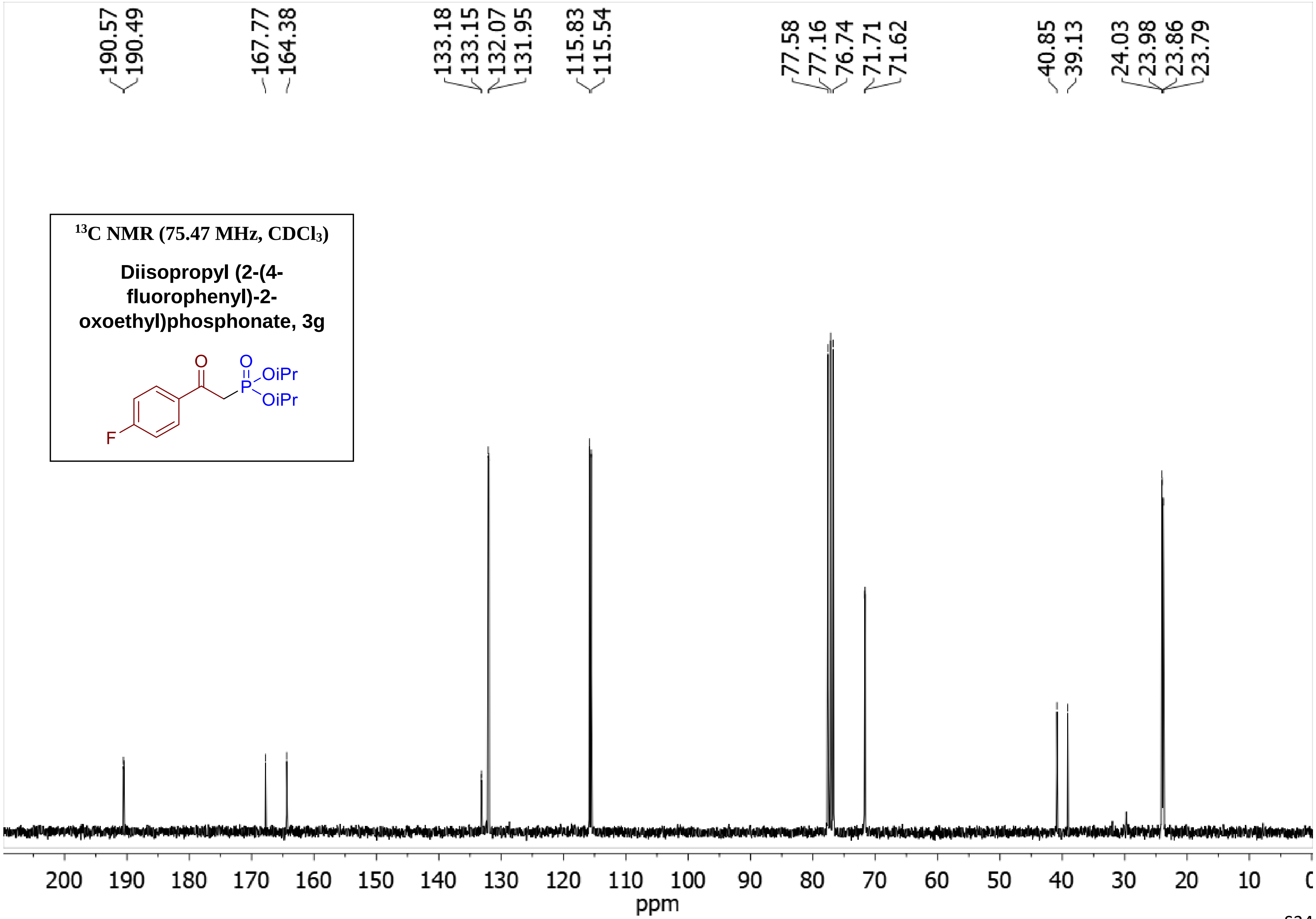
190.57
190.49
167.77
164.38
133.18
133.15
132.07
131.95
115.83
115.54
77.58
77.16
76.74
71.71
71.62
40.85
39.13
24.03
23.98
23.86
23.79
13C NMR (75.47 MHz, CDCl3)
Diisopropyl (2-(4-fluorophenyl)-2-oxoethyl)phosphonate, 3g
O
O
P
OiPr
OiPr
F
200 190 180 170 160 150 140 130 120 110 100 90 80 70 60 50 40 30 20 10
ppm

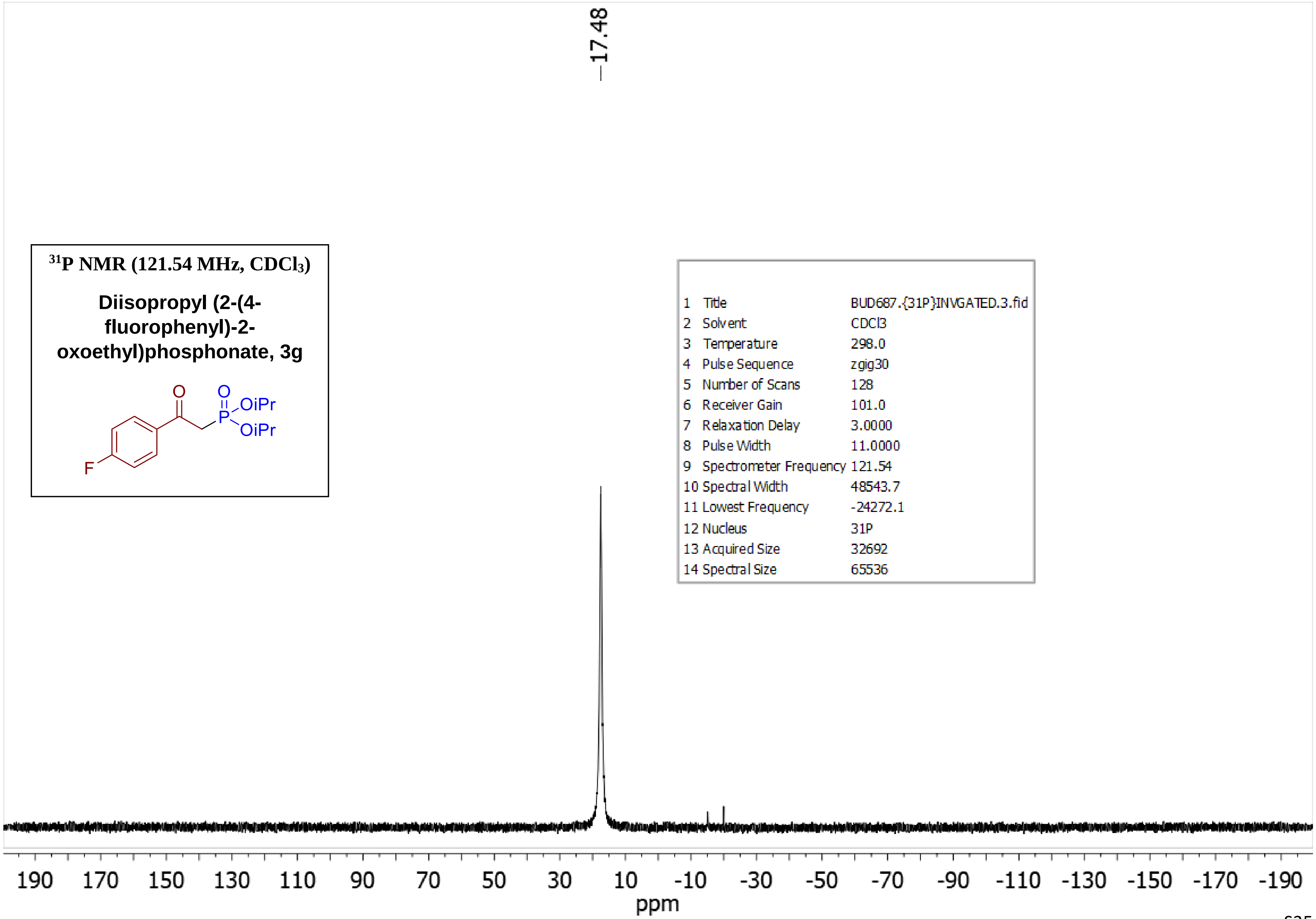
—17.48
^{31}P NMR (121.54 MHz, $CDCl_3$)
Diisopropyl (2-(4-fluorophenyl)-2-oxoethyl)phosphonate, 3g
O
O
OiPr
P
OiPr
F
1 Title BUD687.{31P}INVGATED.3.fid
2 Solvent CDCl3
3 Temperature 298.0
4 Pulse Sequence zgig30
5 Number of Scans 128
6 Receiver Gain 101.0
7 Relaxation Delay 3.0000
8 Pulse Width 11.0000
9 Spectrometer Frequency 121.54
10 Spectral Width 48543.7
11 Lowest Frequency -24272.1
12 Nucleus 31P
13 Acquired Size 32692
14 Spectral Size 65536
190 170 150 130 110 90 70 50 30 10 -10 -30 -50 -70 -90 -110 -130 -150 -170 -190
ppm

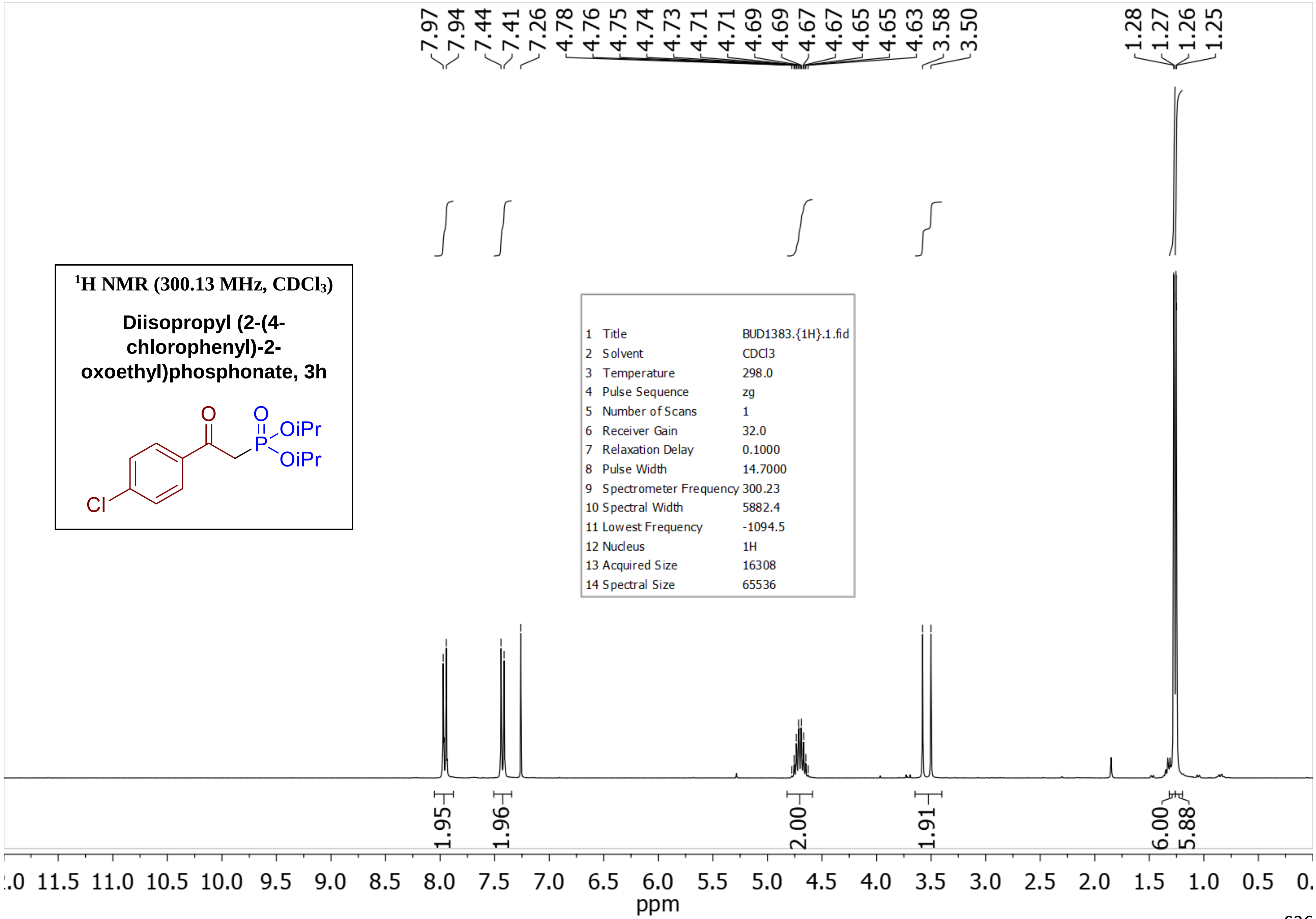

¹H NMR (300.13 MHz, CDCl3)
Diisopropyl (2-(4-chlorophenyl)-2-oxoethyl)phosphonate, 3h
O
O
P
OiPr
OiPr
Cl
7.97
7.94
7.44
7.41
7.26
4.78
4.76
4.75
4.74
4.73
4.71
4.71
4.69
4.69
4.67
4.67
4.65
4.65
4.63
3.58
3.50
1.28
1.27
1.26
1.25
1 Title BUD1383.{1H}.1.fid
2 Solvent CDCl3
3 Temperature 298.0
4 Pulse Sequence zg
5 Number of Scans 1
6 Receiver Gain 32.0
7 Relaxation Delay 0.1000
8 Pulse Width 14.7000
9 Spectrometer Frequency 300.23
10 Spectral Width 5882.4
11 Lowest Frequency -1094.5
12 Nucleus 1H
13 Acquired Size 16308
14 Spectral Size 65536
1.95
1.96
2.00
1.91
6.00
5.88
11.5 11.0 10.5 10.0 9.5 9.0 8.5 8.0 7.5 7.0 6.5 6.0 5.5 5.0 4.5 4.0 3.5 3.0 2.5 2.0 1.5 1.0 0.5
ppm

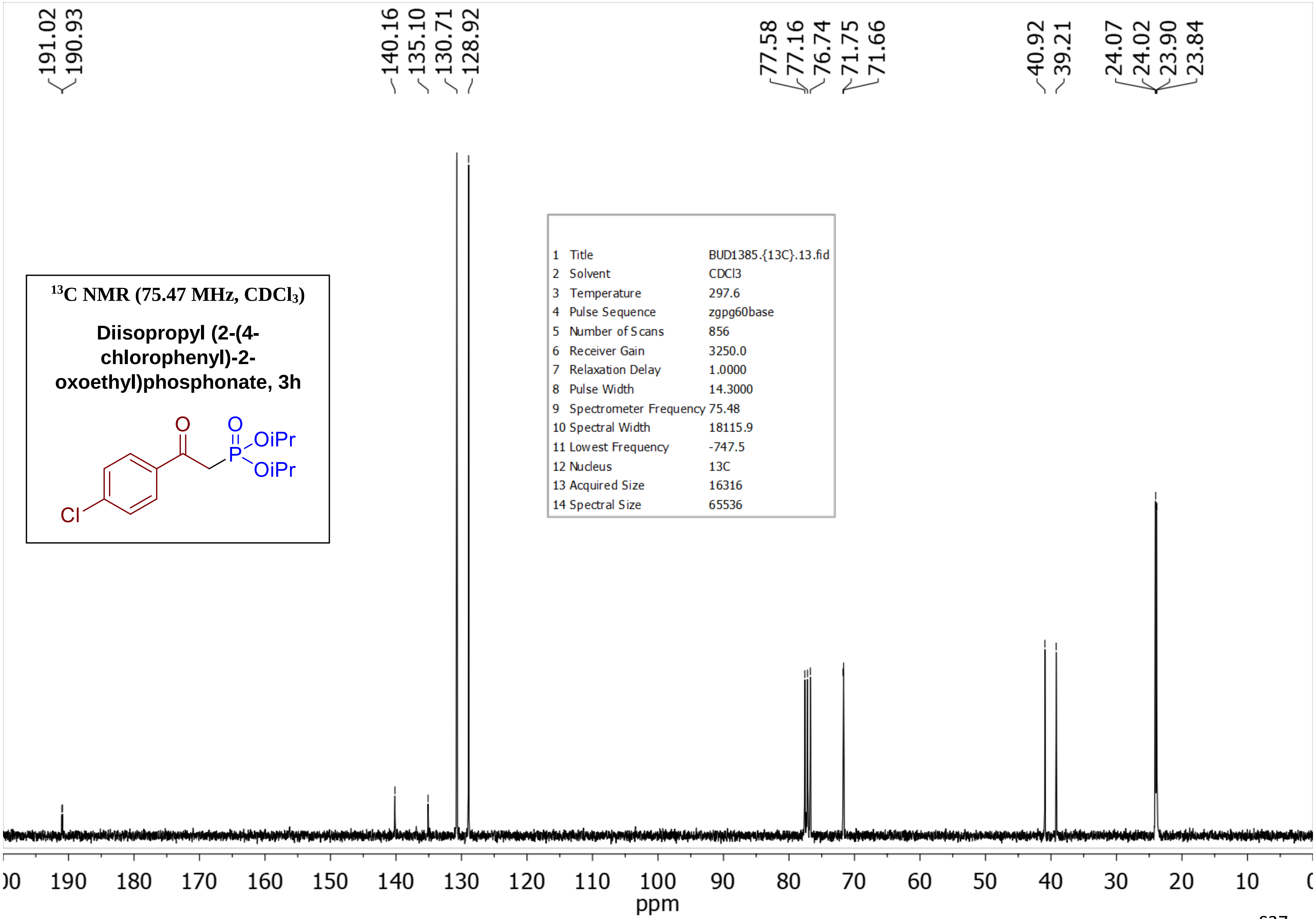
191.02
190.93
140.16
135.10
130.71
128.92
77.58
77.16
76.74
71.75
71.66
40.92
39.21
24.07
24.02
23.90
23.84
13C NMR (75.47 MHz, CDCl3)
Diisopropyl (2-(4-chlorophenyl)-2-oxoethyl)phosphonate, 3h
O
O
P
OiPr
OiPr
Cl
1 Title BUD1385.{13C}.13.fid
2 Solvent CDCl3
3 Temperature 297.6
4 Pulse Sequence zgpg60base
5 Number of Scans 856
6 Receiver Gain 3250.0
7 Relaxation Delay 1.0000
8 Pulse Width 14.3000
9 Spectrometer Frequency 75.48
10 Spectral Width 18115.9
11 Lowest Frequency -747.5
12 Nucleus 13C
13 Acquired Size 16316
14 Spectral Size 65536
190
180
170
160
150
140
130
120
110
100
90
80
70
60
50
40
30
20
10
ppm

^{31}P NMR (121.49 MHz, CDCl$_3$)

Diisopropyl (2-(4-chlorophenyl)-2-oxoethyl)phosphonate, 3h

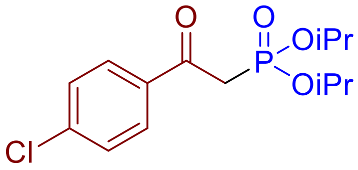

1	Title	BUD1384.{31P}.31.fid
2	Solvent	CDCl3
3	Temperature	297.8
4	Pulse Sequence	zgpg30
5	Number of Scans	464
6	Receiver Gain	2050.0
7	Relaxation Delay	2.0000
8	Pulse Width	14.7000
9	Spectrometer Frequency	121.49
10	Spectral Width	96153.8
11	Lowest Frequency	-54043.6
12	Nucleus	31P
13	Acquired Size	32700
14	Spectral Size	65536

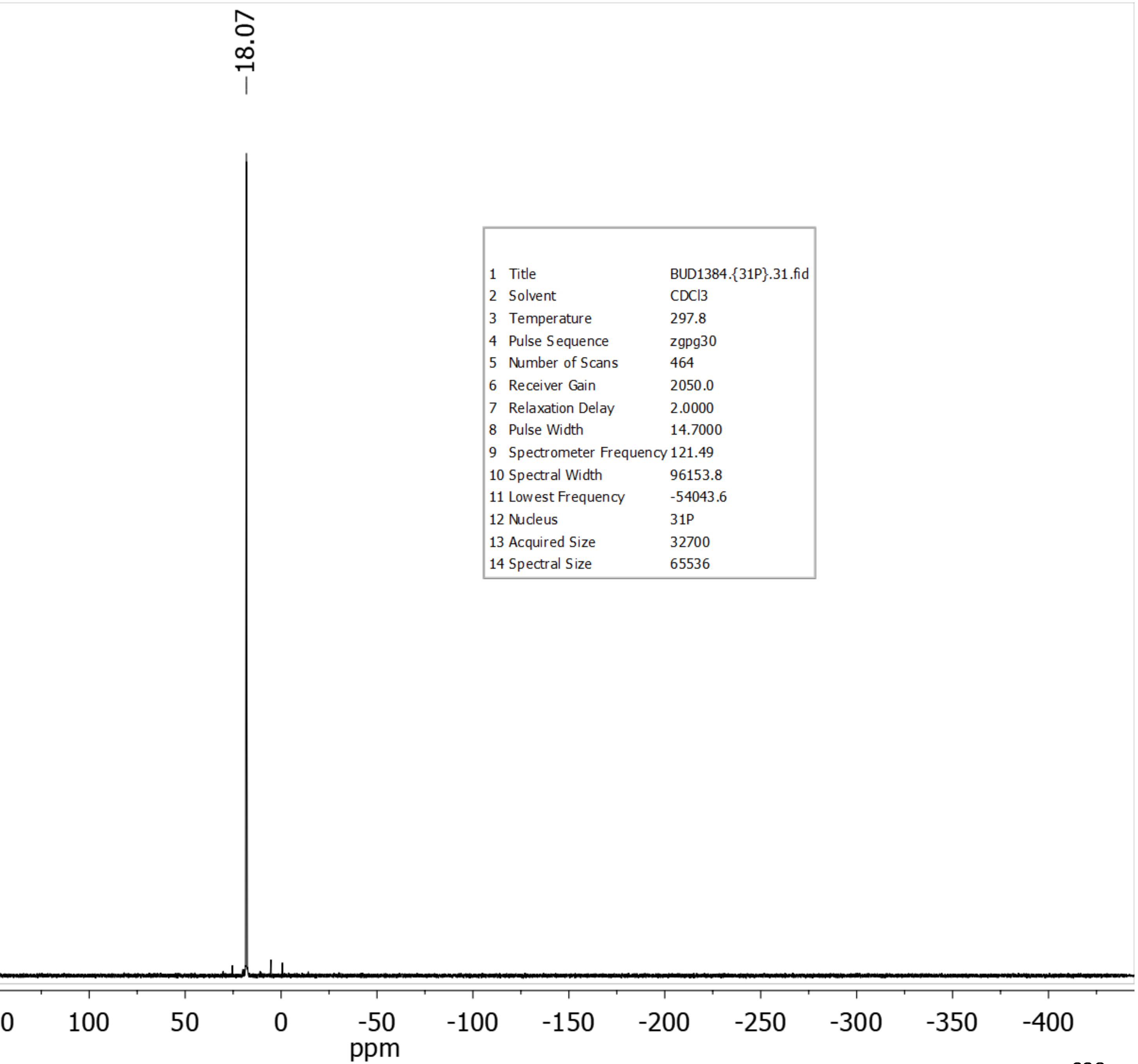

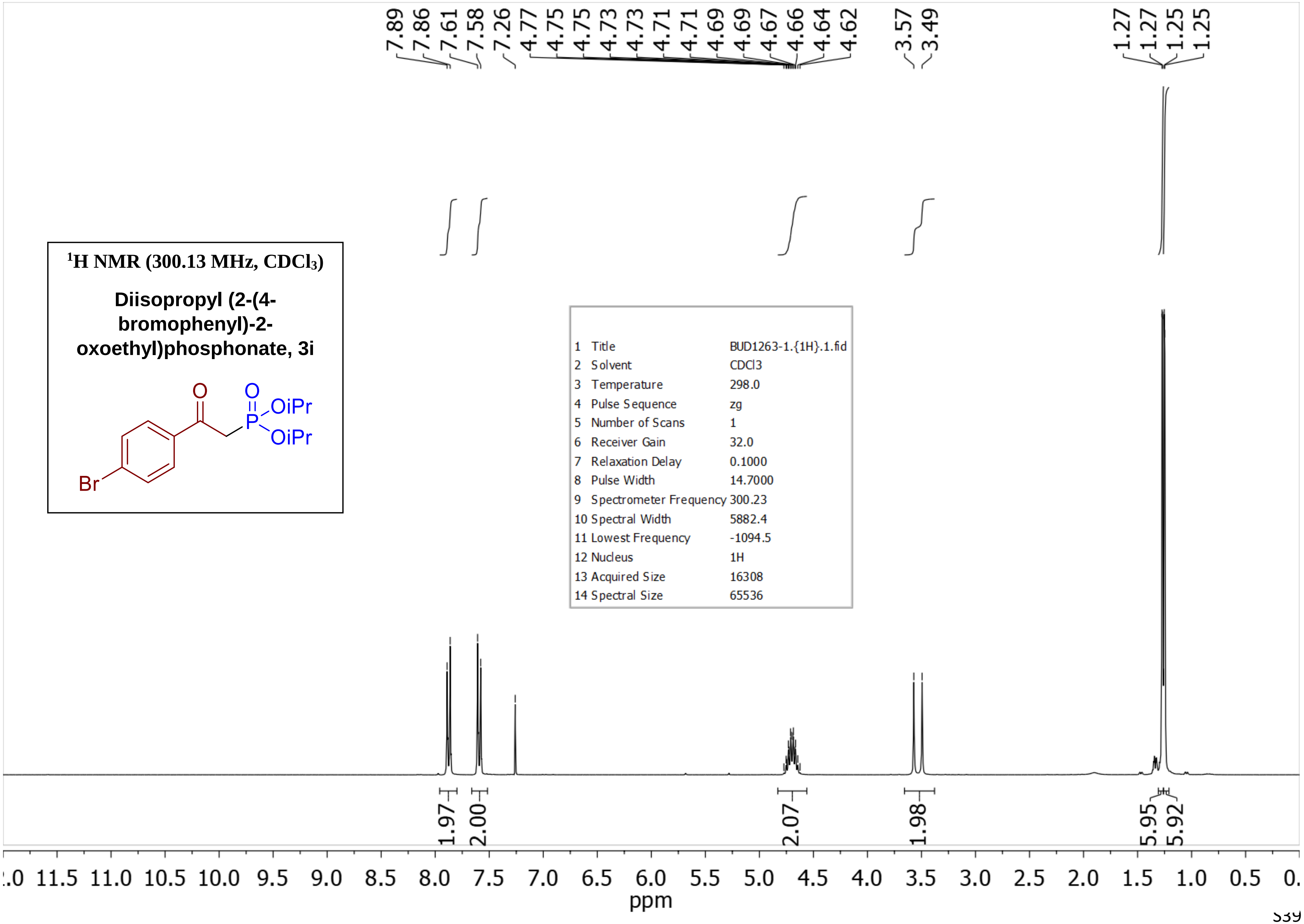

1	Title	BUD1263-1.{1H}.1.fid
2	Solvent	CDCl3
3	Temperature	298.0
4	Pulse Sequence	zg
5	Number of Scans	1
6	Receiver Gain	32.0
7	Relaxation Delay	0.1000
8	Pulse Width	14.7000
9	Spectrometer Frequency	300.23
10	Spectral Width	5882.4
11	Lowest Frequency	-1094.5
12	Nucleus	1H
13	Acquired Size	16308
14	Spectral Size	65536

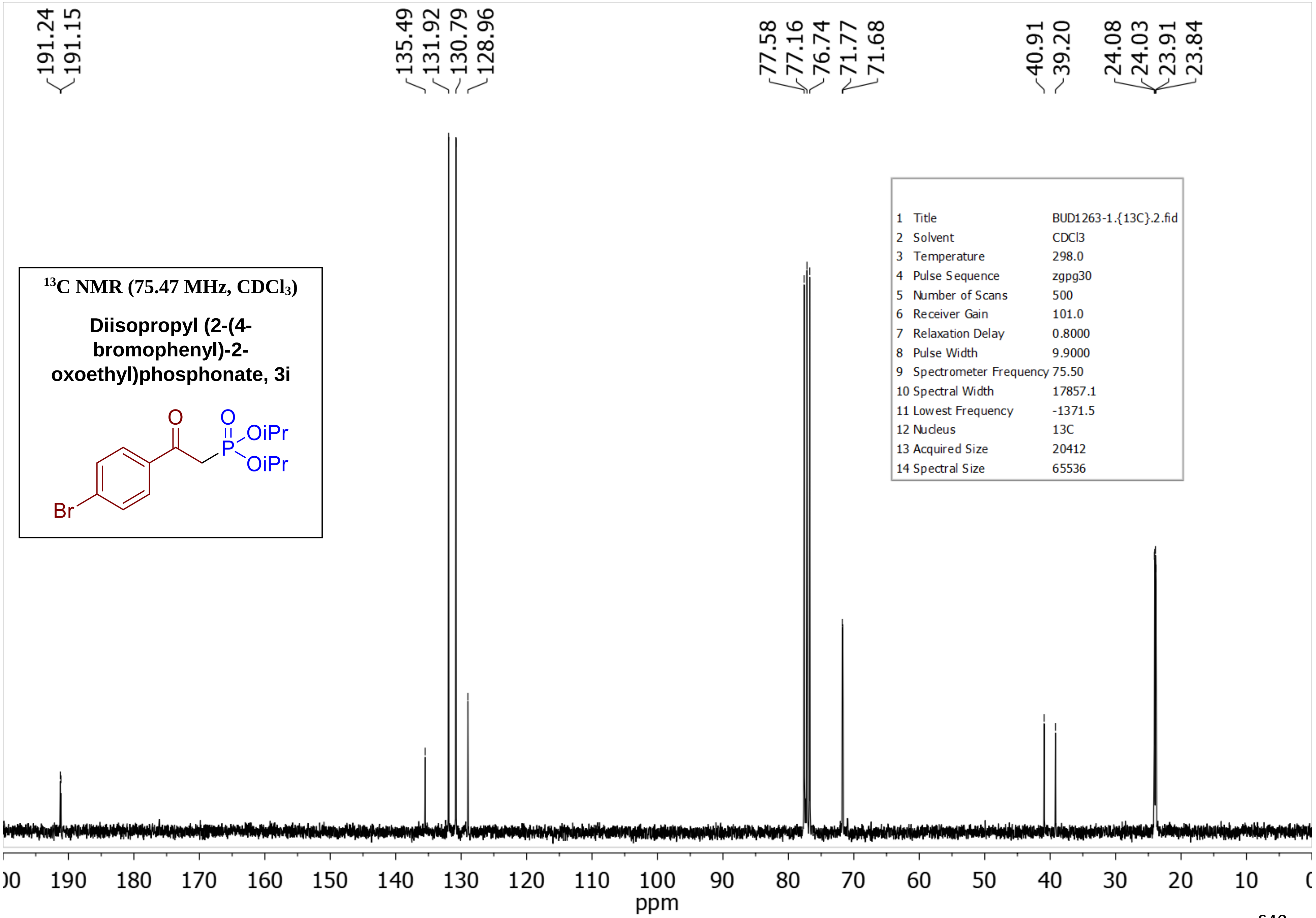
191.24
191.15
135.49
131.92
130.79
128.96
77.58
77.16
76.74
71.77
71.68
40.91
39.20
24.08
24.03
23.91
23.84
13C NMR (75.47 MHz, CDCl3)
Diisopropyl (2-(4-bromophenyl)-2-oxoethyl)phosphonate, 3i
O
O
OiPr
P
OiPr
Br
1 Title BUD1263-1.{13C}.2.fid
2 Solvent CDCl3
3 Temperature 298.0
4 Pulse Sequence zgpg30
5 Number of Scans 500
6 Receiver Gain 101.0
7 Relaxation Delay 0.8000
8 Pulse Width 9.9000
9 Spectrometer Frequency 75.50
10 Spectral Width 17857.1
11 Lowest Frequency -1371.5
12 Nucleus 13C
13 Acquired Size 20412
14 Spectral Size 65536
190
180
170
160
150
140
130
120
110
100
90
80
70
60
50
40
30
20
10
ppm

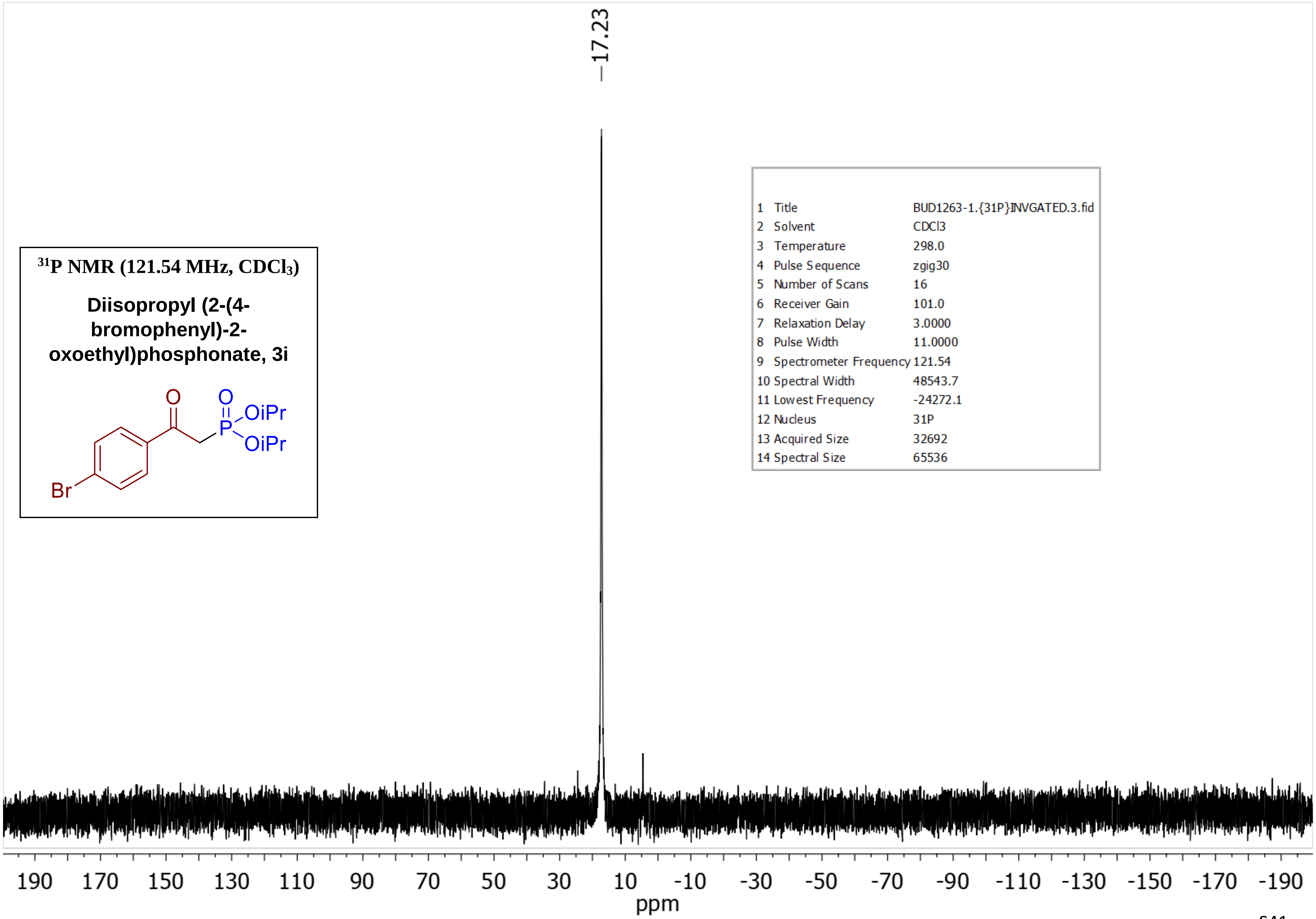

17.23
31P NMR (121.54 MHz, CDCl3)
Diisopropyl (2-(4-bromophenyl)-2-oxoethyl)phosphonate, 3i
O
O
OiPr
P
OiPr
Br
1 Title BUD1263-1.{31P}INVGATED.3.fid
2 Solvent CDCl3
3 Temperature 298.0
4 Pulse Sequence zgig30
5 Number of Scans 16
6 Receiver Gain 101.0
7 Relaxation Delay 3.0000
8 Pulse Width 11.0000
9 Spectrometer Frequency 121.54
10 Spectral Width 48543.7
11 Lowest Frequency -24272.1
12 Nucleus 31P
13 Acquired Size 32692
14 Spectral Size 65536
190 170 150 130 110 90 70 50 30 10 -10 -30 -50 -70 -90 -110 -130 -150 -170 -190
ppm

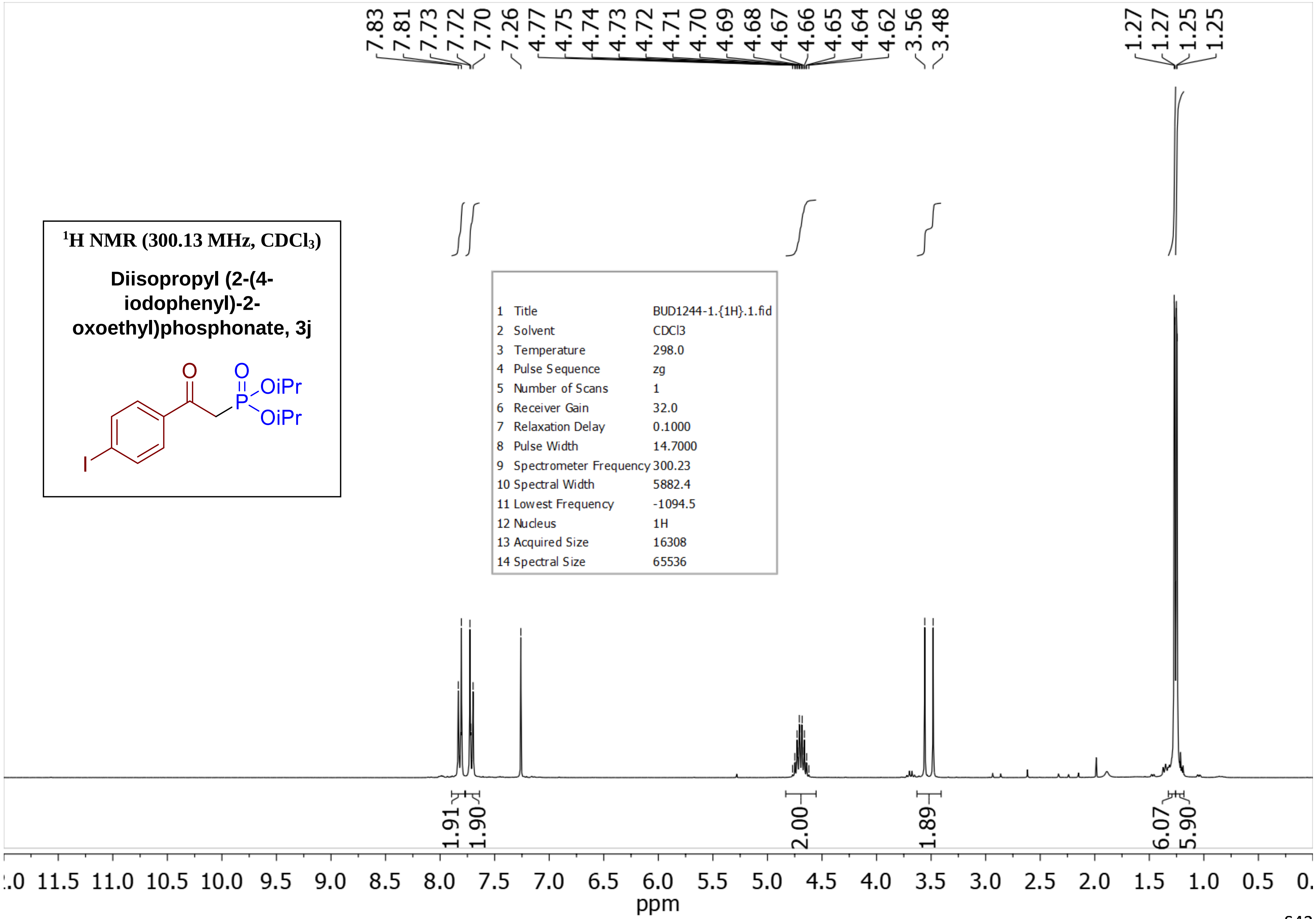
7.83 7.81 7.73 7.72 7.70 7.26 4.77 4.75 4.74 4.73 4.72 4.71 4.70 4.69 4.68 4.67 4.66 4.65 4.64 4.62 3.56 3.48 1.27 1.27 1.25 1.25
1H NMR (300.13 MHz, CDCl3)
Diisopropyl (2-(4-iodophenyl)-2-oxoethyl)phosphonate, 3j
O
O
OiPr
P
OiPr
I
1 Title BUD1244-1.{1H}.1.fid
2 Solvent CDCl3
3 Temperature 298.0
4 Pulse Sequence zg
5 Number of Scans 1
6 Receiver Gain 32.0
7 Relaxation Delay 0.1000
8 Pulse Width 14.7000
9 Spectrometer Frequency 300.23
10 Spectral Width 5882.4
11 Lowest Frequency -1094.5
12 Nucleus 1H
13 Acquired Size 16308
14 Spectral Size 65536
1.91 1.90 2.00 1.89 6.07 5.90
11.5 11.0 10.5 10.0 9.5 9.0 8.5 8.0 7.5 7.0 6.5 6.0 5.5 5.0 4.5 4.0 3.5 3.0 2.5 2.0 1.5 1.0 0.5
ppm

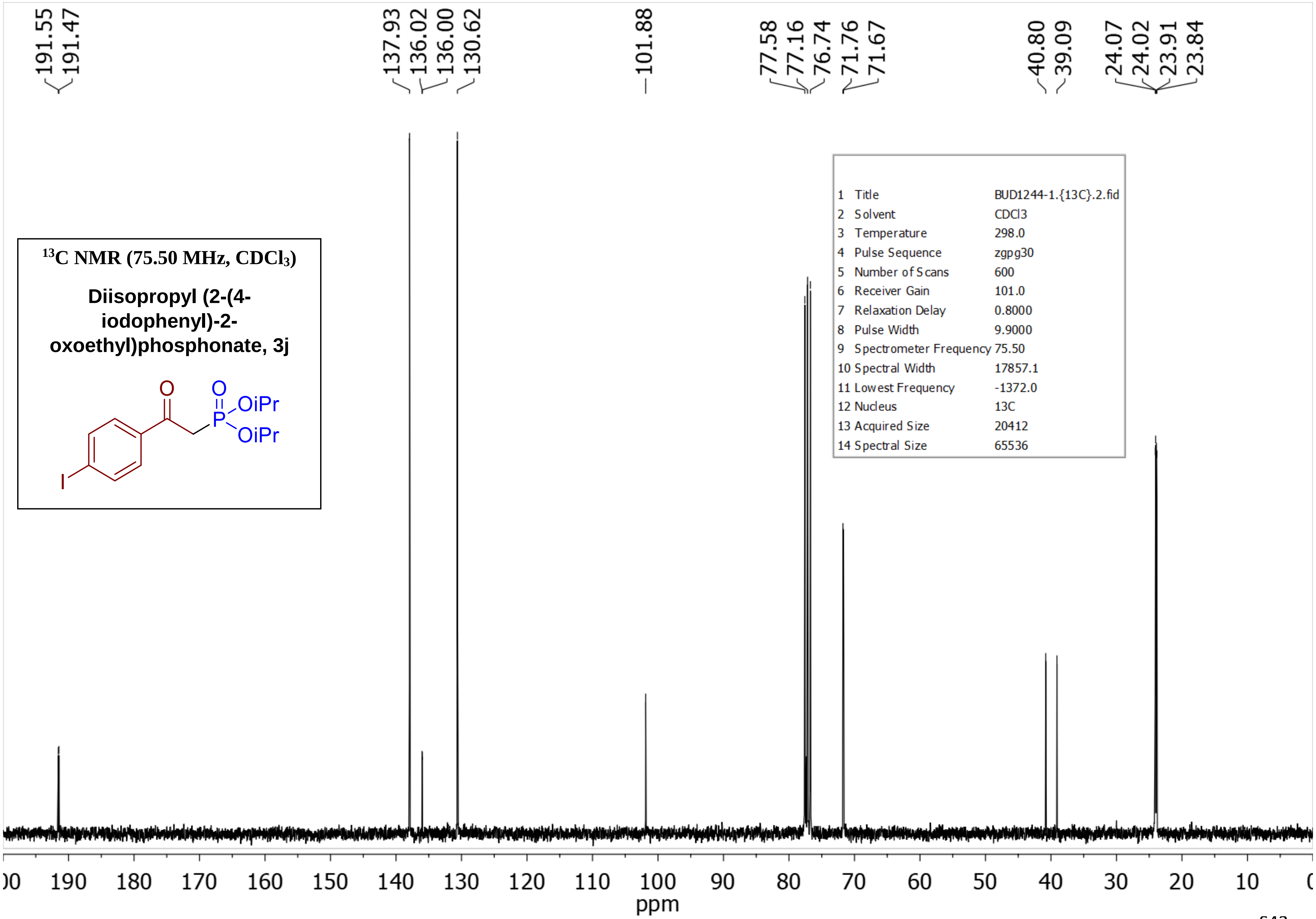
191.55
191.47
137.93
136.02
136.00
130.62
101.88
77.58
77.16
76.74
71.76
71.67
40.80
39.09
24.07
24.02
23.91
23.84
^{13}C NMR (75.50 MHz, $CDCl_3$)
Diisopropyl (2-(4-iodophenyl)-2-oxoethyl)phosphonate, 3j
O
O
P
OiPr
OiPr
I
1 Title BUD1244-1.{13C}.2.fid
2 Solvent CDCl3
3 Temperature 298.0
4 Pulse Sequence zgpg30
5 Number of Scans 600
6 Receiver Gain 101.0
7 Relaxation Delay 0.8000
8 Pulse Width 9.9000
9 Spectrometer Frequency 75.50
10 Spectral Width 17857.1
11 Lowest Frequency -1372.0
12 Nucleus 13C
13 Acquired Size 20412
14 Spectral Size 65536
190
180
170
160
150
140
130
120
110
100
90
80
70
60
50
40
30
20
10
ppm

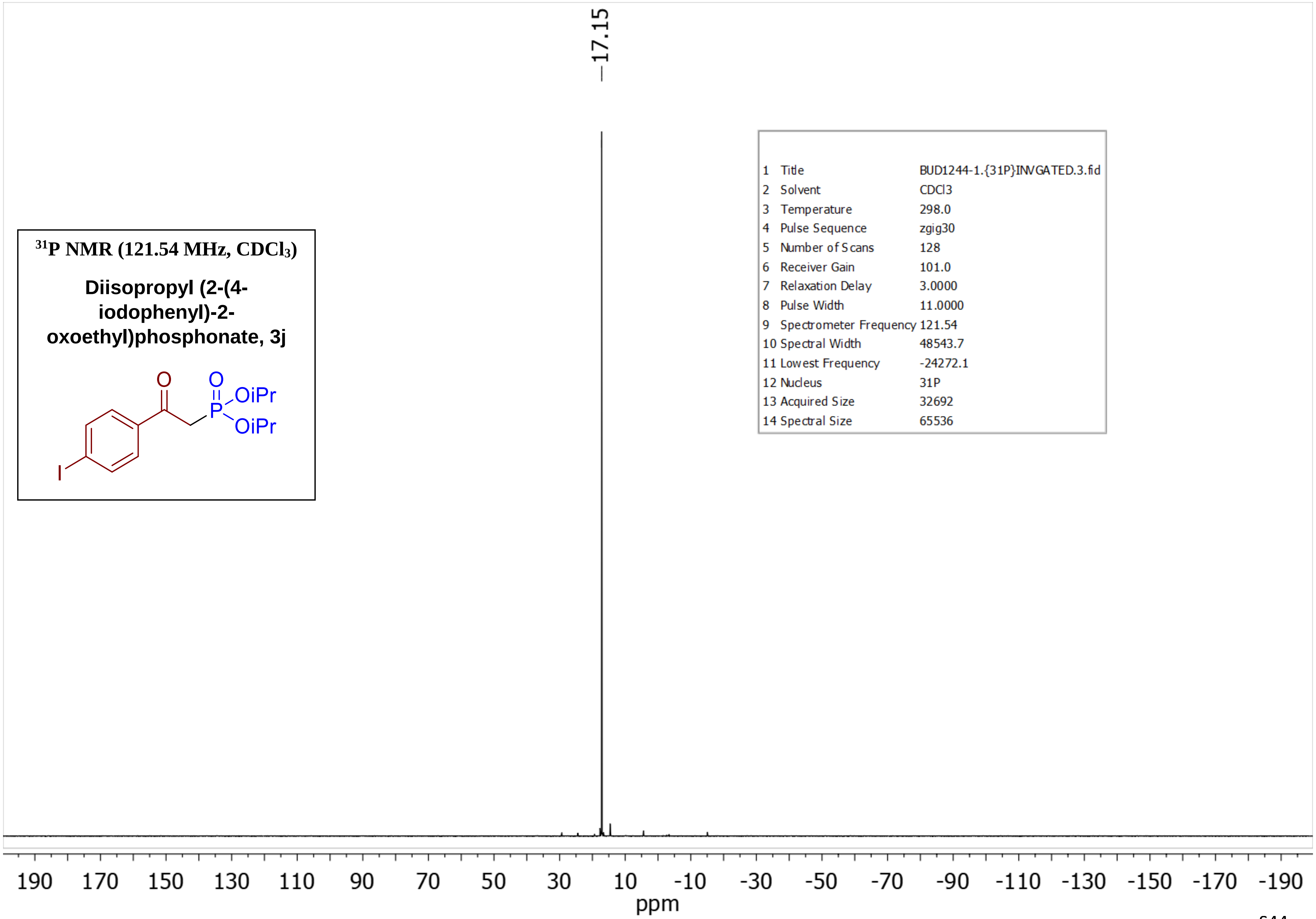
17.15
31P NMR (121.54 MHz, CDCl3)
Diisopropyl (2-(4-iodophenyl)-2-oxoethyl)phosphonate, 3j
O
O
OiPr
P
OiPr
I
1 Title BUD1244-1.{31P}INVGATED.3.fid
2 Solvent CDCl3
3 Temperature 298.0
4 Pulse Sequence zgig30
5 Number of Scans 128
6 Receiver Gain 101.0
7 Relaxation Delay 3.0000
8 Pulse Width 11.0000
9 Spectrometer Frequency 121.54
10 Spectral Width 48543.7
11 Lowest Frequency -24272.1
12 Nucleus 31P
13 Acquired Size 32692
14 Spectral Size 65536
190 170 150 130 110 90 70 50 30 10 -10 -30 -50 -70 -90 -110 -130 -150 -170 -190
ppm

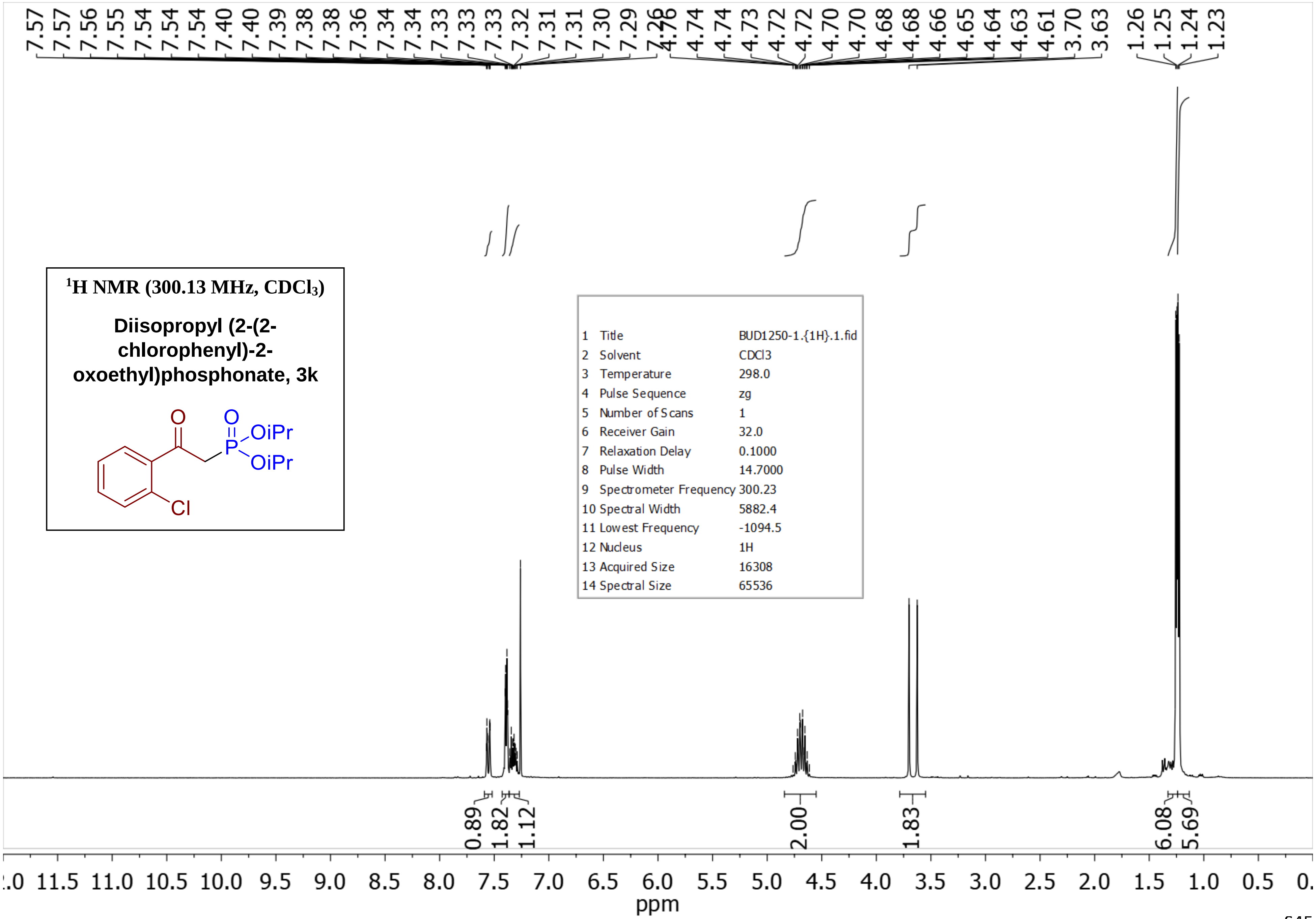

7.57 7.57 7.56 7.55 7.54 7.54 7.54 7.40 7.40 7.39 7.38 7.38 7.36 7.34 7.34 7.33 7.33 7.33 7.32 7.31 7.31 7.30 7.29 7.26 4.76 4.74 4.74 4.73 4.72 4.72 4.70 4.70 4.68 4.68 4.66 4.65 4.64 4.63 4.61 3.70 3.63 1.26 1.25 1.24 1.23
1H NMR (300.13 MHz, CDCl3)
Diisopropyl (2-(2-chlorophenyl)-2-oxoethyl)phosphonate, 3k
O
O
OiPr
P
OiPr
Cl
1 Title BUD1250-1.{1H}.1.fid
2 Solvent CDCl3
3 Temperature 298.0
4 Pulse Sequence zg
5 Number of Scans 1
6 Receiver Gain 32.0
7 Relaxation Delay 0.1000
8 Pulse Width 14.7000
9 Spectrometer Frequency 300.23
10 Spectral Width 5882.4
11 Lowest Frequency -1094.5
12 Nucleus 1H
13 Acquired Size 16308
14 Spectral Size 65536
0.89 1.82 1.12 2.00 1.83 6.08 5.69
11.5 11.0 10.5 10.0 9.5 9.0 8.5 8.0 7.5 7.0 6.5 6.0 5.5 5.0 4.5 4.0 3.5 3.0 2.5 2.0 1.5 1.0 0.5
ppm

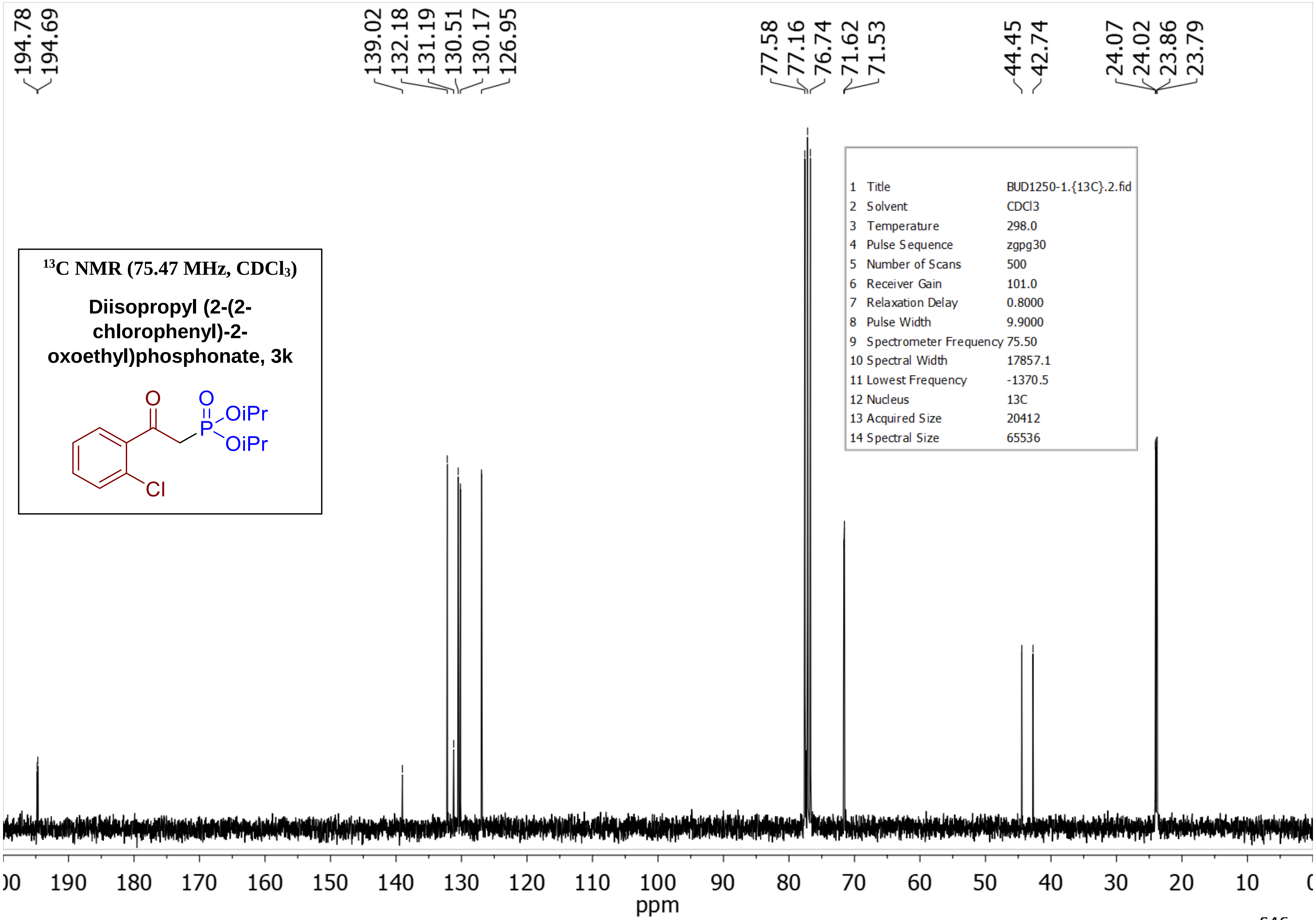
194.78
194.69
139.02
132.18
131.19
130.51
130.17
126.95
77.58
77.16
76.74
71.62
71.53
44.45
42.74
24.07
24.02
23.86
23.79
^{13}C NMR (75.47 MHz, CDCl$_3$)
Diisopropyl (2-(2-chlorophenyl)-2-oxoethyl)phosphonate, 3k
O
O
P
OiPr
OiPr
Cl
1 Title BUD1250-1.{13C}.2.fid
2 Solvent CDCl3
3 Temperature 298.0
4 Pulse Sequence zgpg30
5 Number of Scans 500
6 Receiver Gain 101.0
7 Relaxation Delay 0.8000
8 Pulse Width 9.9000
9 Spectrometer Frequency 75.50
10 Spectral Width 17857.1
11 Lowest Frequency -1370.5
12 Nucleus 13C
13 Acquired Size 20412
14 Spectral Size 65536
190
180
170
160
150
140
130
120
110
100
90
80
70
60
50
40
30
20
10
ppm

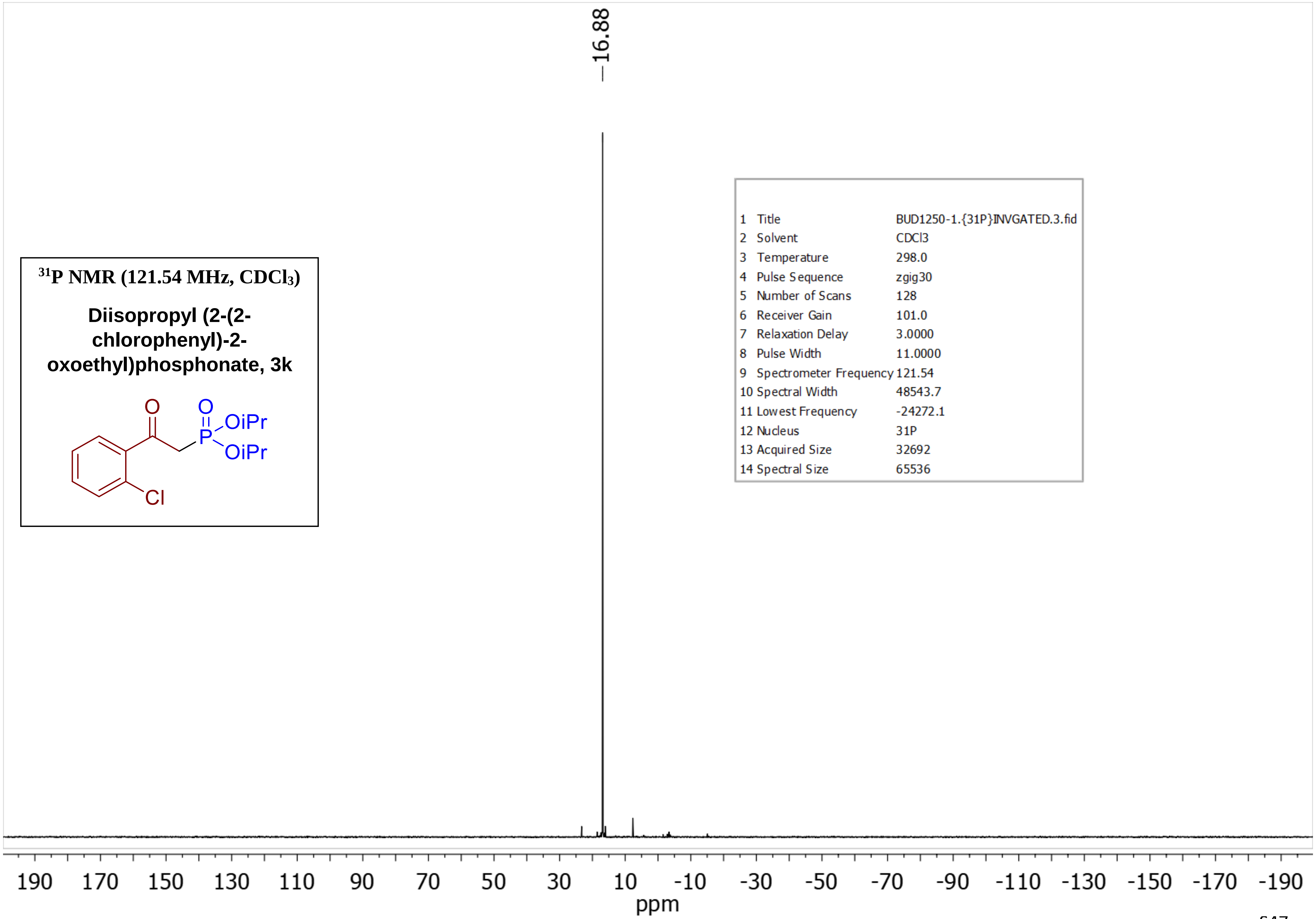

31P NMR (121.54 MHz, CDCl3)
Diisopropyl (2-(2-chlorophenyl)-2-oxoethyl)phosphonate, 3k
O
O
OiPr
P
OiPr
Cl
—16.88
1 Title BUD1250-1.{31P}INVGATED.3.fid
2 Solvent CDCl3
3 Temperature 298.0
4 Pulse Sequence zgig30
5 Number of Scans 128
6 Receiver Gain 101.0
7 Relaxation Delay 3.0000
8 Pulse Width 11.0000
9 Spectrometer Frequency 121.54
10 Spectral Width 48543.7
11 Lowest Frequency -24272.1
12 Nucleus 31P
13 Acquired Size 32692
14 Spectral Size 65536
190 170 150 130 110 90 70 50 30 10 -10 -30 -50 -70 -90 -110 -130 -150 -170 -190
ppm

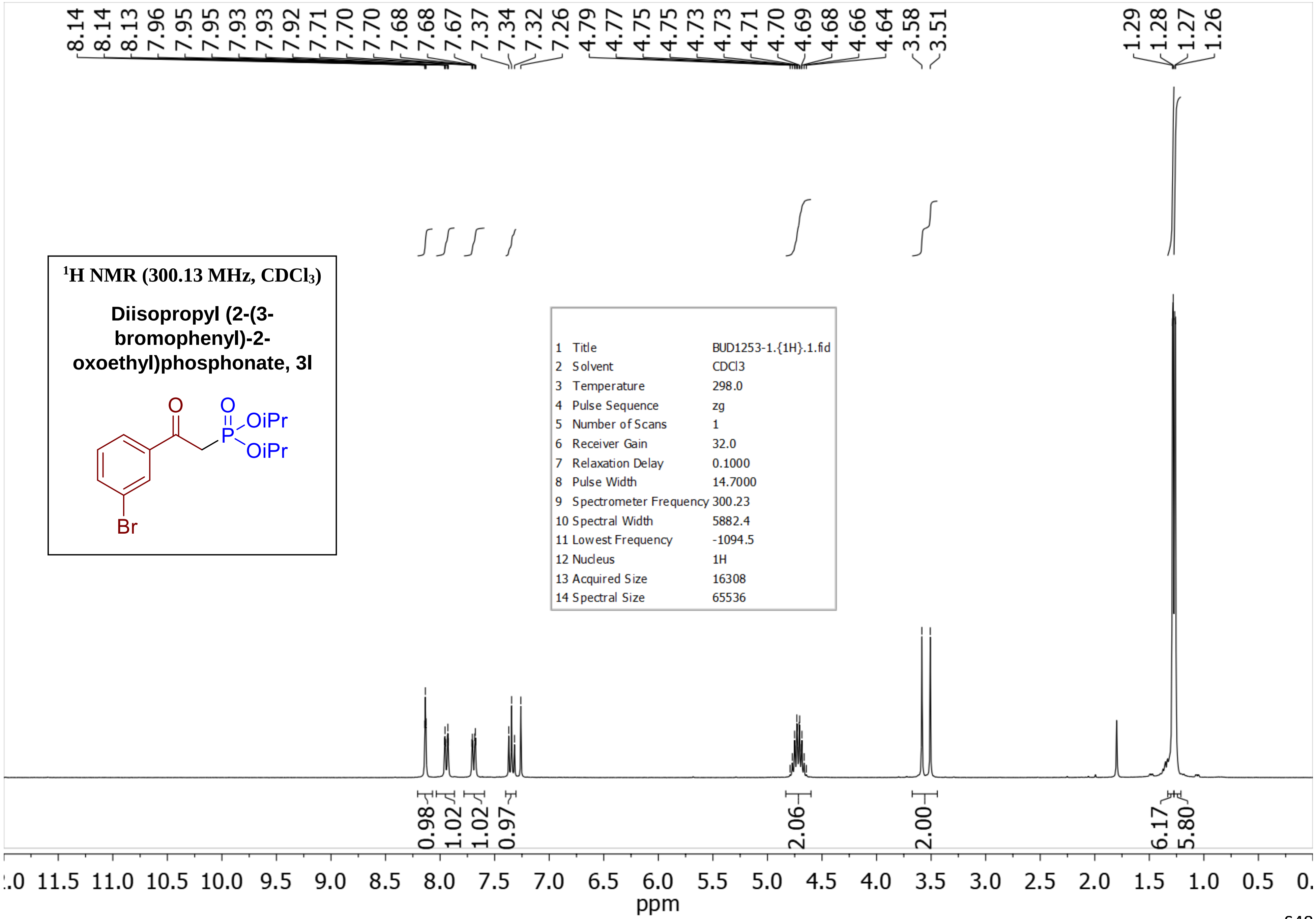

^{1}H NMR (300.13 MHz, CDCl$_3$)
Diisopropyl (2-(3-bromophenyl)-2-oxoethyl)phosphonate, 3l
O
O
OiPr
P
OiPr
Br
1 Title BUD1253-1.{1H}.1.fid
2 Solvent CDCl3
3 Temperature 298.0
4 Pulse Sequence zg
5 Number of Scans 1
6 Receiver Gain 32.0
7 Relaxation Delay 0.1000
8 Pulse Width 14.7000
9 Spectrometer Frequency 300.23
10 Spectral Width 5882.4
11 Lowest Frequency -1094.5
12 Nucleus 1H
13 Acquired Size 16308
14 Spectral Size 65536
8.14 8.14 8.13 7.96 7.95 7.95 7.93 7.93 7.92 7.71 7.70 7.70 7.68 7.68 7.67 7.37 7.34 7.32 7.26 4.79 4.77 4.75 4.75 4.73 4.73 4.71 4.70 4.69 4.68 4.66 4.64 3.58 3.51 1.29 1.28 1.27 1.26
0.98 1.02 1.02 0.97 2.06 2.00 6.17 5.80
11.5 11.0 10.5 10.0 9.5 9.0 8.5 8.0 7.5 7.0 6.5 6.0 5.5 5.0 4.5 4.0 3.5 3.0 2.5 2.0 1.5 1.0 0.5
ppm

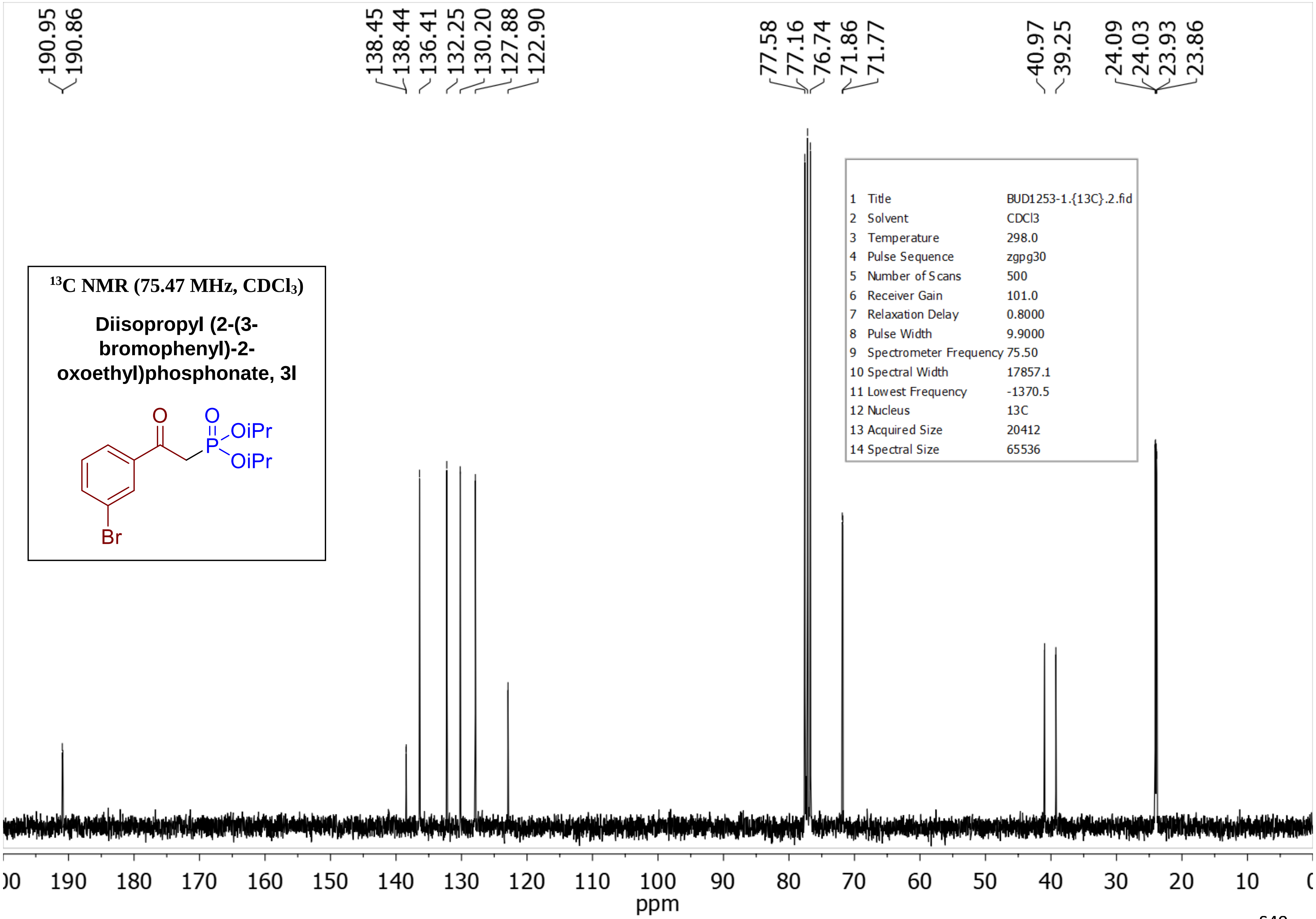
190.95
190.86
138.45
138.44
136.41
132.25
130.20
127.88
122.90
77.58
77.16
76.74
71.86
71.77
40.97
39.25
24.09
24.03
23.93
23.86
13C NMR (75.47 MHz, CDCl3)
Diisopropyl (2-(3-bromophenyl)-2-oxoethyl)phosphonate, 3l
O
O
P
OiPr
OiPr
Br
1 Title BUD1253-1.{13C}.2.fid
2 Solvent CDCl3
3 Temperature 298.0
4 Pulse Sequence zgpg30
5 Number of Scans 500
6 Receiver Gain 101.0
7 Relaxation Delay 0.8000
8 Pulse Width 9.9000
9 Spectrometer Frequency 75.50
10 Spectral Width 17857.1
11 Lowest Frequency -1370.5
12 Nucleus 13C
13 Acquired Size 20412
14 Spectral Size 65536
190
180
170
160
150
140
130
120
110
100
90
80
70
60
50
40
30
20
10
ppm

^{31}P NMR (121.54 MHz, $CDCl_3$)

Diisopropyl (2-(3-bromophenyl)-2-oxoethyl)phosphonate, 3l

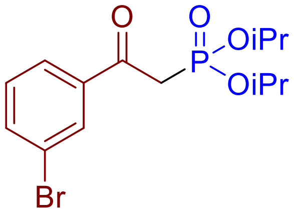

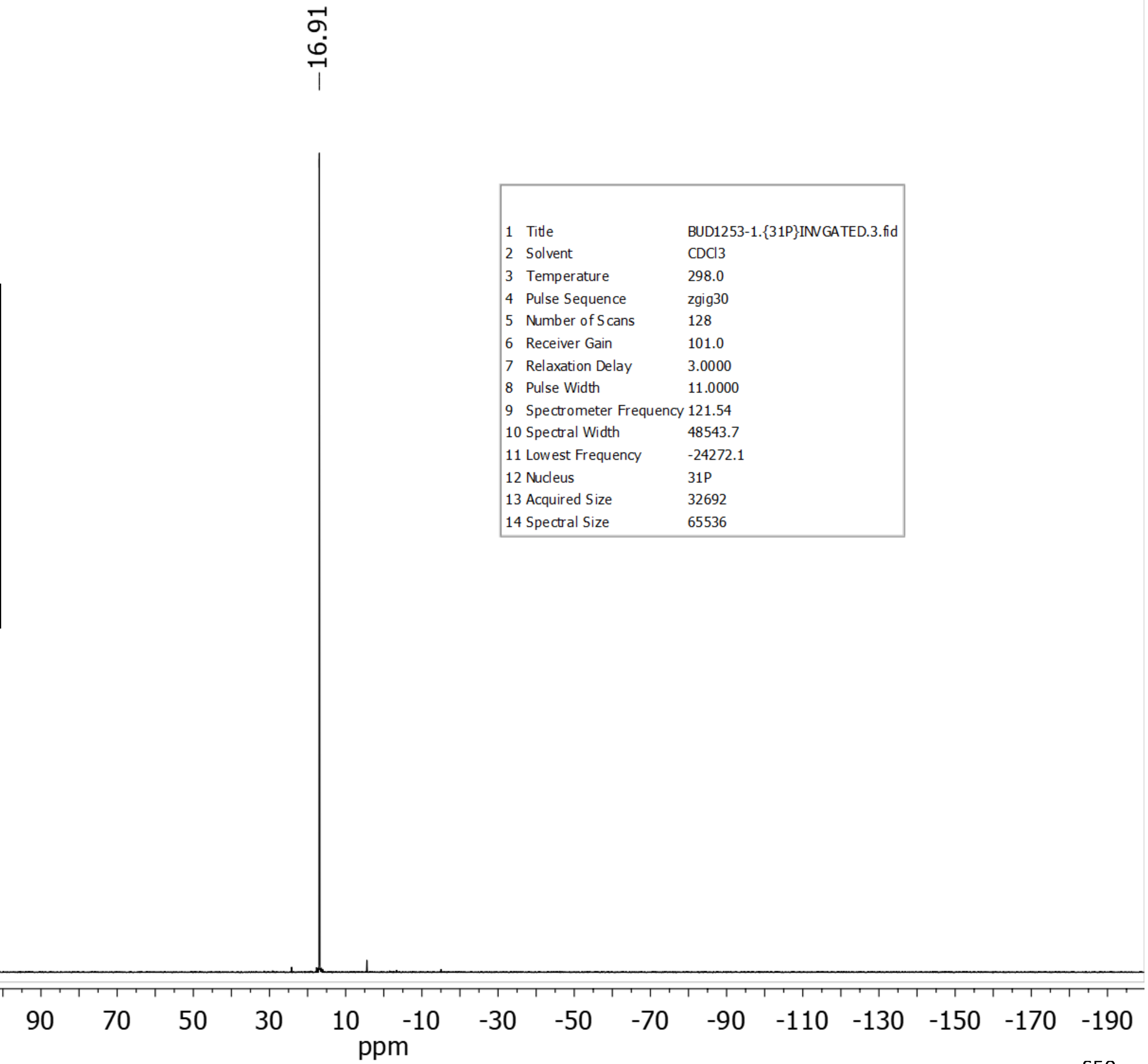

1	Title	BUD1253-1.{31P}INVGATED.3.fid
2	Solvent	CDCl3
3	Temperature	298.0
4	Pulse Sequence	zgig30
5	Number of Scans	128
6	Receiver Gain	101.0
7	Relaxation Delay	3.0000
8	Pulse Width	11.0000
9	Spectrometer Frequency	121.54
10	Spectral Width	48543.7
11	Lowest Frequency	-24272.1
12	Nucleus	31P
13	Acquired Size	32692
14	Spectral Size	65536

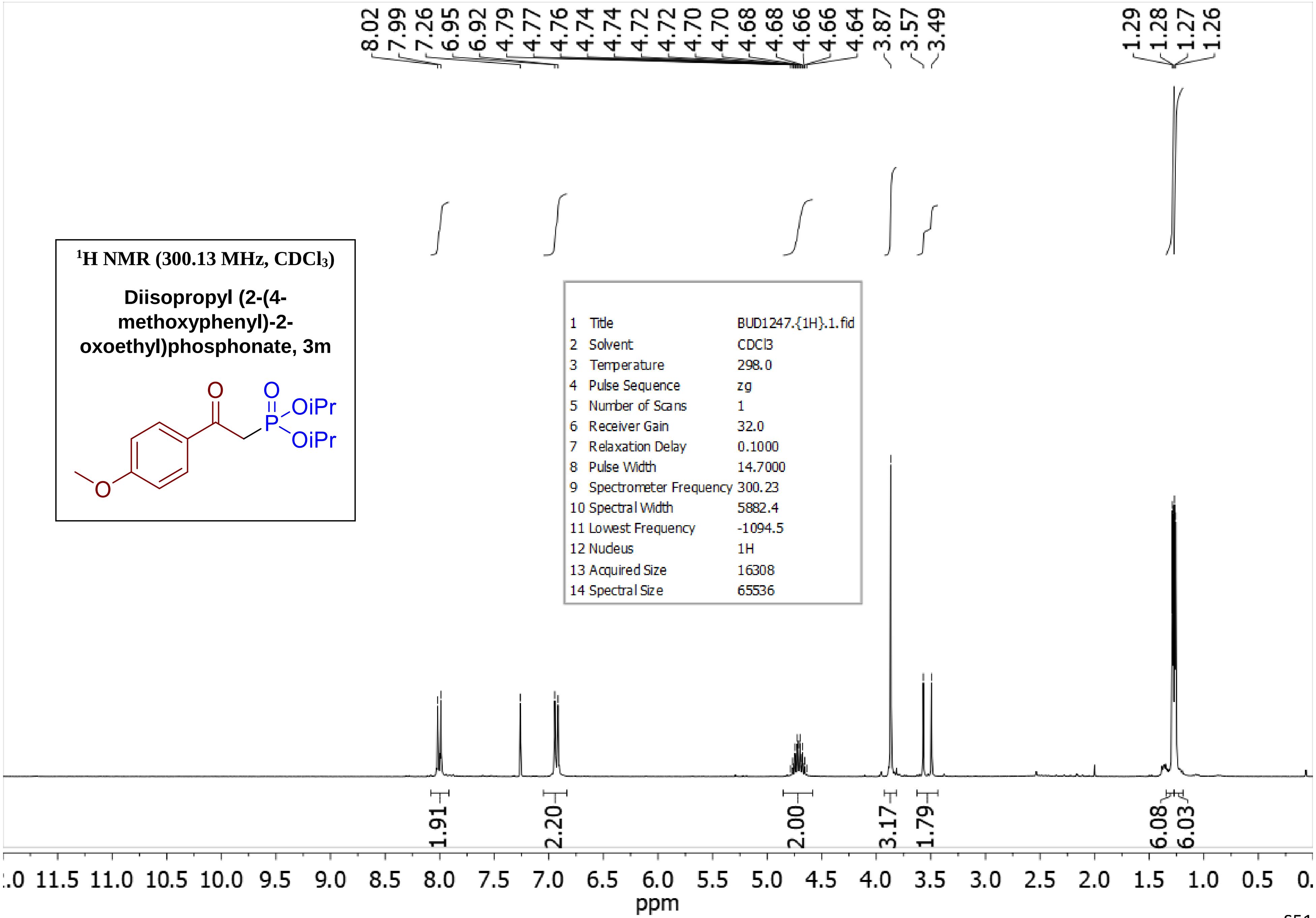
8.02 7.99 7.26 6.95 6.92 4.79 4.77 4.76 4.74 4.74 4.72 4.72 4.70 4.70 4.68 4.68 4.66 4.66 4.64 3.87 3.57 3.49
1.29 1.28 1.27 1.26
^{1}H NMR (300.13 MHz, CDCl3)
Diisopropyl (2-(4-methoxyphenyl)-2-oxoethyl)phosphonate, 3m
O
O
OiPr
P
OiPr
O
1 Title BUD1247.{1H}.1.fid
2 Solvent CDCl3
3 Temperature 298.0
4 Pulse Sequence zg
5 Number of Scans 1
6 Receiver Gain 32.0
7 Relaxation Delay 0.1000
8 Pulse Width 14.7000
9 Spectrometer Frequency 300.23
10 Spectral Width 5882.4
11 Lowest Frequency -1094.5
12 Nucleus 1H
13 Acquired Size 16308
14 Spectral Size 65536
1.91
2.20
2.00
3.17
1.79
6.08
6.03
11.5 11.0 10.5 10.0 9.5 9.0 8.5 8.0 7.5 7.0 6.5 6.0 5.5 5.0 4.5 4.0 3.5 3.0 2.5 2.0 1.5 1.0 0.5
ppm

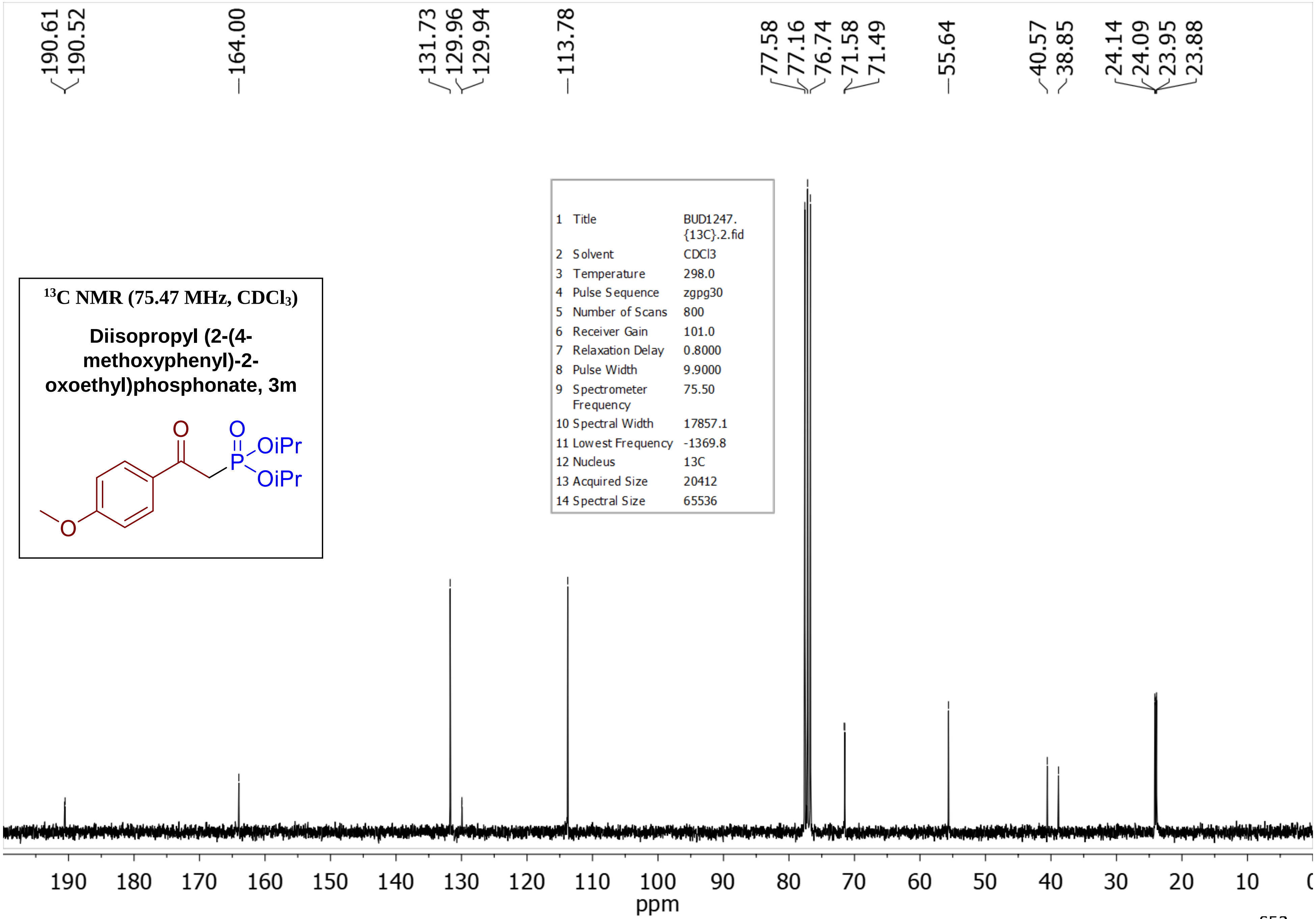
190.61
190.52
164.00
131.73
129.96
129.94
113.78
77.58
77.16
76.74
71.58
71.49
55.64
40.57
38.85
24.14
24.09
23.95
23.88
^{13}C NMR (75.47 MHz, CDCl$_3$)
Diisopropyl (2-(4-methoxyphenyl)-2-oxoethyl)phosphonate, 3m
O
O
OiPr
OiPr
P
O
1 Title BUD1247.{13C}.2.fid
2 Solvent CDCl3
3 Temperature 298.0
4 Pulse Sequence zgpg30
5 Number of Scans 800
6 Receiver Gain 101.0
7 Relaxation Delay 0.8000
8 Pulse Width 9.9000
9 Spectrometer Frequency 75.50
10 Spectral Width 17857.1
11 Lowest Frequency -1369.8
12 Nucleus 13C
13 Acquired Size 20412
14 Spectral Size 65536
190
180
170
160
150
140
130
120
110
100
90
80
70
60
50
40
30
20
10
ppm

^{31}P NMR (121.54 MHz, $CDCl_3$)

Diisopropyl (2-(4-methoxyphenyl)-2-oxoethyl)phosphonate, 3m

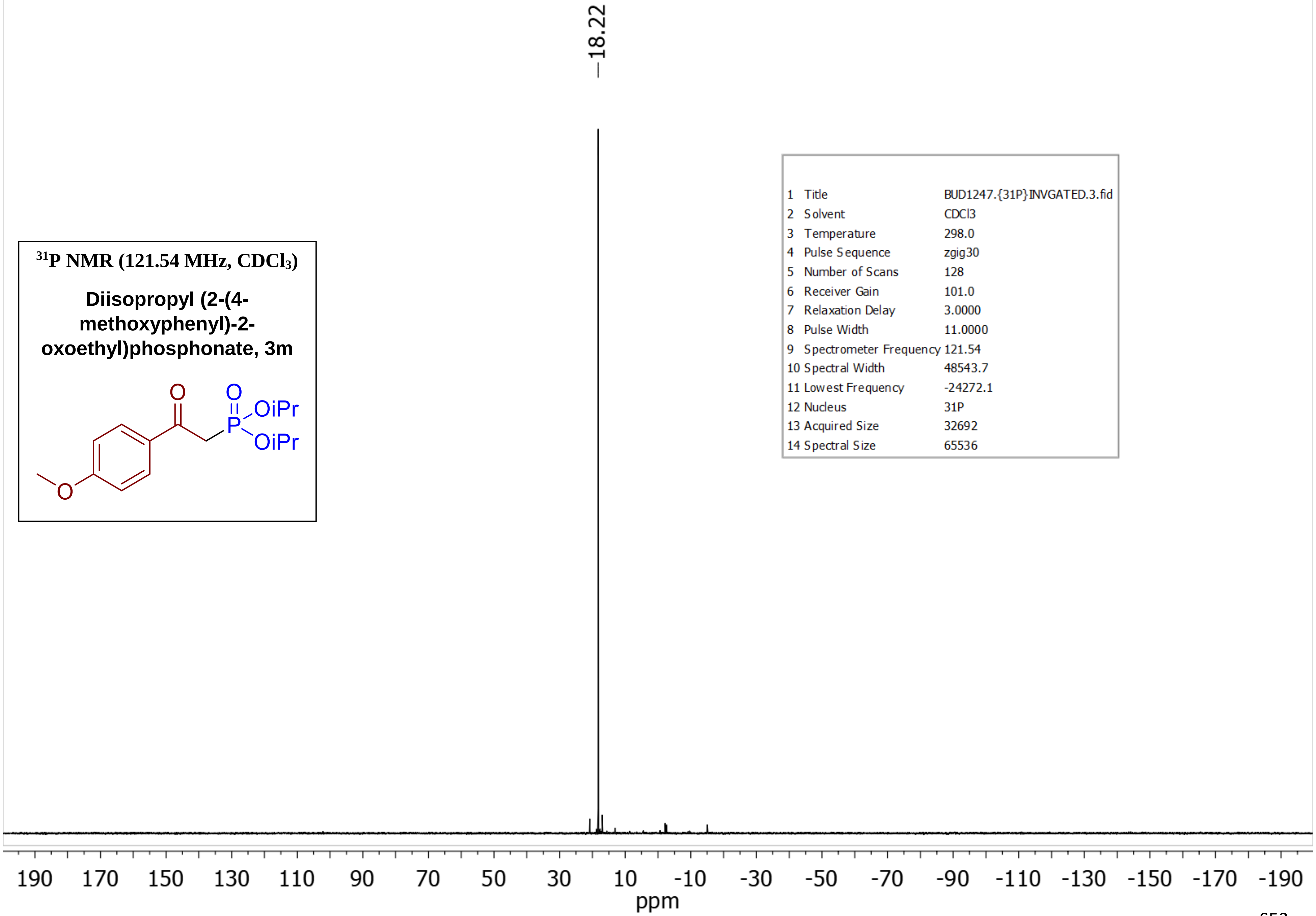

1	Title	BUD1247.{31P}INVGATED.3.fid
2	Solvent	CDCl3
3	Temperature	298.0
4	Pulse Sequence	zgig30
5	Number of Scans	128
6	Receiver Gain	101.0
7	Relaxation Delay	3.0000
8	Pulse Width	11.0000
9	Spectrometer Frequency	121.54
10	Spectral Width	48543.7
11	Lowest Frequency	-24272.1
12	Nucleus	31P
13	Acquired Size	32692
14	Spectral Size	65536

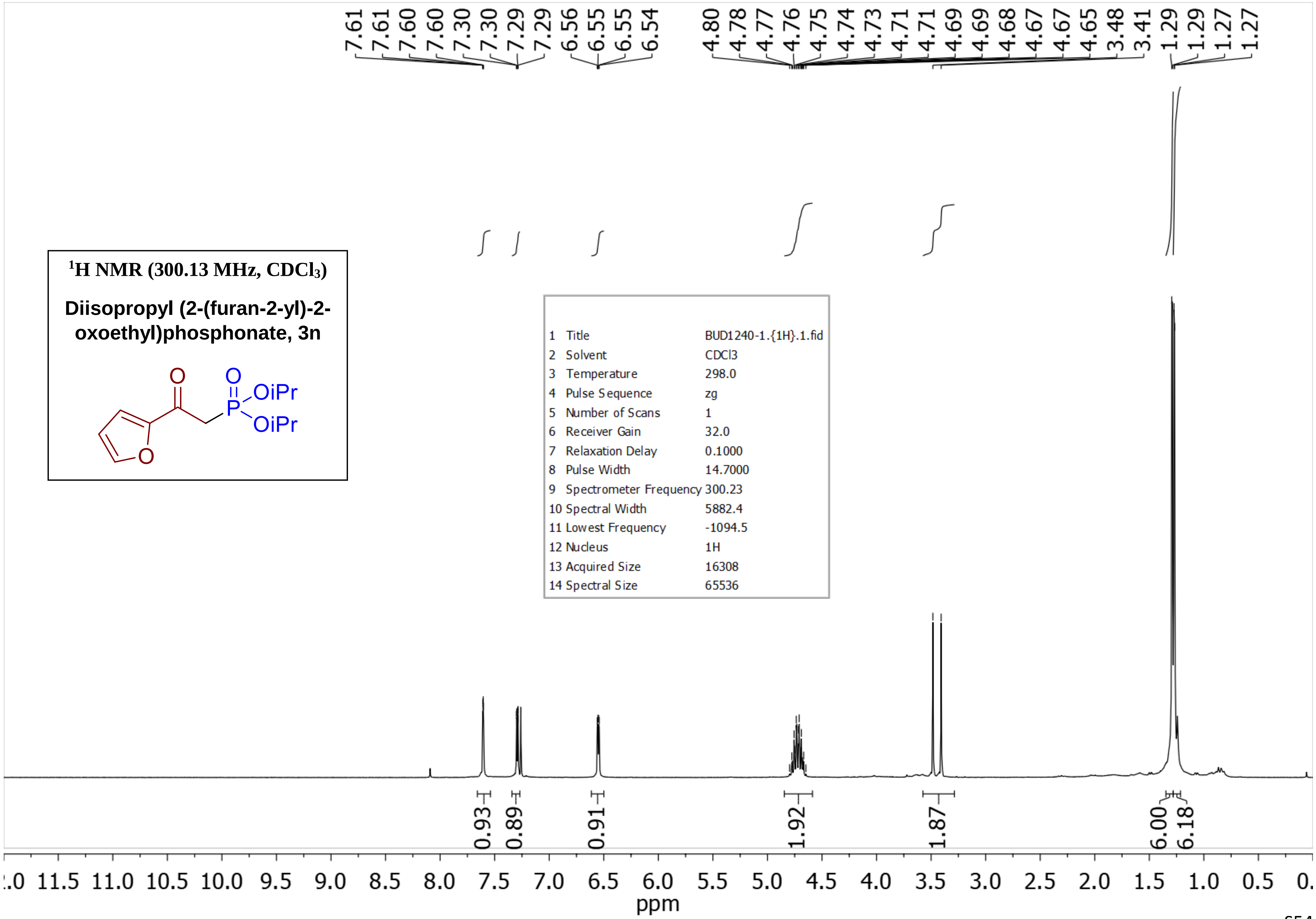

1H NMR (300.13 MHz, CDCl3)
Diisopropyl (2-(furan-2-yl)-2-oxoethyl)phosphonate, 3n
O
O
OiPr
OiPr
P
O
7.61 7.61 7.60 7.60 7.30 7.30 7.29 7.29 6.56 6.55 6.55 6.54
4.80 4.78 4.77 4.76 4.75 4.74 4.73 4.71 4.71 4.69 4.69 4.68 4.67 4.67 4.65 3.48 3.41 1.29 1.29 1.27 1.27
1 Title BUD1240-1.{1H}.1.fid
2 Solvent CDCl3
3 Temperature 298.0
4 Pulse Sequence zg
5 Number of Scans 1
6 Receiver Gain 32.0
7 Relaxation Delay 0.1000
8 Pulse Width 14.7000
9 Spectrometer Frequency 300.23
10 Spectral Width 5882.4
11 Lowest Frequency -1094.5
12 Nucleus 1H
13 Acquired Size 16308
14 Spectral Size 65536
0.93 0.89 0.91 1.92 1.87 6.00 6.18
11.5 11.0 10.5 10.0 9.5 9.0 8.5 8.0 7.5 7.0 6.5 6.0 5.5 5.0 4.5 4.0 3.5 3.0 2.5 2.0 1.5 1.0 0.5
ppm

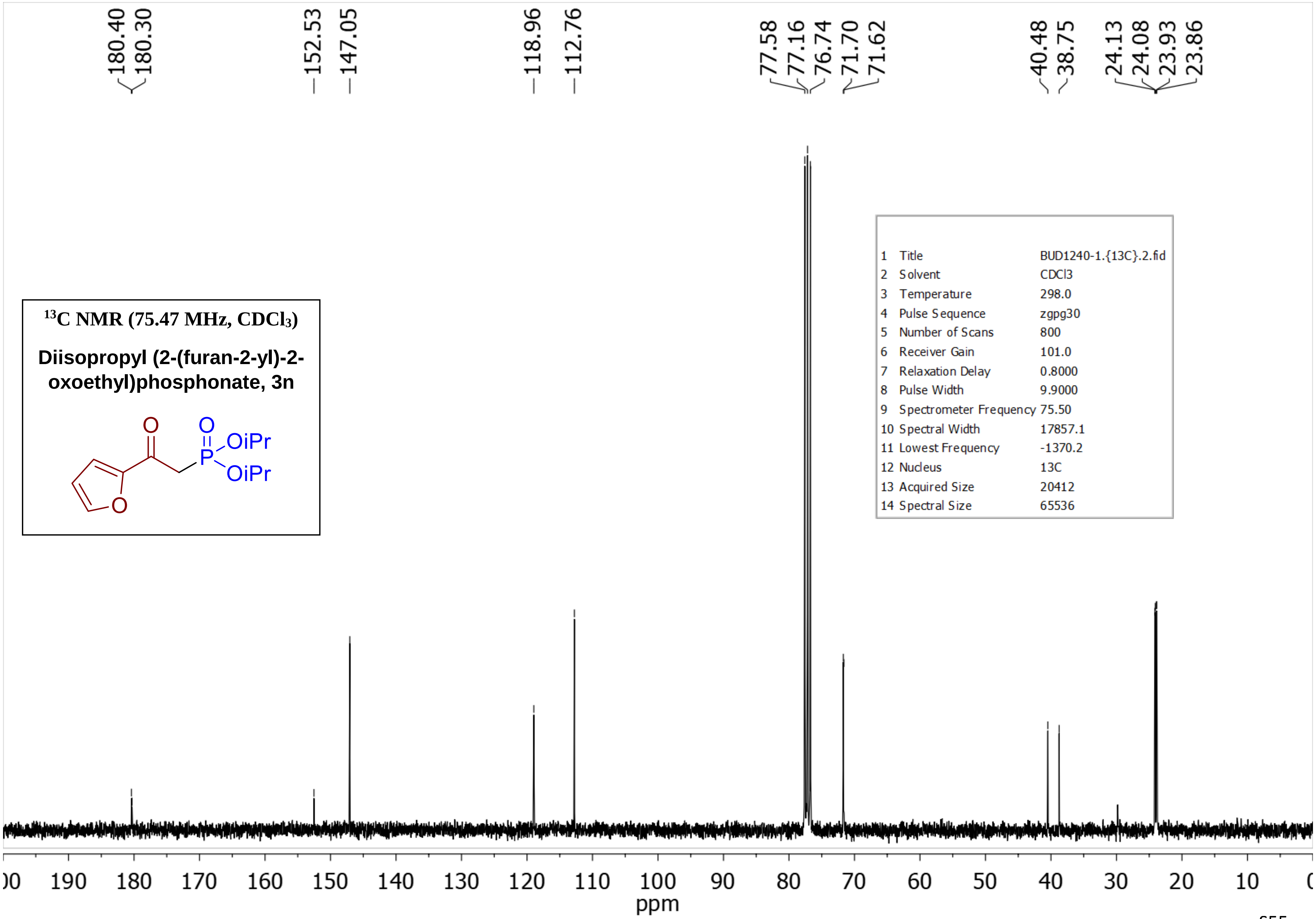

1	Title	BUD1240-1.{13C}.2.fid
2	Solvent	CDCl3
3	Temperature	298.0
4	Pulse Sequence	zgpg30
5	Number of Scans	800
6	Receiver Gain	101.0
7	Relaxation Delay	0.8000
8	Pulse Width	9.9000
9	Spectrometer Frequency	75.50
10	Spectral Width	17857.1
11	Lowest Frequency	-1370.2
12	Nucleus	13C
13	Acquired Size	20412
14	Spectral Size	65536

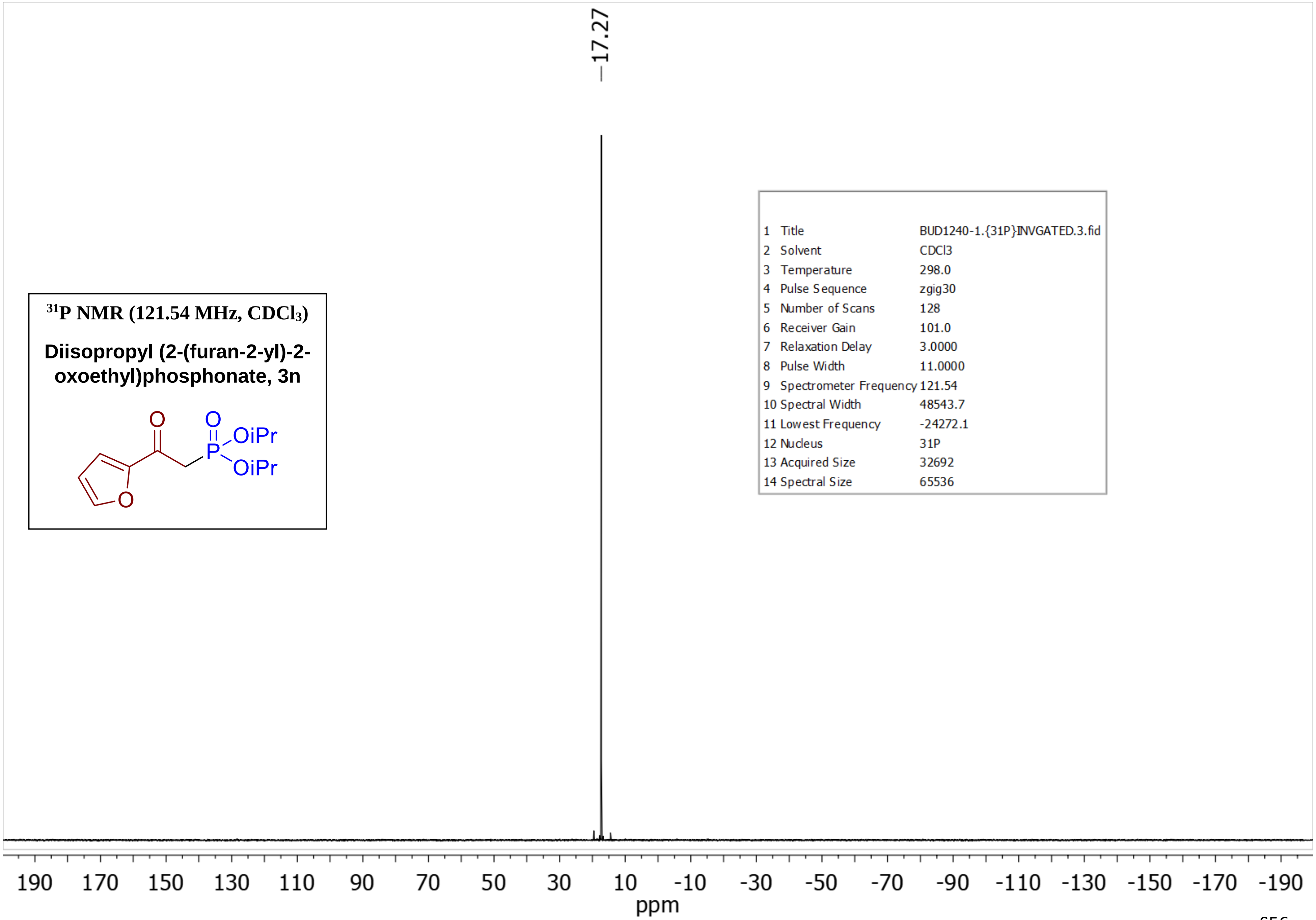

—17.27
³¹P NMR (121.54 MHz, CDCl₃)
Diisopropyl (2-(furan-2-yl)-2-oxoethyl)phosphonate, 3n
O
O
OiPr
P
OiPr
O
1 Title BUD1240-1.{31P}INVGATED.3.fid
2 Solvent CDCl3
3 Temperature 298.0
4 Pulse Sequence zgig30
5 Number of Scans 128
6 Receiver Gain 101.0
7 Relaxation Delay 3.0000
8 Pulse Width 11.0000
9 Spectrometer Frequency 121.54
10 Spectral Width 48543.7
11 Lowest Frequency -24272.1
12 Nucleus 31P
13 Acquired Size 32692
14 Spectral Size 65536
190 170 150 130 110 90 70 50 30 10 -10 -30 -50 -70 -90 -110 -130 -150 -170 -190
ppm

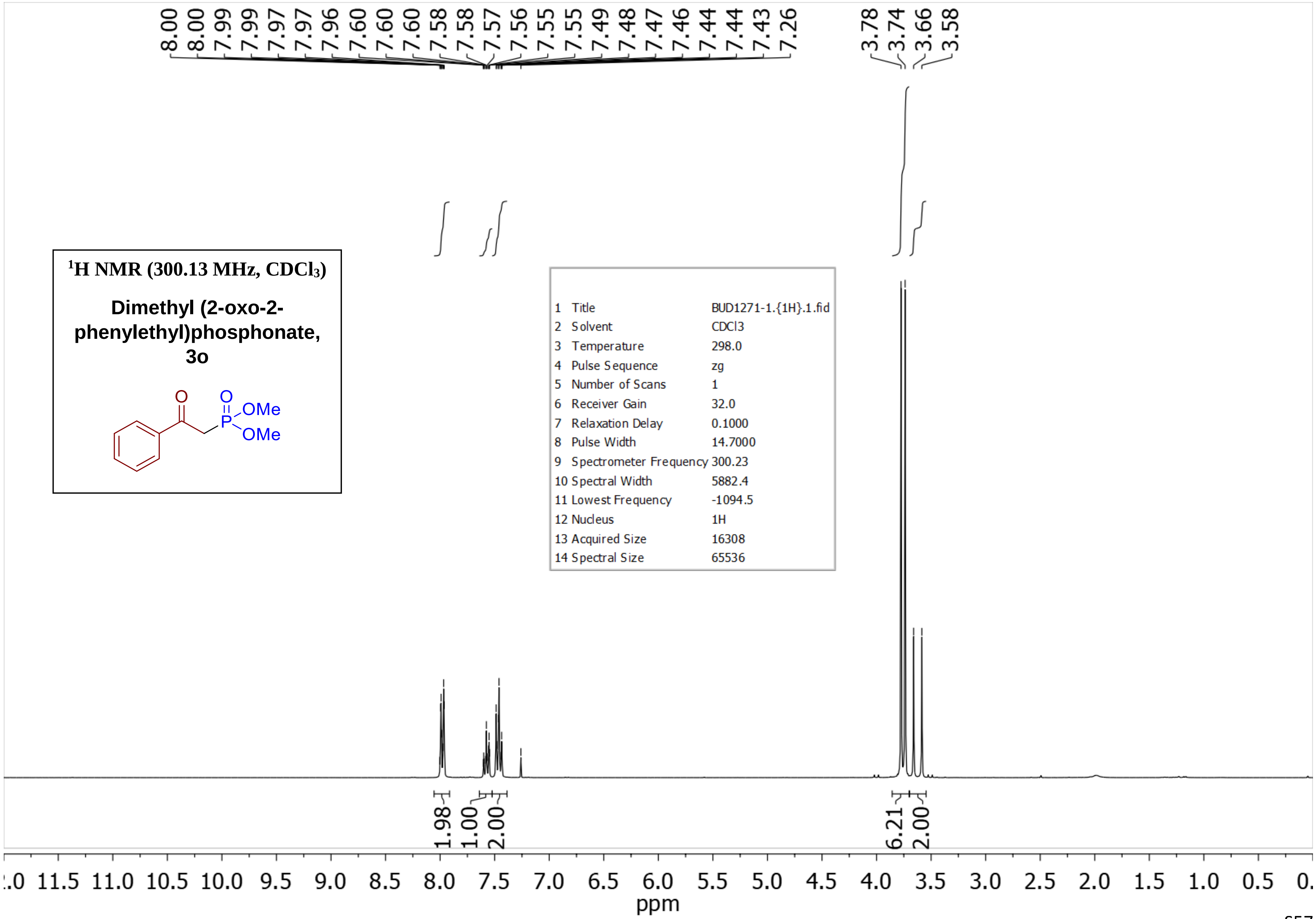

8.00 8.00 7.99 7.99 7.97 7.97 7.96 7.60 7.60 7.60 7.58 7.58 7.57 7.56 7.55 7.55 7.49 7.48 7.47 7.46 7.44 7.44 7.43 7.26
3.78 3.74 3.66 3.58
1H NMR (300.13 MHz, CDCl3)
Dimethyl (2-oxo-2-phenylethyl)phosphonate, 3o
O
O
P
OMe
OMe
1 Title BUD1271-1.{1H}.1.fid
2 Solvent CDCl3
3 Temperature 298.0
4 Pulse Sequence zg
5 Number of Scans 1
6 Receiver Gain 32.0
7 Relaxation Delay 0.1000
8 Pulse Width 14.7000
9 Spectrometer Frequency 300.23
10 Spectral Width 5882.4
11 Lowest Frequency -1094.5
12 Nucleus 1H
13 Acquired Size 16308
14 Spectral Size 65536
1.98
1.00
2.00
6.21
2.00
11.5 11.0 10.5 10.0 9.5 9.0 8.5 8.0 7.5 7.0 6.5 6.0 5.5 5.0 4.5 4.0 3.5 3.0 2.5 2.0 1.5 1.0 0.5
ppm

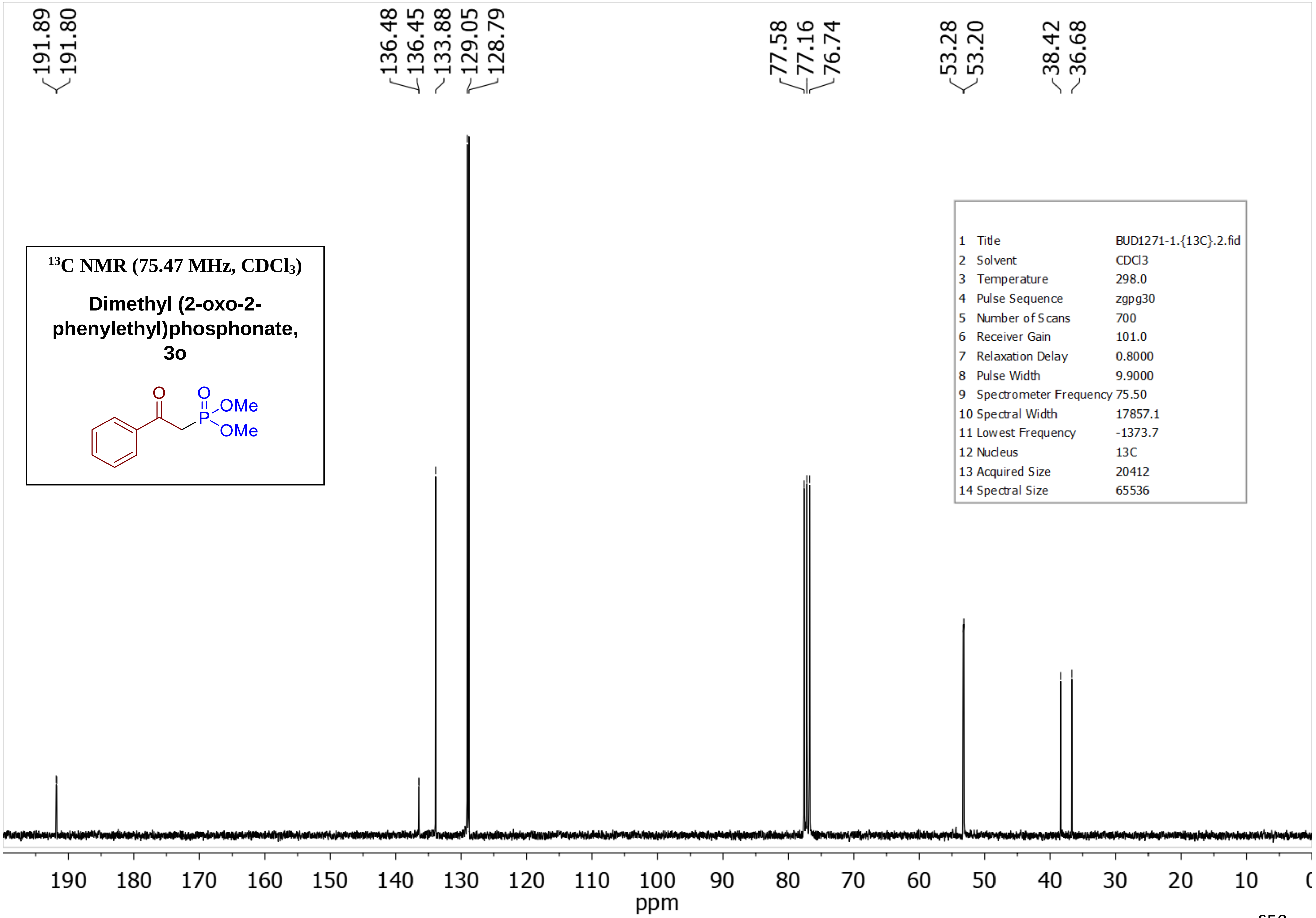
191.89
191.80
136.48
136.45
133.88
129.05
128.79
77.58
77.16
76.74
53.28
53.20
38.42
36.68
13C NMR (75.47 MHz, CDCl3)
Dimethyl (2-oxo-2-phenylethyl)phosphonate, 3o
O
O
P
OMe
OMe
1 Title BUD1271-1.{13C}.2.fid
2 Solvent CDCl3
3 Temperature 298.0
4 Pulse Sequence zgpg30
5 Number of Scans 700
6 Receiver Gain 101.0
7 Relaxation Delay 0.8000
8 Pulse Width 9.9000
9 Spectrometer Frequency 75.50
10 Spectral Width 17857.1
11 Lowest Frequency -1373.7
12 Nucleus 13C
13 Acquired Size 20412
14 Spectral Size 65536
190
180
170
160
150
140
130
120
110
100
90
80
70
60
50
40
30
20
10
ppm

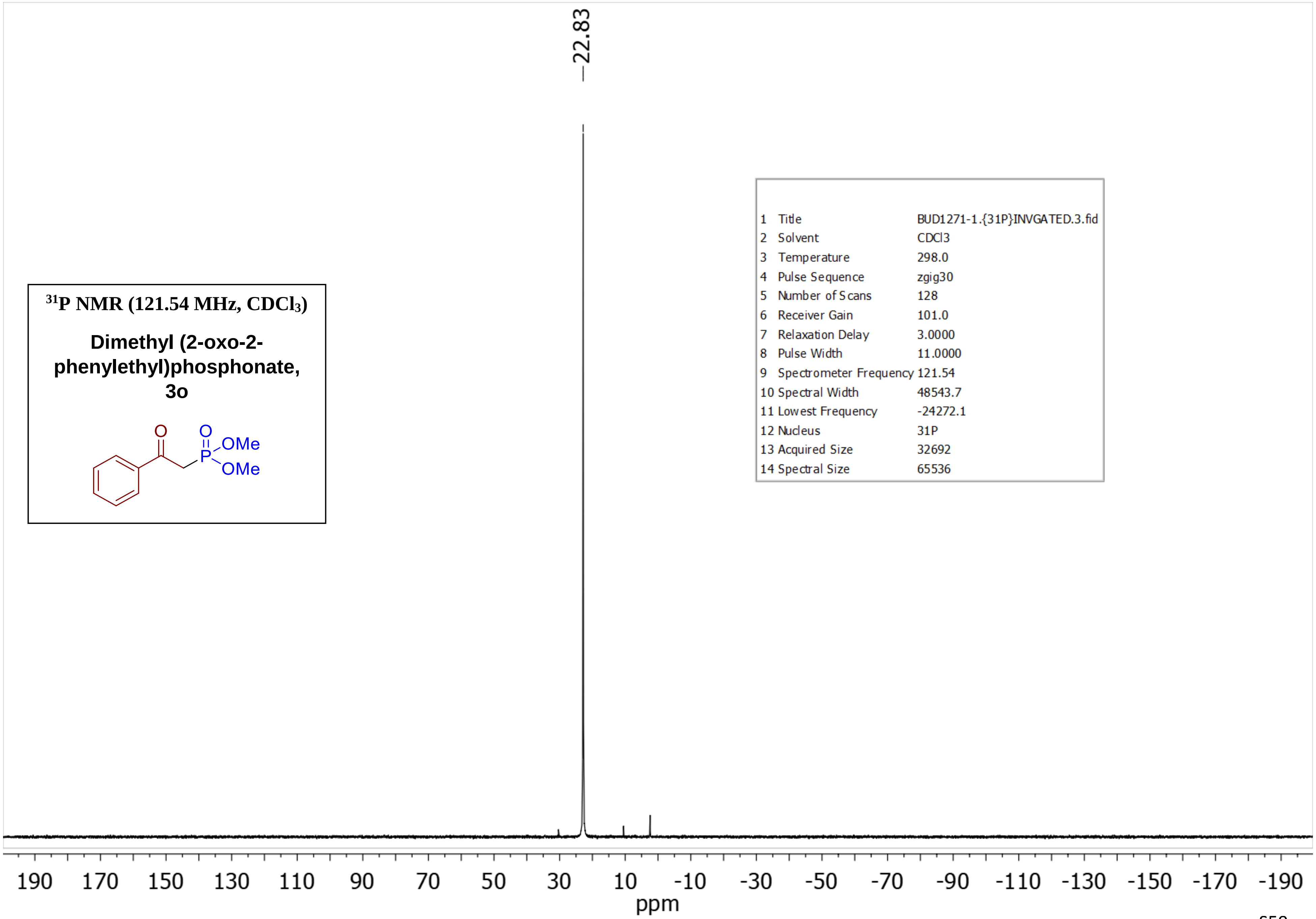

22.83
31P NMR (121.54 MHz, CDCl3)
Dimethyl (2-oxo-2-phenylethyl)phosphonate, 3o
O
O
P
OMe
OMe
1 Title BUD1271-1.{31P}INVGATED.3.fid
2 Solvent CDCl3
3 Temperature 298.0
4 Pulse Sequence zgig30
5 Number of Scans 128
6 Receiver Gain 101.0
7 Relaxation Delay 3.0000
8 Pulse Width 11.0000
9 Spectrometer Frequency 121.54
10 Spectral Width 48543.7
11 Lowest Frequency -24272.1
12 Nucleus 31P
13 Acquired Size 32692
14 Spectral Size 65536
190 170 150 130 110 90 70 50 30 10 -10 -30 -50 -70 -90 -110 -130 -150 -170 -190
ppm

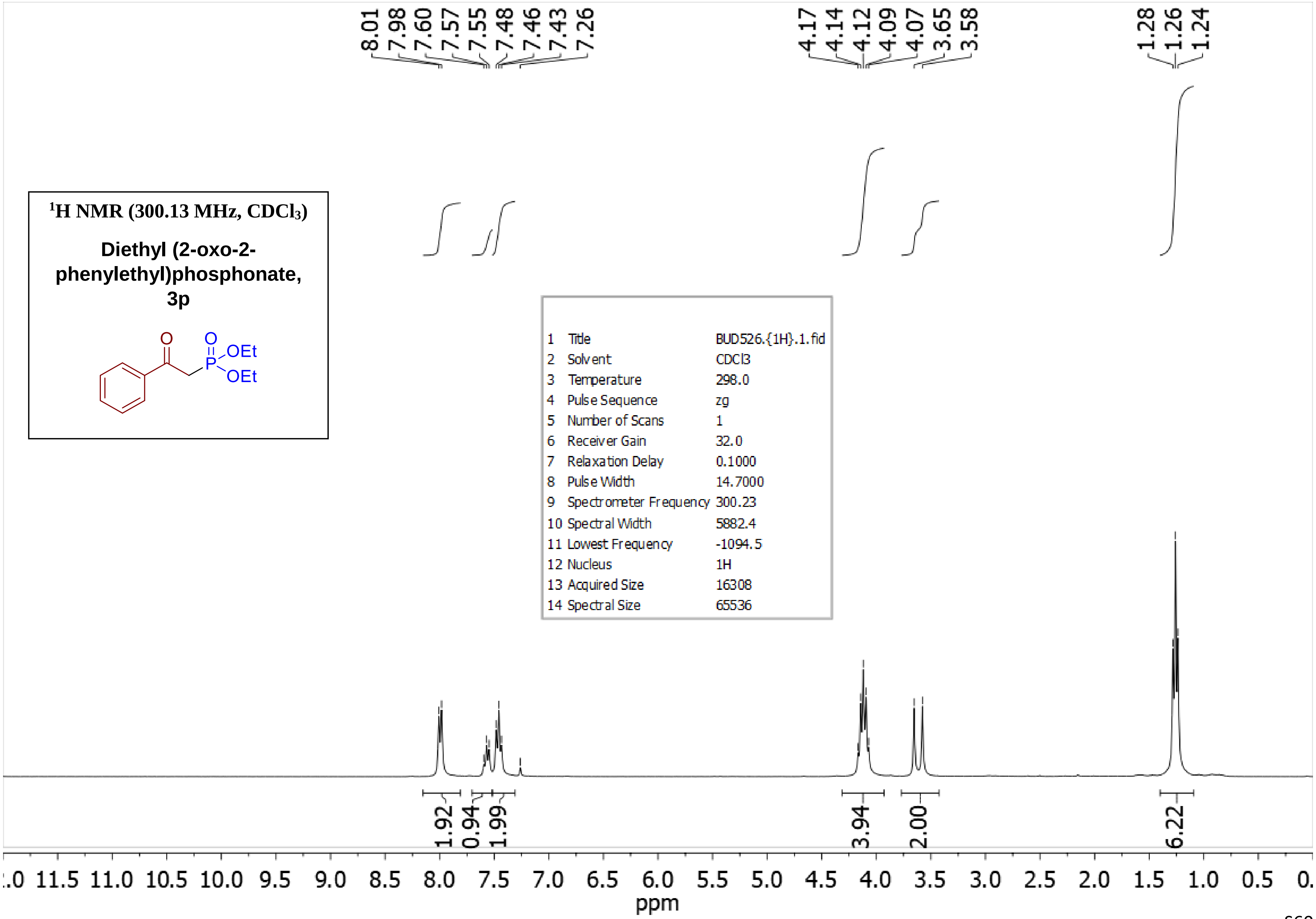

1H NMR (300.13 MHz, CDCl3)
Diethyl (2-oxo-2-phenylethyl)phosphonate, 3p
O
O
P
OEt
OEt
8.01 7.98 7.60 7.57 7.55 7.48 7.46 7.43 7.26
4.17 4.14 4.12 4.09 4.07 3.65 3.58
1.28 1.26 1.24
1 Title BUD526.{1H}.1.fid
2 Solvent CDCl3
3 Temperature 298.0
4 Pulse Sequence zg
5 Number of Scans 1
6 Receiver Gain 32.0
7 Relaxation Delay 0.1000
8 Pulse Width 14.7000
9 Spectrometer Frequency 300.23
10 Spectral Width 5882.4
11 Lowest Frequency -1094.5
12 Nucleus 1H
13 Acquired Size 16308
14 Spectral Size 65536
1.92 0.94 1.99 3.94 2.00 6.22
11.5 11.0 10.5 10.0 9.5 9.0 8.5 8.0 7.5 7.0 6.5 6.0 5.5 5.0 4.5 4.0 3.5 3.0 2.5 2.0 1.5 1.0 0.5
ppm

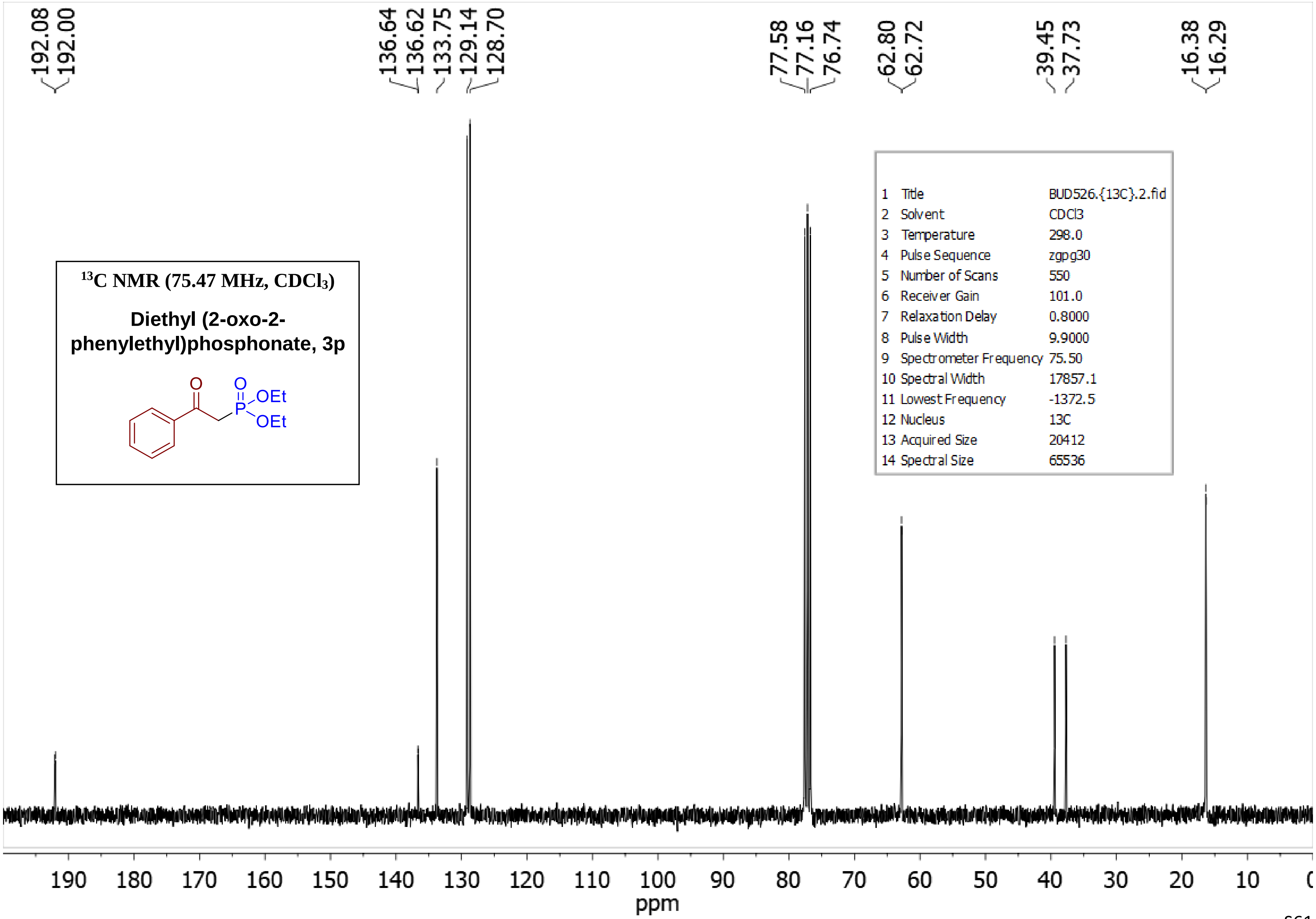
192.08
192.00
136.64
136.62
133.75
129.14
128.70
77.58
77.16
76.74
62.80
62.72
39.45
37.73
16.38
16.29
13C NMR (75.47 MHz, CDCl3)
Diethyl (2-oxo-2-phenylethyl)phosphonate, 3p
O
O
P
OEt
OEt
1 Title BUD526.{13C}.2.fid
2 Solvent CDCl3
3 Temperature 298.0
4 Pulse Sequence zgpg30
5 Number of Scans 550
6 Receiver Gain 101.0
7 Relaxation Delay 0.8000
8 Pulse Width 9.9000
9 Spectrometer Frequency 75.50
10 Spectral Width 17857.1
11 Lowest Frequency -1372.5
12 Nucleus 13C
13 Acquired Size 20412
14 Spectral Size 65536
190 180 170 160 150 140 130 120 110 100 90 80 70 60 50 40 30 20 10
ppm

^{31}P NMR (121.49 MHz, $CDCl_3$)

Diethyl (2-oxo-2-phenylethyl)phosphonate, 3p

1	Title	BUD526.{31P}INVGATED.3.fid
2	Solvent	CDCl3
3	Temperature	298.0
4	Pulse Sequence	zgig30
5	Number of Scans	128
6	Receiver Gain	101.0
7	Relaxation Delay	3.0000
8	Pulse Width	11.0000
9	Spectrometer Frequency	121.54
10	Spectral Width	48543.7
11	Lowest Frequency	-24272.1
12	Nucleus	31P
13	Acquired Size	32700
14	Spectral Size	65536

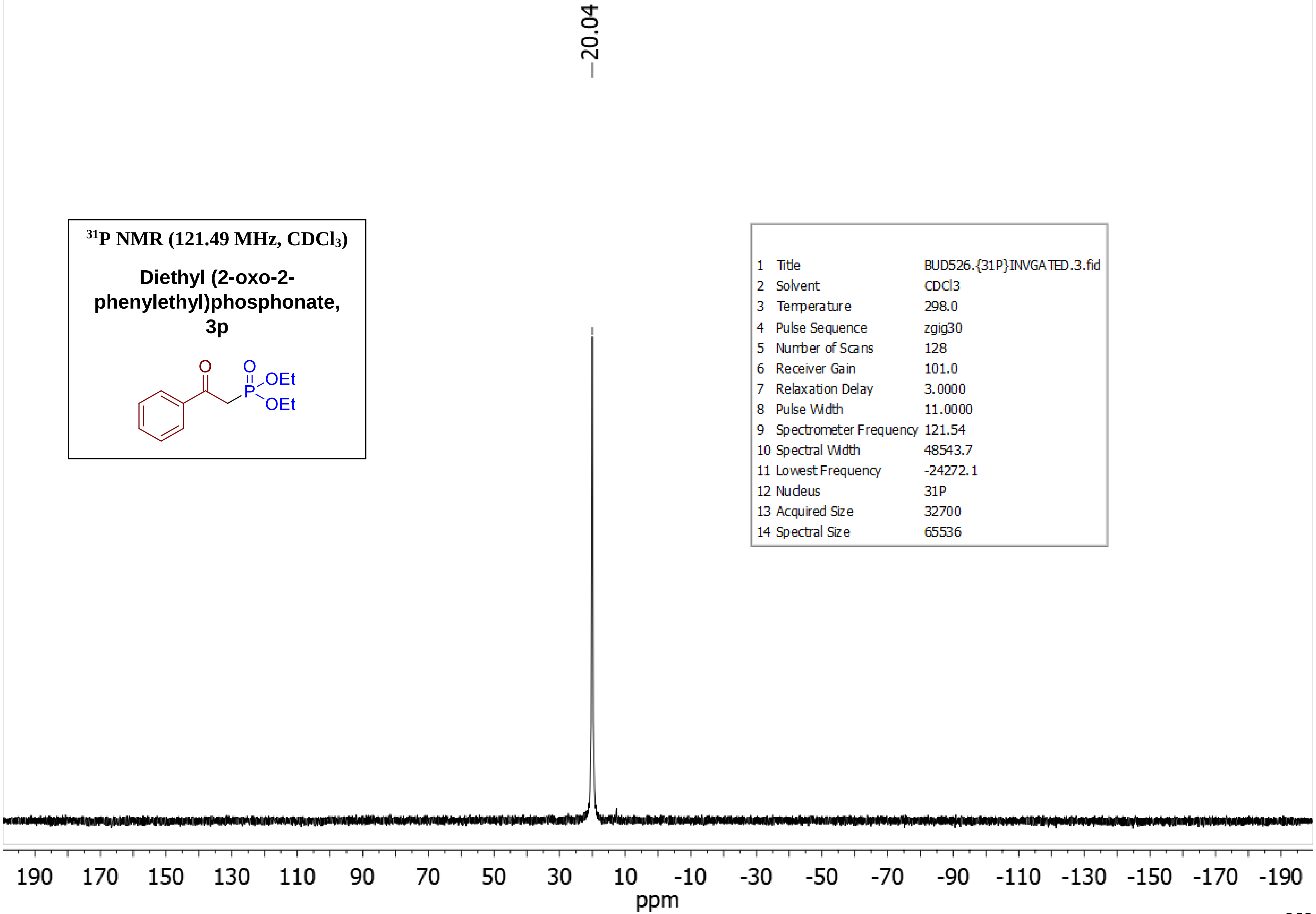

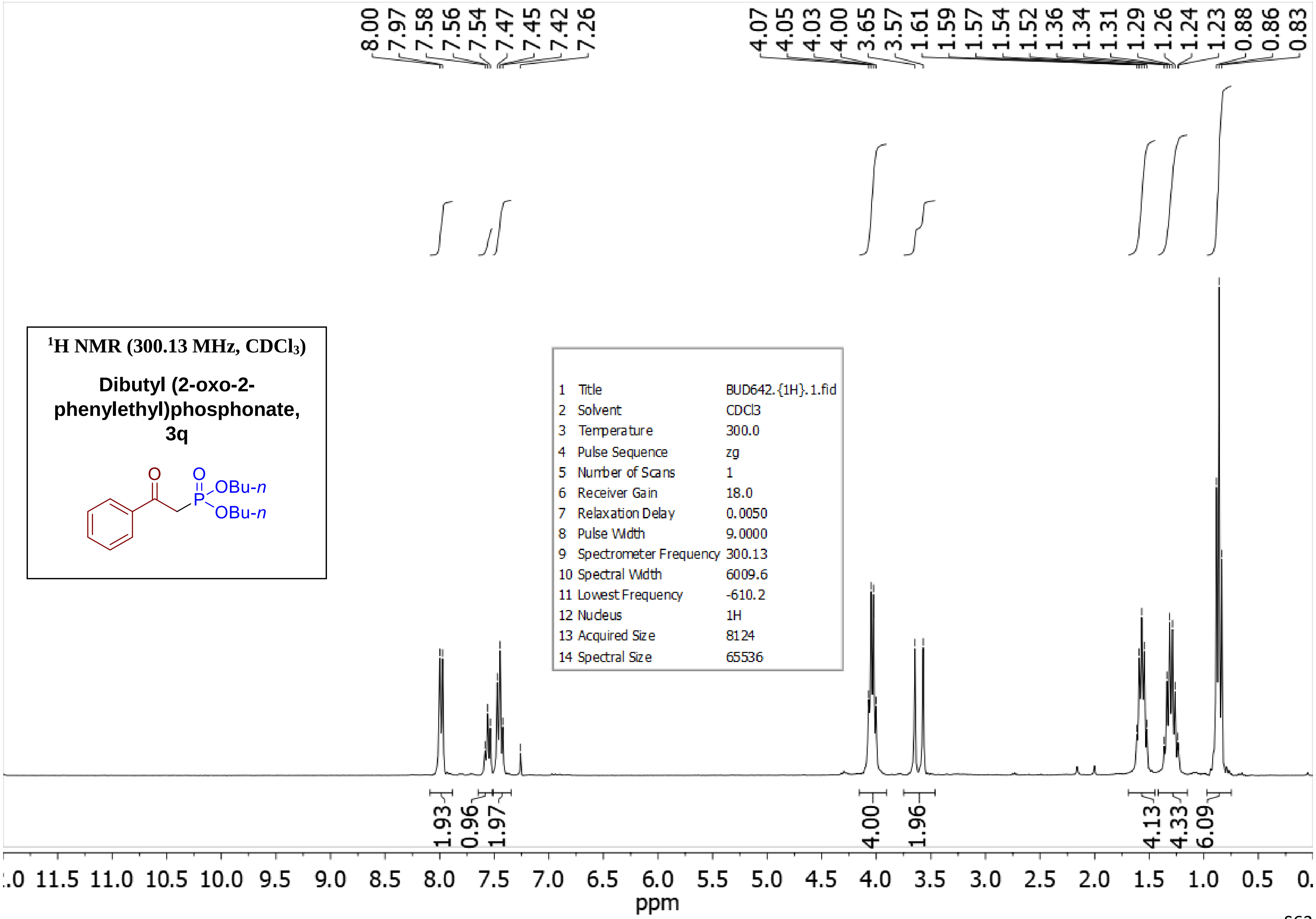

^{1}H NMR (300.13 MHz, $CDCl_3$)

Dibutyl (2-oxo-2-phenylethyl)phosphonate, 3q

1	Title	BUD642.{1H}.1.fid
2	Solvent	CDCl3
3	Temperature	300.0
4	Pulse Sequence	zg
5	Number of Scans	1
6	Receiver Gain	18.0
7	Relaxation Delay	0.0050
8	Pulse Width	9.0000
9	Spectrometer Frequency	300.13
10	Spectral Width	6009.6
11	Lowest Frequency	-610.2
12	Nucleus	1H
13	Acquired Size	8124
14	Spectral Size	65536

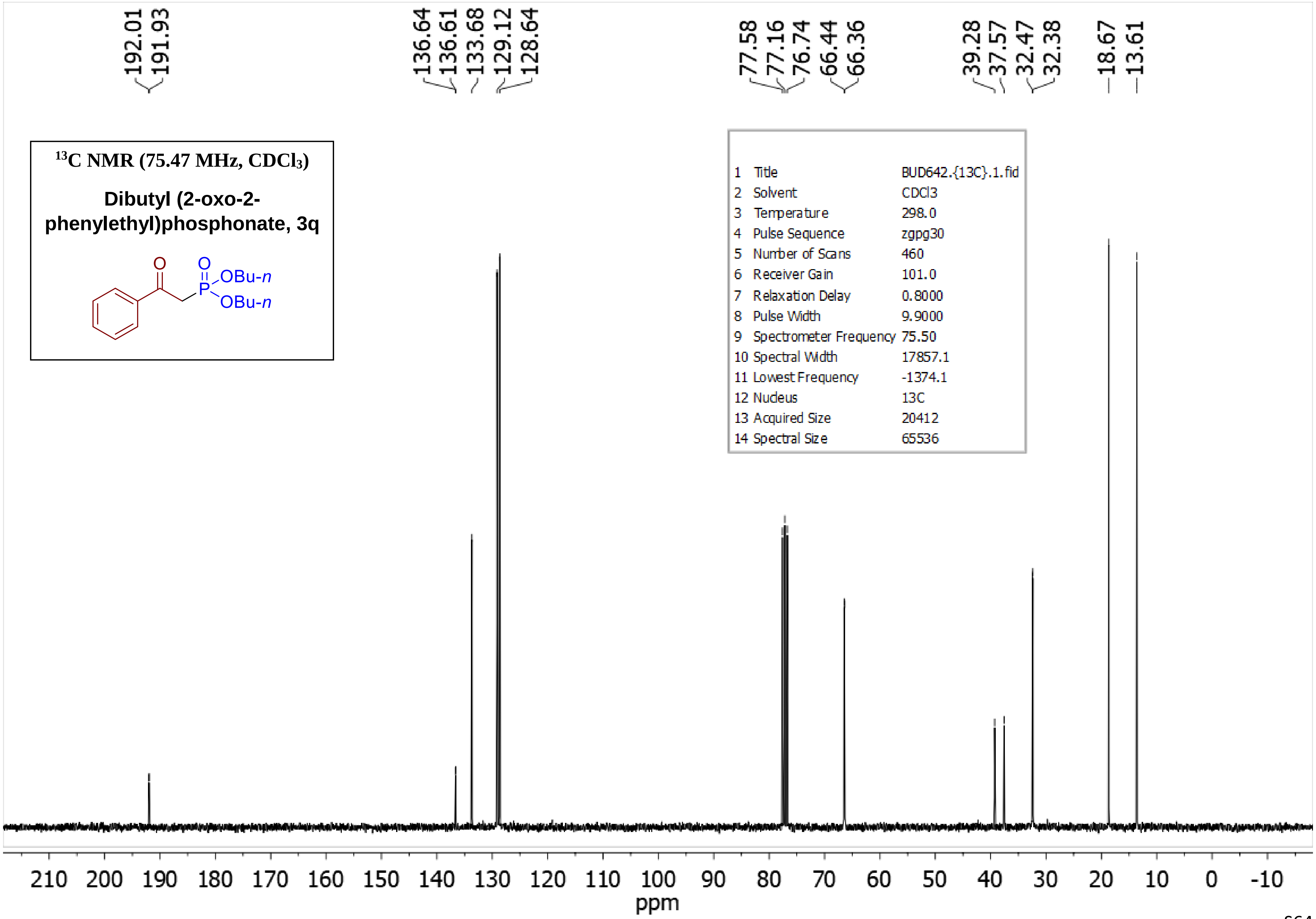
192.01
191.93
136.64
136.61
133.68
129.12
128.64
77.58
77.16
76.74
66.44
66.36
39.28
37.57
32.47
32.38
18.67
13.61
13C NMR (75.47 MHz, CDCl3)
Dibutyl (2-oxo-2-phenylethyl)phosphonate, 3q
O
O
P
OBu-n
OBu-n
1 Title BUD642.{13C}.1.fid
2 Solvent CDCl3
3 Temperature 298.0
4 Pulse Sequence zgpg30
5 Number of Scans 460
6 Receiver Gain 101.0
7 Relaxation Delay 0.8000
8 Pulse Width 9.9000
9 Spectrometer Frequency 75.50
10 Spectral Width 17857.1
11 Lowest Frequency -1374.1
12 Nucleus 13C
13 Acquired Size 20412
14 Spectral Size 65536
210 200 190 180 170 160 150 140 130 120 110 100 90 80 70 60 50 40 30 20 10 0 -10
ppm

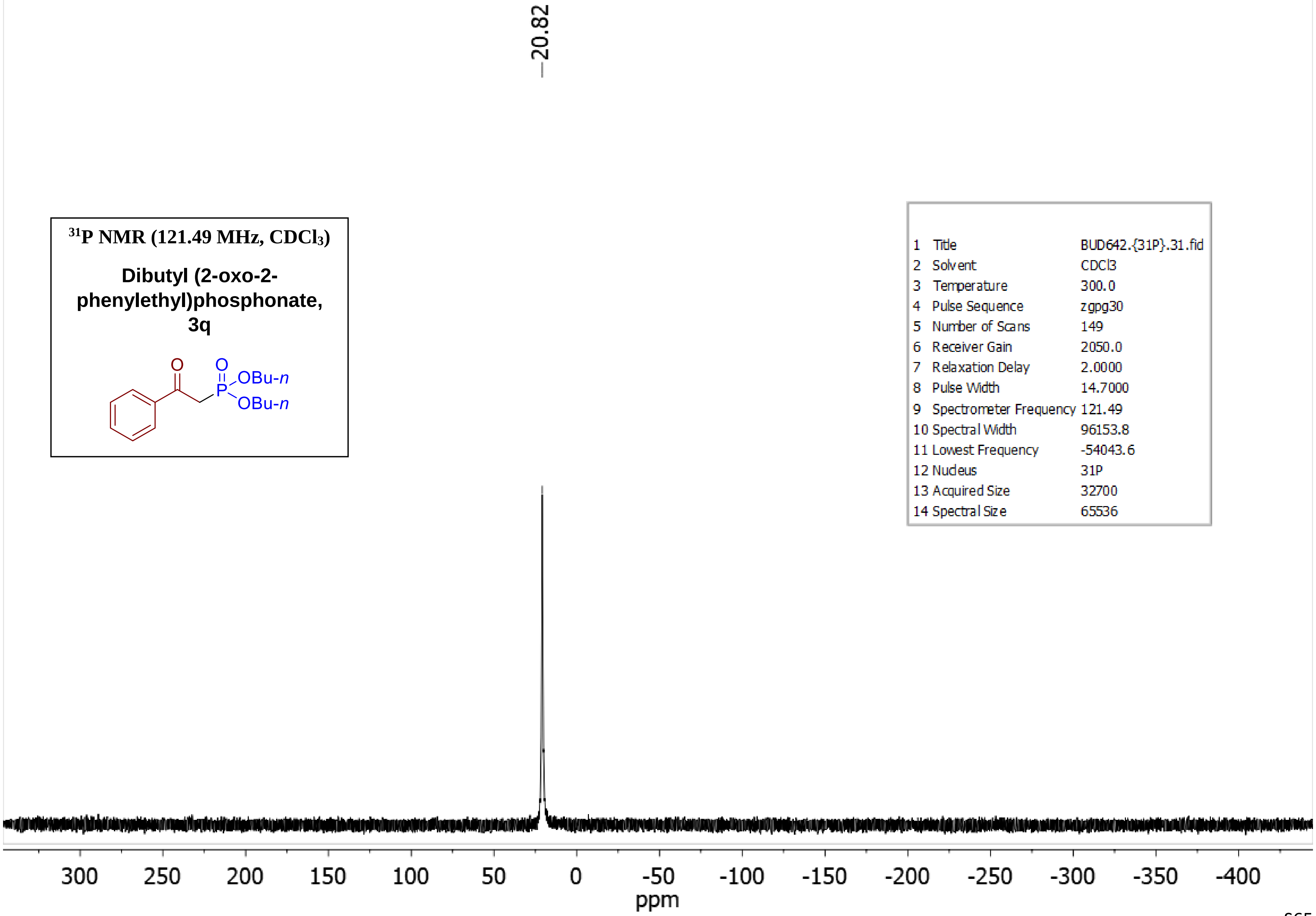

1	Title	BUD642.{31P}.31.fid
2	Solvent	CDCl3
3	Temperature	300.0
4	Pulse Sequence	zgpg30
5	Number of Scans	149
6	Receiver Gain	2050.0
7	Relaxation Delay	2.0000
8	Pulse Width	14.7000
9	Spectrometer Frequency	121.49
10	Spectral Width	96153.8
11	Lowest Frequency	-54043.6
12	Nucleus	31P
13	Acquired Size	32700
14	Spectral Size	65536

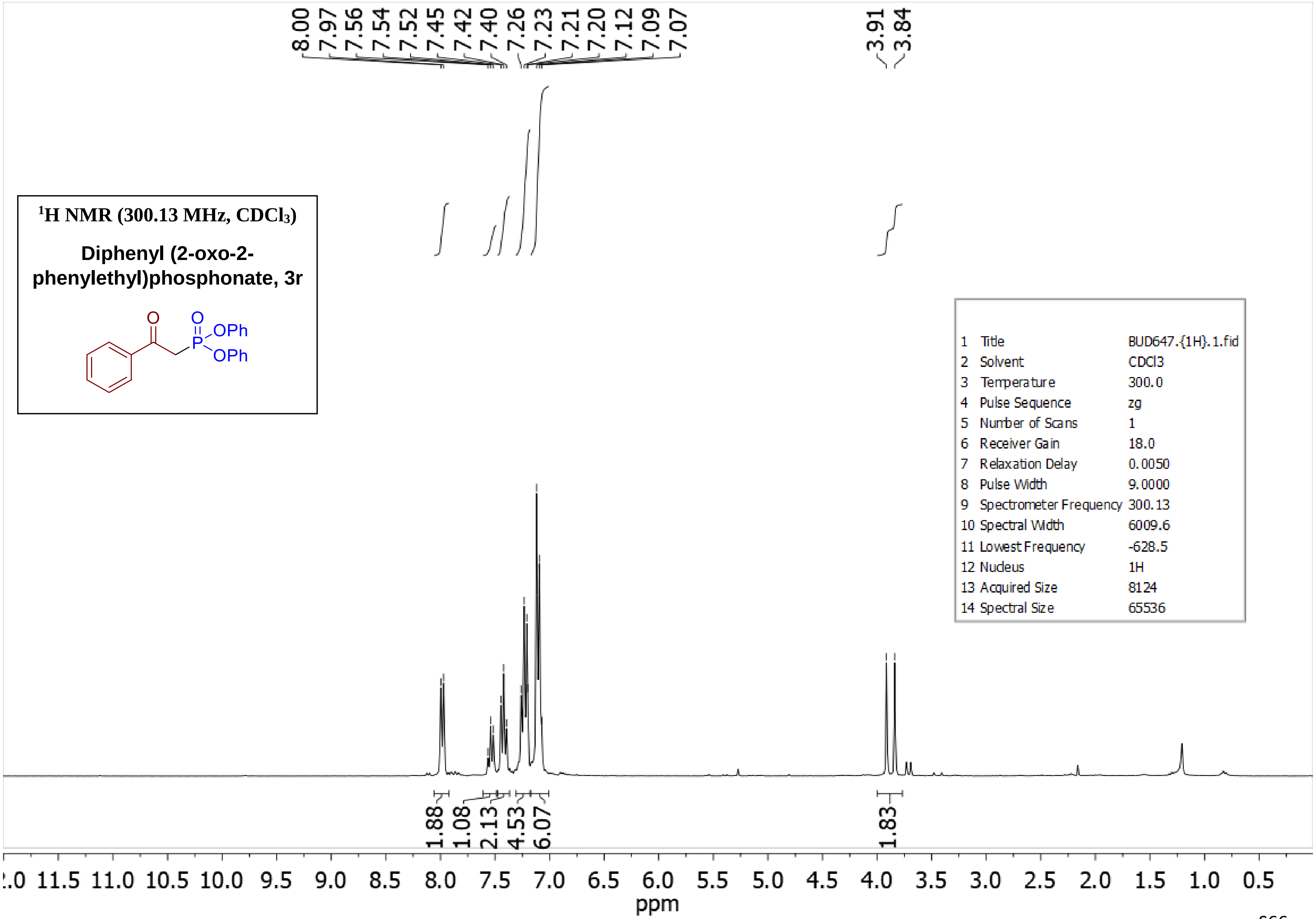

8.00
7.97
7.56
7.54
7.52
7.45
7.42
7.40
7.26
7.23
7.21
7.20
7.12
7.09
7.07
3.91
3.84
1H NMR (300.13 MHz, CDCl3)
Diphenyl (2-oxo-2-phenylethyl)phosphonate, 3r
O
O
P
OPh
OPh
1 Title BUD647.{1H}.1.fid
2 Solvent CDCl3
3 Temperature 300.0
4 Pulse Sequence zg
5 Number of Scans 1
6 Receiver Gain 18.0
7 Relaxation Delay 0.0050
8 Pulse Width 9.0000
9 Spectrometer Frequency 300.13
10 Spectral Width 6009.6
11 Lowest Frequency -628.5
12 Nucleus 1H
13 Acquired Size 8124
14 Spectral Size 65536
1.88
1.08
2.13
4.53
6.07
1.83
11.5 11.0 10.5 10.0 9.5 9.0 8.5 8.0 7.5 7.0 6.5 6.0 5.5 5.0 4.5 4.0 3.5 3.0 2.5 2.0 1.5 1.0 0.5
ppm

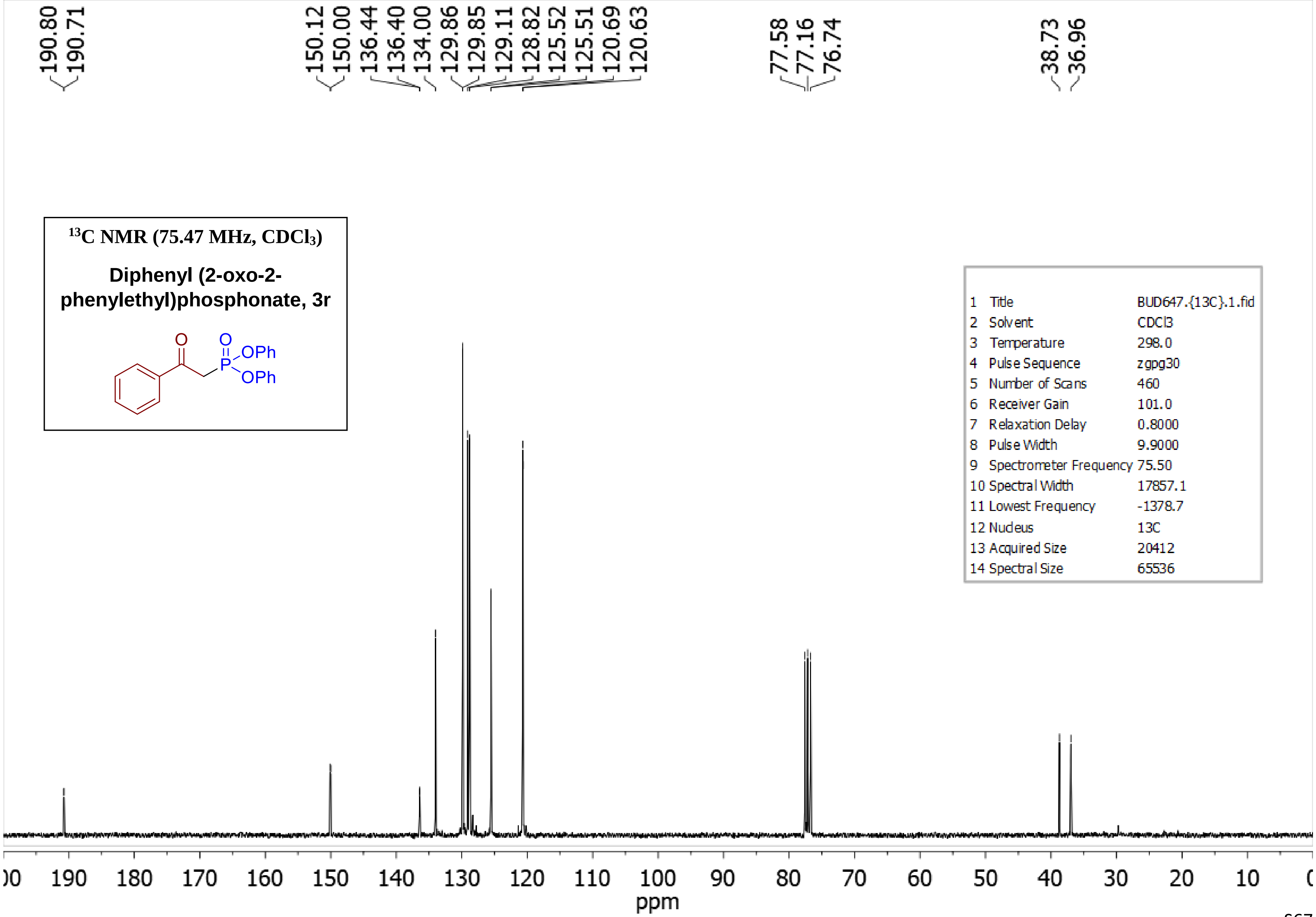
190.80
190.71
150.12
150.00
136.44
136.40
134.00
129.86
129.85
129.11
128.82
125.52
125.51
120.69
120.63
77.58
77.16
76.74
38.73
36.96
13C NMR (75.47 MHz, CDCl3)
Diphenyl (2-oxo-2-phenylethyl)phosphonate, 3r
O
O
P
OPh
OPh
1 Title BUD647.{13C}.1.fid
2 Solvent CDCl3
3 Temperature 298.0
4 Pulse Sequence zgpg30
5 Number of Scans 460
6 Receiver Gain 101.0
7 Relaxation Delay 0.8000
8 Pulse Width 9.9000
9 Spectrometer Frequency 75.50
10 Spectral Width 17857.1
11 Lowest Frequency -1378.7
12 Nucleus 13C
13 Acquired Size 20412
14 Spectral Size 65536
190
180
170
160
150
140
130
120
110
100
90
80
70
60
50
40
30
20
10
ppm

^{31}P NMR (121.49 MHz, $CDCl_3$)

Diphenyl (2-oxo-2-phenylethyl)phosphonate, 3r

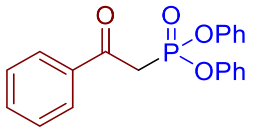

1	Title	BUD647.{31P}INVGATED.2.fid
2	Solvent	CDCl3
3	Temperature	298.0
4	Pulse Sequence	zgig30
5	Number of Scans	128
6	Receiver Gain	101.0
7	Relaxation Delay	3.0000
8	Pulse Width	11.0000
9	Spectrometer Frequency	121.54
10	Spectral Width	48543.7
11	Lowest Frequency	-24272.1
12	Nucleus	31P
13	Acquired Size	32700
14	Spectral Size	65536

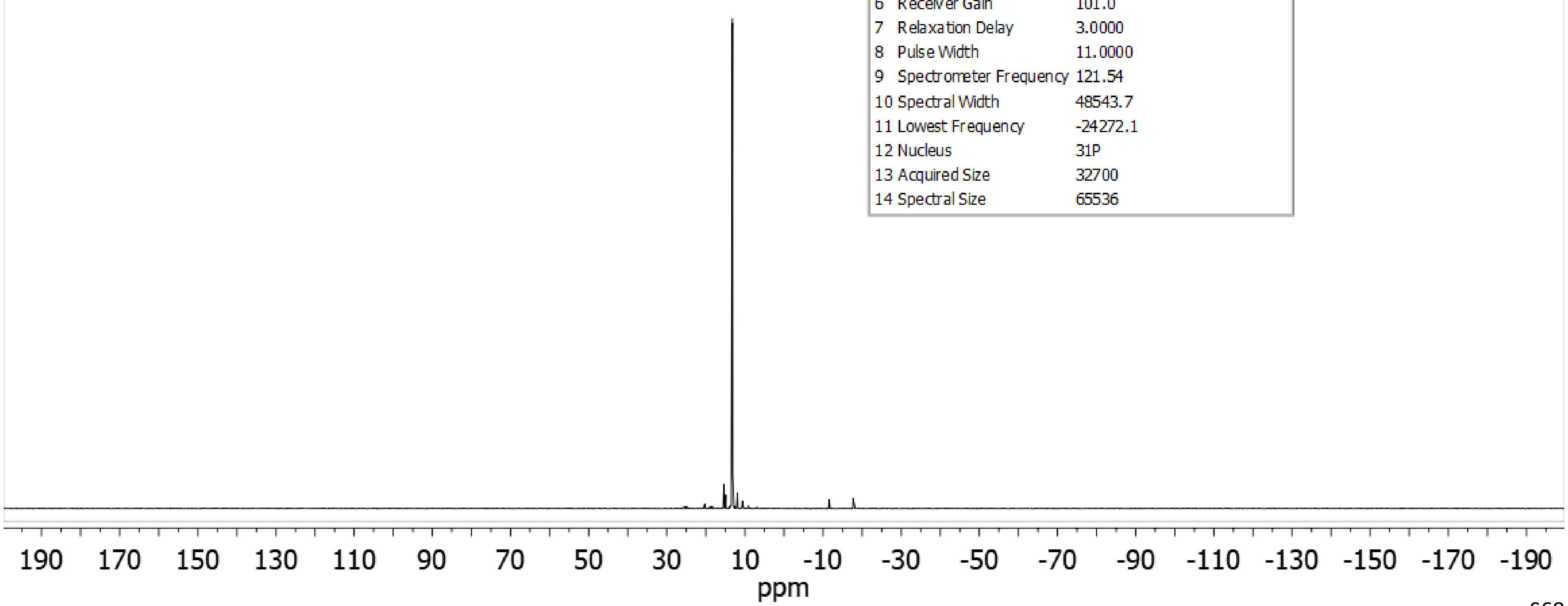

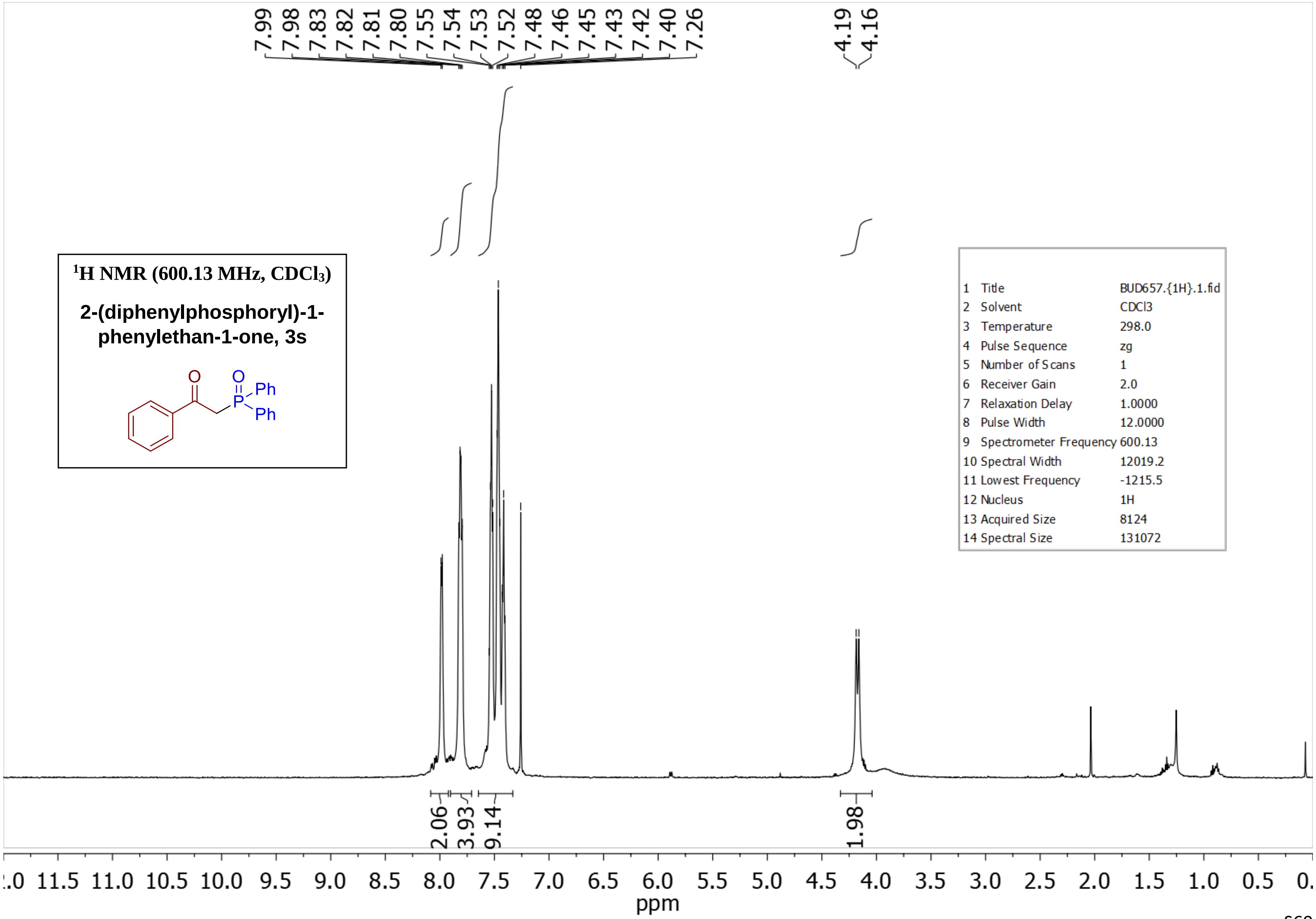

1H NMR (600.13 MHz, CDCl3)
2-(diphenylphosphoryl)-1-phenylethan-1-one, 3s
O
O
P
Ph
Ph
7.99
7.98
7.83
7.82
7.81
7.80
7.55
7.54
7.53
7.52
7.48
7.46
7.45
7.43
7.42
7.40
7.26
4.19
4.16
2.06
3.93
9.14
1.98
1 Title BUD657.{1H}.1.fid
2 Solvent CDCl3
3 Temperature 298.0
4 Pulse Sequence zg
5 Number of Scans 1
6 Receiver Gain 2.0
7 Relaxation Delay 1.0000
8 Pulse Width 12.0000
9 Spectrometer Frequency 600.13
10 Spectral Width 12019.2
11 Lowest Frequency -1215.5
12 Nucleus 1H
13 Acquired Size 8124
14 Spectral Size 131072
11.5
11.0
10.5
10.0
9.5
9.0
8.5
8.0
7.5
7.0
6.5
6.0
5.5
5.0
4.5
4.0
3.5
3.0
2.5
2.0
1.5
1.0
0.5
ppm

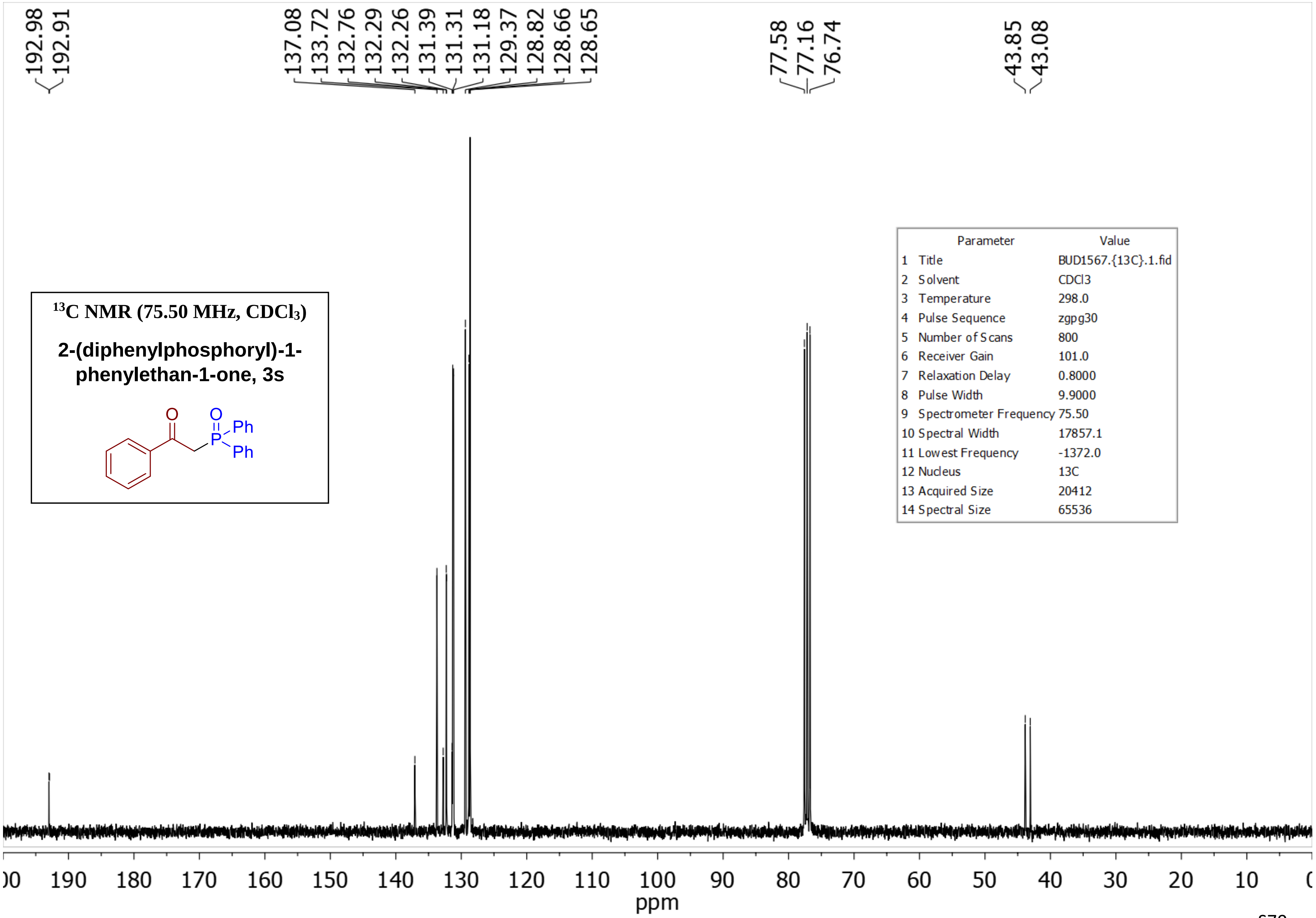
192.98
192.91
137.08
133.72
132.76
132.29
132.26
131.39
131.31
131.18
129.37
128.82
128.66
128.65
77.58
77.16
76.74
43.85
43.08
13C NMR (75.50 MHz, CDCl3)
2-(diphenylphosphoryl)-1-phenylethan-1-one, 3s
O
O
P
Ph
Ph
Parameter
Value
1 Title
BUD1567.{13C}.1.fid
2 Solvent
CDCl3
3 Temperature
298.0
4 Pulse Sequence
zgpg30
5 Number of Scans
800
6 Receiver Gain
101.0
7 Relaxation Delay
0.8000
8 Pulse Width
9.9000
9 Spectrometer Frequency
75.50
10 Spectral Width
17857.1
11 Lowest Frequency
-1372.0
12 Nucleus
13C
13 Acquired Size
20412
14 Spectral Size
65536
190
180
170
160
150
140
130
120
110
100
90
80
70
60
50
40
30
20
10
ppm

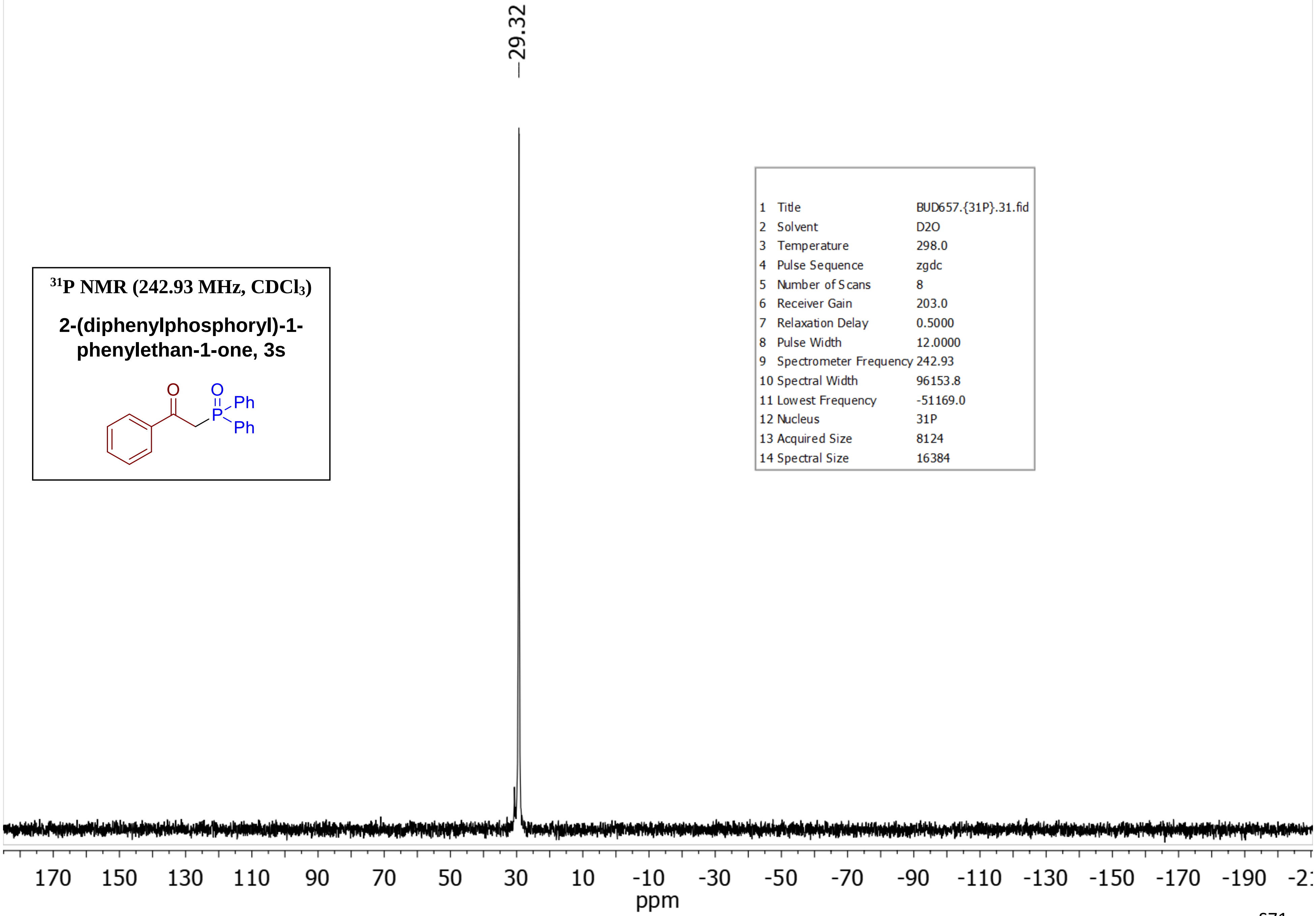

—29.32
^{31}P NMR (242.93 MHz, CDCl$_3$)
2-(diphenylphosphoryl)-1-phenylethan-1-one, 3s
O
O
P
Ph
Ph
1 Title BUD657.{31P}.31.fid
2 Solvent D2O
3 Temperature 298.0
4 Pulse Sequence zgdc
5 Number of Scans 8
6 Receiver Gain 203.0
7 Relaxation Delay 0.5000
8 Pulse Width 12.0000
9 Spectrometer Frequency 242.93
10 Spectral Width 96153.8
11 Lowest Frequency -51169.0
12 Nucleus 31P
13 Acquired Size 8124
14 Spectral Size 16384
170 150 130 110 90 70 50 30 10 -10 -30 -50 -70 -90 -110 -130 -150 -170 -190
ppm
